# Imaging brain tissue architecture across millimeter to nanometer scales

**DOI:** 10.1038/s41587-023-01911-8

**Published:** 2023-08-31

**Authors:** Julia M. Michalska, Julia Lyudchik, Philipp Velicky, Hana Štefaničková, Jake F. Watson, Alban Cenameri, Christoph Sommer, Nicole Amberg, Alessandro Venturino, Karl Roessler, Thomas Czech, Romana Höftberger, Sandra Siegert, Gaia Novarino, Peter Jonas, Johann G. Danzl

**Affiliations:** 1 Institute of Science and Technology Austria, Klosterneuburg, Austria; 2 Department of Neurology, Division of Neuropathology and Neurochemistry, Medical University of Vienna, Vienna, Austria; 3 Comprehensive Center for Clinical Neurosciences and Mental Health, Medical University of Vienna, Vienna, Austria; 4 Department of Neurosurgery, Medical University of Vienna, Vienna, Austria; 5 Present Address: Core Facility Imaging, Medical University of Vienna, Vienna, Austria

**Keywords:** Super-resolution microscopy, Fluorescence imaging, Cellular neuroscience, Synaptic transmission, 3-D reconstruction

## Abstract

Mapping the complex and dense arrangement of cells and their connectivity in brain tissue demands nanoscale spatial resolution imaging. Super-resolution optical microscopy excels at visualizing specific molecules and individual cells but fails to provide tissue context. Here we developed Comprehensive Analysis of Tissues across Scales (CATS), a technology to densely map brain tissue architecture from millimeter regional to nanometer synaptic scales in diverse chemically fixed brain preparations, including rodent and human. CATS uses fixation-compatible extracellular labeling and optical imaging, including stimulated emission depletion or expansion microscopy, to comprehensively delineate cellular structures. It enables three-dimensional reconstruction of single synapses and mapping of synaptic connectivity by identification and analysis of putative synaptic cleft regions. Applying CATS to the mouse hippocampal mossy fiber circuitry, we reconstructed and quantified the synaptic input and output structure of identified neurons. We furthermore demonstrate applicability to clinically derived human tissue samples, including formalin-fixed paraffin-embedded routine diagnostic specimens, for visualizing the cellular architecture of brain tissue in health and disease.

## Abstract (web summary)

Mapping fixed brain samples with extracellular labeling and optical microscopy reveals synaptic connections.

## Main

Illuminating the complex structure of brain tissue has been a major motivating force to advance imaging technologies. Optical super-resolution approaches visualize cells and molecules at nanoscopic scales, increasing resolution beyond the diffraction limit of a few hundred nanometers by increasing instrument resolution^1–4^ or distances between features^5–8^. Super-resolution microscopy has generated insights into synaptic organization^9–11^, the neuronal cytoskeleton^12^, cellular structure–function relationships^13^ and tissue organization^14^. However, analysis has been limited to specific molecular targets or sparse subsets of labeled cells, lacking information about their context within the tissue. Electron microscopy (EM) provides comprehensive structural contrast and exquisite spatial resolution, but three-dimensional (3D) tissue reconstruction is technically challenging, laborious and difficult to complement with molecular information. Optical technologies visualizing the tissue’s architecture and providing contextual meaning to molecules and cellular structures at high resolution would provide major opportunities for discovery.

Extracellular labeling delineates all cells in a tissue in an unbiased fashion. It has been applied to guide patch-clamp experiments^15^ and visualize extracellular space (ECS)^16,17^ in living brain tissue and for EM connectomics^18^. Reading out freely diffusing, extracellularly applied fluorophores with stimulated emission depletion (STED) microscopy^1,19,20^ in living brain tissue by super-resolution shadow imaging^17,21–23^ casts super-resolved shadows of all cells. Such labeling reveals the tissue’s cellular architecture in a comprehensive manner down to nanoscopic scale. STED provides direct, ‘all-optical’ super-resolution with a light pattern confining fluorescence to sub-diffraction volumes. We recently showed that extracellular labeling integrated with a 3D super-resolution/machine learning technology enables dense, nanoscale reconstruction of living brain tissue^24^. However, although live imaging uniquely accesses dynamics, it is constrained in super-resolution modality, molecular labeling options, addressable tissue volumes and sample type. In fixed tissues, feature-rich representations of cells and tissues have been achieved, using fluorescent^25–29^ or Raman^30^ contrast for protein density or other molecule classes in expansion microscopy (ExM). However, none of these has been amenable to in silico reconstruction of brain tissue architecture or subcellular morphology. There is, thus, an unmet need for an optical technology capable of visualizing and quantifying tissue organization from regional to single-synapse level.

In this study, we developed Comprehensive Analysis of Tissues across Scales (CATS), an integrated labeling, optical imaging and analysis platform to decode brain tissue architecture, subcellular morphologies and molecular arrangements within their structural context. We engineered CATS to visualize all cellular structures in fixed tissue by extracellular labeling in (super-resolution) fluorescence microscopy. Thereby, CATS removes live-imaging constraints and permits analysis from regional to nanoscopic scales in common brain tissue preparations. It capitalizes on the full technology base for labeling, optically homogenizing and 3D super-resolution imaging available for fixed tissues, building on STED and ExM. CATS quantitatively reveals tissue architecture, maps synaptic connectivity and allows 3D reconstruction of subcellular morphology, including single synapses, in a molecularly informed fashion. To demonstrate the power of this approach, we characterized key synapse types in the hippocampal circuitry. We also visualized the synaptic input and output structure of functionally characterized neurons and applied the technique to human clinical specimens.

## Results

### CATS unravels tissue architecture at super-resolved detail

**Fig. 1:**
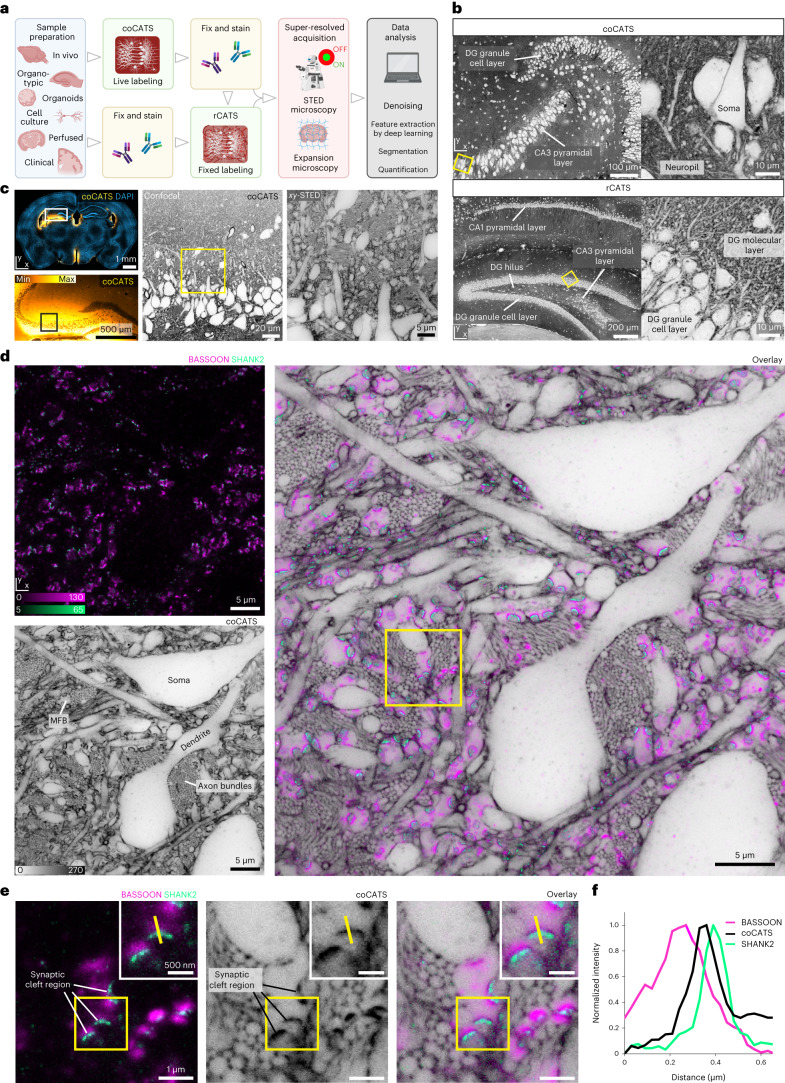
CATS. **a**, Platform for tissue analysis including live extracellular labeling (coCATS) or extracellular labeling in previously fixed tissue (rCATS), optional molecular staining, super-resolved acquisition and conventional/machine learning analysis. **b**, Top: coCATS labeling (STAR RED-NHS) in organotypic hippocampal slice, revealing gross architecture of the DG and CA3 region and zoomed view of boxed region (confocal). Data are representative of experiments in *n* = 10 slices. Raw data. Intensity lookup tables for CATS are inverted throughout—that is, black regions correspond to high labeling intensity, unless otherwise noted. Bottom: rCATS labeling (WGA-CF633) in perfusion-fixed adult mouse coronal section, showing hippocampus with zoomed view. Raw data. Data are representative of rCATS in *n* = 10 fixative perfused animals. **c**, Progressive zoom from hippocampal regional to cellular scale in CA3 stratum pyramidale and stratum lucidum. coCATS labeling by in vivo stereotactic injection (STAR RED-NHS) into the LV of adult mouse (left: lookup table not inverted; left bottom: gamma correction applied). Left, center: confocal; right: STED, lateral resolution increase (*xy*-STED). Raw data. **d**, Super-resolved tissue architecture of mouse CA3 stratum pyramidale/lucidum, after in vivo coCATS label (STAR RED-NHS) microinjection into LV. Left top: immunostaining of pre-synaptic BASSOON (magenta, confocal, AF488) and post-synaptic SHANK2 (turquoise, *xy*-STED, AF594). Left bottom: coCATS (*xy*-STED) of same region. Right: overlay placing synaptic markers into structural context, including MFBs. Raw data. Images are representative of coCATS with in vivo microinjection into the LV in *n* = 10 animals. **e**, Magnified view from **d** (boxed), focusing on an MFB with multiple synaptic sites, amidst bundles of thin mossy fiber axons. Inset: magnification of synaptic transmission site. High-intensity coCATS labeling pinpoints dense/protein-rich features between pre-synapses and post-synapses corresponding to pSCRs. **f**, Line profile as indicated in **e**, showing sandwich arrangement of BASSOON, high-intensity coCATS (pSCR) and SHANK2 signals.

We developed two extracellular labeling strategies (Fig. 1a). (1) ‘Compartment CATS’ (coCATS) applies covalently binding labels to the extracellular compartment in living tissue, with intact membrane boundaries constraining labeling to ECS and cell surfaces. (2) ‘Resident CATS’ (rCATS) labels extracellularly resident molecules, particularly polysaccharides, making CATS applicable to specimens where live labeling is not possible (Fig. 1b). Both approaches revealed the brain’s cellular architecture across scales—for example, in hippocampus (Fig. 1b,c)^31^.

For coCATS, we screened for labels providing high extracellular to intracellular contrast, high labeling density and compatibility with downstream super-resolution read-out (Supplementary Fig. 1). We focused on commercially available compounds for adoptability. We ensured cell impermeability via hydrophilic, anionic fluorophores or sulfo- or polyethylene glycol (PEG) groups. Chemistries targeting primary amines, including *N*-hydroxysuccinimide (NHS), tetrafluorophenyl and pentafluorophenyl esters, mediated covalent attachment to extracellular and cell surface molecules, particularly proteins. For read-out, we used either directly conjugated fluorophores or a small molecule reporter (biotin/fluorescent avidin).

**Extended Data Fig. 1:**
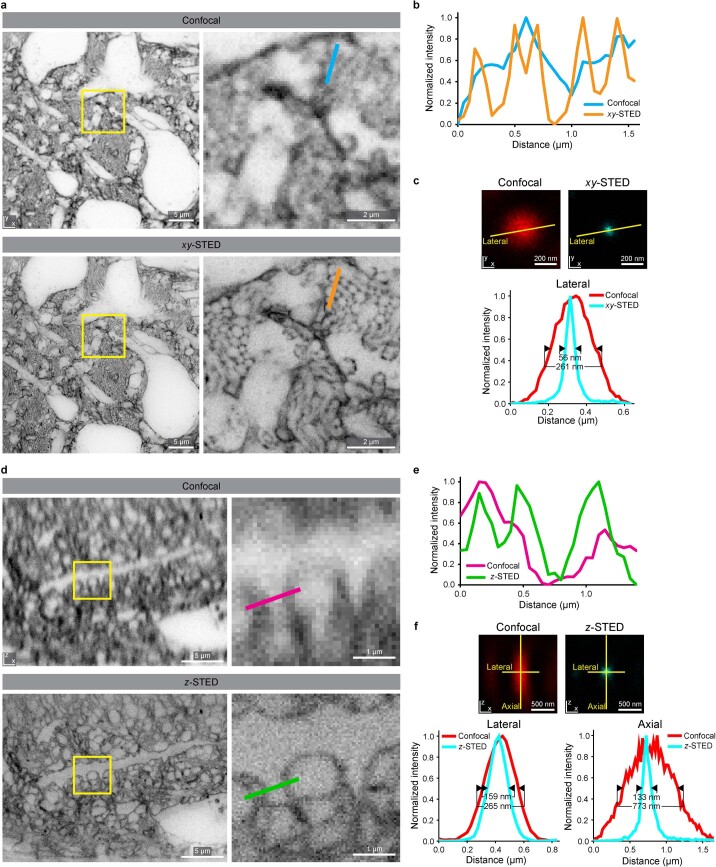
Improved visualization of tissue architecture with STED microscopy in coCATS. **a**, coCATS in adult mouse hippocampus (DG hilus) with labeling by in vivo microinjection into the lateral ventricle. Overviews and magnified views of the same region in confocal (*top*) and *xy*-STED mode (*bottom*) with lateral resolution increase. Tissue structure is visible more clearly in STED mode. For example, individual axons are discernible as fine rings only in STED mode. **b**, Line profile (width = 3 pixels) in a region of axon bundles as indicated by the lines in a, revealing modulation by individual axons in STED but not in confocal mode. **c**, Confocal and STED images of a single 40 nm Crimson bead with line profiles and full width at half maximum (FWHM). STED power was the same as in **a**. **d**, Single axial sections in the neuropil of an organotypic hippocampal slice with overviews and magnified views of the boxed region. Same region imaged in confocal (*top*) and *z*-STED mode (*bottom*). Resolution increase is stronger in the axial direction, yielding near-isotropic resolution. STED performance is high in the central ~10–15 µm of the axial range, for which correction of spherical aberrations was set by the objective’s correction collar. Decreasing STED performance above and below reflects the well-known sensitivity of the *z*-STED pattern to spherical aberration. **e**, Line profile (width = 3 pixels) as indicated in **c**. **f**, Confocal and STED images of a single 40 nm Crimson bead with corresponding line profiles. STED power was the same as in **d**. All data acquired with the same high-numerical aperture (NA = 1.35) silicone oil immersion objective. Comparison of confocal vs. STED performance in coCATS-labeled specimens was performed in *n* = 3 biological specimens. Imaging of fluorescent beads is representative of typical microscope performance and was acquired in one imaging session.

**Extended Data Fig. 2:**
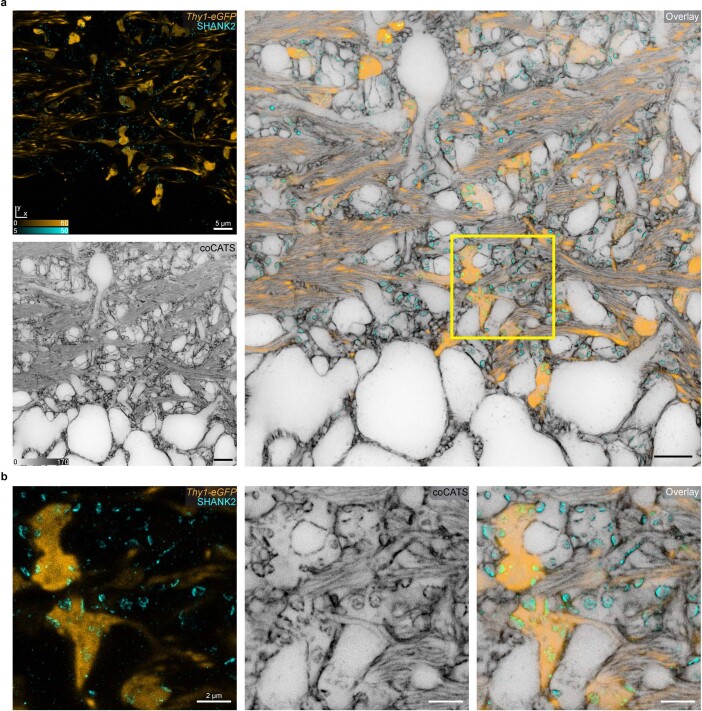
Information gain with CATS over sparse neuronal labeling. **a**, coCATS (gray, *xy*-STED) labeling by *in vivo* microinjection into the lateral ventricle of an adult mouse combined with a sparse genetic marker (*Thy1-eGFP*, yellow, confocal, immunostaining for eGFP) and a synaptic marker (immunostaining for SHANK2, *xy*-STED) shows the gain in information provided by CATS. **b**, Magnified view of the yellow box in **a**, showing mossy fiber boutons, some of which are positively labeled via eGFP expression. When using the sparse genetic marker alone, many synapses (indicated by presence of SHANK2) cannot be assigned within the tissue’s structure. CATS, in contrast, reveals not only the positively labeled, but all cellular structures. Displayed data are from a single dataset representative of coCATS with in vivo microinjection into the lateral ventricle performed in *n* = 10 animals.

For decrypting near-natively preserved brain, we stereotactically injected an NHS derivative of a hydrophilic, far-red STED-fluorophore in vivo, followed by transcardial fixative perfusion. Injection into the lateral ventricle (LV) labeled areas adjacent to the ventricular system, distant from the lesioned injection site (Supplementary Fig. 2). We first focused on hippocampus, a region central to spatial navigation and memory with well-characterized fundamental circuitry. Mossy fibers originating from dentate gyrus (DG) granule cells convey excitatory input to pyramidal neurons (PNs) in the CA3 stratum lucidum, forming key synapses in the hippocampal trisynaptic circuit. These are an established model for functional synapse characterization and contribute to higher-order computations^32,33^. STED imaging in the CA3 stratum pyramidale and lucidum revealed complex arrangements of cell bodies, dendrites, bundles of thin axons and synaptic terminals at high signal-to-noise ratio (Fig. 1d; see Methods and Supplementary Table 1 for labeling and imaging parameters). Diffraction-unlimited resolution, here ~60 nm laterally, was indispensable to resolve the densely arranged cellular structures (Extended Data Fig. 1). For example, STED resolved individual unmyelinated axons in mossy fiber bundles as circular structures when transversely optically sectioned. Complemented with immunolabeling for pre-synaptic BASSOON and for SHANK2, a scaffolding protein of excitatory post-synapses, CATS assigned molecularly defined synaptic sites to individual pre-synaptic boutons of mossy fibers and their post-synaptic counterparts, including complex PN spines^34^, termed ‘thorny excrescences’ (Fig. 1d–f). Such contextual structural meaning was missing with immunostainings alone or sparse labeling of cells by gold standard cytosolic fluorescent protein expression (Extended Data Fig. 2).

### Quantifying synapse structure

**Fig. 2:**
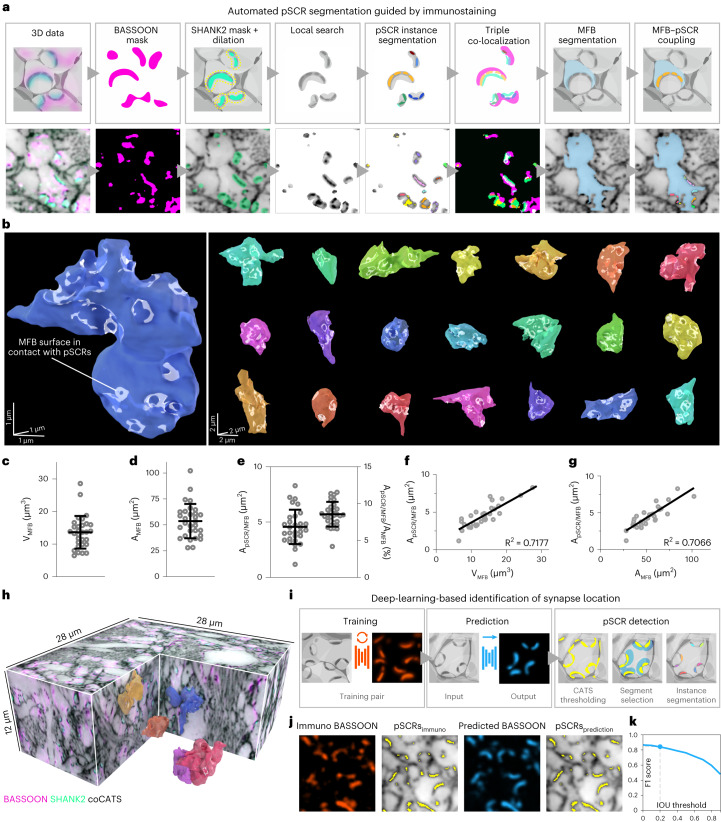
Synaptic connectivity and single-bouton properties. coCATS of hippocampal mossy fiber/CA3 PN synapses in adult mouse CA3 stratum lucidum with in vivo microinjection. **a**, Automated synapse detection guided by synaptic immunostaining. High-intensity 3D features in coCATS are segmented and classified as pSCRs if co-localized with pre-synaptic and post-synaptic markers and associated with manual volume segmentations of MFBs. Schematic (top) and single *xy* planes of volumetric data (bottom) including coCATS (gray, *z*-STED, STAR RED-NHS), BASSOON (magenta, confocal, AF488) and SHANK2 (turquoise, *z*-STED, AF594) (N2V, raw data: Supplementary Fig. 4). Imaging data are representative of in vivo microinjection into the LV in *n* = 10 animals. **b**, 3D renderings of 22 MFBs segmented from coCATS data at near-isotropic resolution (*z*-STED). MFB surface areas occupied by pSCRs (white) were automatically segmented and manually proofread. 3D scale bars refer to bouton center. N_MFB_ = 30 MFBs were reconstructed (10 from each of three imaging volumes recorded across two brain sections (one animal); additional renderings: Supplementary Fig. 7). **c**–**e**, MFB volume (V_MFB_) (**c**), surface area (A_MFB_) (**d**), absolute area (A_pSCR/MFB_) and relative area occupied by pSCRs on individual MFBs (A_pSCR/MFB_/A_MFB_) (**e**) (mean ± s.d., n_MFB_ = 30). Data points: individual MFBs. **f**,**g**, A_pSCR/MFB_ as function of bouton volume (**f**) and surface area (**g**) with linear regression (n_MFB_ = 30). **h**, One of the imaging volumes used for MFB characterization (N2V, raw data: Supplementary Fig. 4) with coCATS (gray, *z*-STED), BASSOON (magenta, confocal) and SHANK2 (turquoise, *z*-STED), including manually segmented MFBs and automatically detected pSCRs. **i**, Deep learning pSCR identification with training on paired structural (coCATS) and molecular (BASSOON immunostaining) super-resolved data. Prediction of synaptic marker location in unseen datasets is based on structural data alone. pSCRs are segmented similarly as in **a** but using predicted BASSOON instead of immunostainings. **j**, Immunostained (orange, *z*-STED) and predicted BASSOON distribution (blue) in a dataset not included in the training. Corresponding pSCRs (yellow) segmented from coCATS data (gray, *z*-STED, N2V), guided by immunostained (pSCRs_immuno_) or predicted BASSOON (pSCRs_prediction_). **k**, Similarity between pSCRs_immuno_ and pSCRs_prediction_ quantified by F1 score (range: 0–1, combining precision and recall; Methods) as a function of IOU threshold. No manual proofreading was applied in **j** and **k**. Training was performed on *n* = 13 imaging volumes recorded across four brain sections from *n* = 3 animals and testing on *n* = 1 dataset.

**Extended Data Fig. 3:**
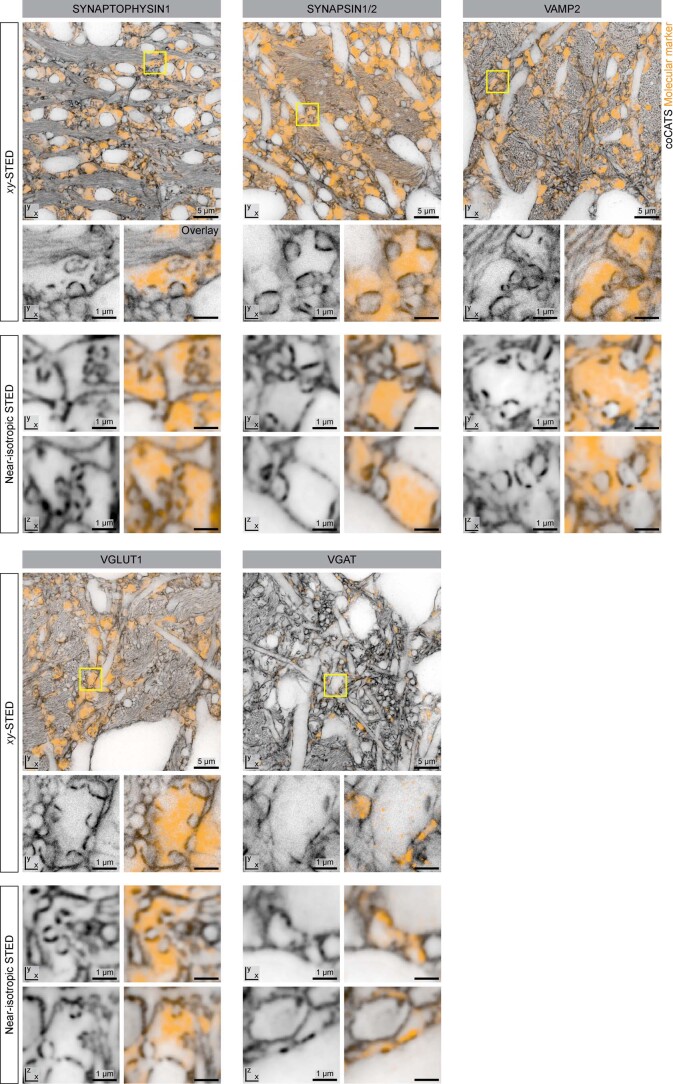
coCATS labeling pattern in relation to synaptic vesicle associated proteins. STED imaging in the CA3 stratum lucidum after in vivo microinjection into adult mouse brain and transcardial fixative perfusion. CoCATs labeling (gray) was performed with STAR RED-NHS and immunolabeling (orange) of SYNAPTOPHYSIN1, SYNAPSIN1/2, VAMP2 (vesicle-associated membrane protein 2), VGLUT1 (vesicular glutamate transporter, excitatory synapses) and VGAT (vesicular GABA transporter, inhibitory synapses) was performed with AF594. High-intensity coCATS features at putative synaptic cleft regions (pSCRs) are present both at excitatory and inhibitory synapses. Overview images for *xy*-STED with zoomed views (raw data) and high magnification views both in lateral and axial directions at near isotropic resolution. STED imaging at near isotropic resolution was performed with 80% of STED power with π-tophat phase modulation (*z*-STED pattern) and 20% of power 4π-helically phase modulated^24^ or with *z*-STED alone. N2V was applied to both channels independently. In some cases, 3D-histogram matching (Fiji/ImageJ Bleach Correction) was applied in the immunolabeling channel to compensate for limited antibody penetration. For detailed parameters see Supplementary Table 1. Tests for pSCR location relative to synaptic markers in Extended Data Figs. 3 and 4 were performed for a total of 10 markers across 17 brain slices from *n* = 6 animals.

**Extended Data Fig. 4:**
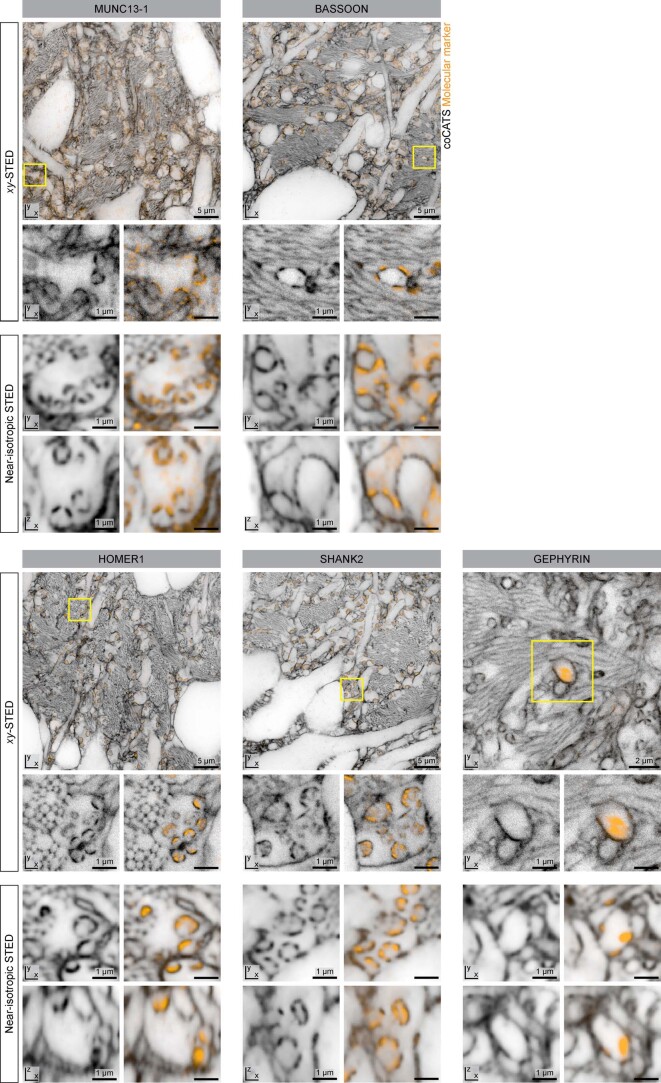
coCATS labeling pattern in relation to presynaptic and postsynaptic proteins. STED imaging in the CA3 stratum lucidum after in vivo microinjection into adult mouse brain and transcardial fixative perfusion. CoCATs labeling (gray) was performed with STAR RED-NHS and immunolabeling (orange) of the presynaptic active zone proteins MUNC13-1 and BASSOON, as well as the postsynaptic scaffolding proteins HOMER1 and SHANK2 present at excitatory synapses and GEPHYRIN present at inhibitory synapses. High-intensity coCATS features at pSCRs are present both at excitatory and inhibitory synapses. Overview images for *xy*-STED with zoomed views (raw data) and high magnification views both in lateral and axial directions at near isotropic resolution. STED imaging at near isotropic resolution was performed with 80% of STED power with π-tophat phase modulation (*z*-STED pattern) and 20% of power 4π-helically phase modulated^24^ or with z-STED alone. N2V was applied to both channels independently. In some cases, 3D-histogram matching (Fiji/ImageJ Bleach Correction) was applied in the immunolabeling channel to compensate for limited antibody penetration. For detailed parameters see Supplementary Table 1. Tests for pSCR location relative to synaptic markers in Extended Data Figs. 3 and 4 were performed for a total of 10 markers across 17 brain slices from *n* = 6 animals.

When inspecting combined structural/molecular data, we discovered that coCATS consistently produced high-intensity features sandwiched between pre-synapses and post-synapses. These correspond to putatively primary amine/protein-rich extracellular regions at apparent synaptic transmission sites, likely reflecting high protein density at synaptic clefts^35^ (Fig. 1e,f). We clarified their relationship with synaptic molecules in excitatory and inhibitory synapses, including vesicle markers SYNAPTOPHYSIN1 and SYNAPSIN1/2; vesicle-associated membrane protein 2; vesicular glutamate transporter; vesicular GABA transporter; pre-synaptic active zone proteins MUNC13–1; BASSOON; post-synaptic scaffolding proteins HOMER1, SHANK2 and GEPHYRIN; and sparsely labeled mossy fiber boutons (MFBs) (Extended Data Figs. 2–4). We found their location consistent with synaptic clefts, prompting us to designate them ‘putative synaptic cleft regions’ (pSCRs) and develop an automated pipeline for mapping them (Fig. 2a). After enhancing volumetric datasets with deep learning denoising (Noise2Void^36^ (N2V); Supplementary Figs. 3 and 4), we used super-resolved SHANK2 immunostaining as guide to excitatory synapses and performed locally confined thresholding to isolate high-intensity coCATS features. We classified these as pSCRs when adjacent to BASSOON (confocal) and SHANK2 (STED). This also eliminated false-positive identifications from unavoidable immunostaining background (Supplementary Fig. 5). Finally, we performed instance segmentation of pSCRs, applied manual proofreading based on CATS and immunolabeling and contextualized them by association with manual MFB volume segmentations. Automated analysis substantially reduced processing time compared to manual pSCR segmentation.

We reconstructed individual boutons with their synaptic transmission topology. Reconstruction is limited by the least-resolved direction—that is, along the optical (*z*) axis. We, therefore, applied a light pattern for near-isotropic STED resolution^1^ (*z*-STED, ~160-nm lateral and ~130-nm axial resolution; Extended Data Fig. 1 and Supplementary Fig. 6) and recorded three volumes in CA3 stratum lucidum (~30 × 30 × 12 µm^3^, two brain slices and one animal). We selected 10 prominent MFBs from each, manually segmented them from coCATS and quantified key geometrical parameters and pSCRs (Fig. 2b–h, Supplementary Videos 1 and 2 and Supplementary Fig. 7). Boutons varied in size and shape, with mean volume ${\bar{{\text{V}}}}_{{\rm{MFB}}}=\,13.6\pm 5.0\,{\upmu {\rm{m}}}^{3}$ (±s.d.) (Fig. 2c) and mean surface area ${\bar{{\text{A}}}}_{{\rm{MFB}}}=53.5\pm 16.6\,{\upmu {\rm{m}}}^{2}$ (Fig. 2d), consistent with EM results from adult mouse^37^ (${\bar{{\text{V}}}}_{{\rm{MFB}}}\,=\,13.5\,{\upmu {\rm{m}}}^{3}$,$\,{\bar{{\text{A}}}}_{{\rm{MFB}}}=\,66.5{\upmu {\rm{m}}}^{2}$). Mean surface area was smaller, as we did not include filopodia, which are at the limit of the resolution employed here. pSCRs were similarly diverse, often forming fenestrated structures (Fig. 2b). To identify MFB regions occupied by putative active zones, we related pSCRs to MFB segmentations. The total area of individual boutons occupied by pSCRs (A_pSCR/MFB_) had a mean of $\,{\bar{{\text{A}}}}_{{\rm{pSCR}}/{\rm{MFB}}}=4.6\pm 1.6{\upmu {\rm{m}}}^{2}$ (Fig. 2e). The fraction of MFB surface occupied by pSCRs (A_pSCR/MFB_/A_MFB_) at individual bouton level displayed smaller spread, hinting toward correlation between MFB size and extent of synaptic release sites. Indeed, when plotting A_pSCR/MFB_ as a function of MFB volume (Fig. 2f) (Pearson correlation coefficient *r* = 0.844, 95% confidence interval (CI): 0.694–0.923, two-tailed *P* value: *P* < 0.0001, R^2^ = 0.72, *n* = 30 MFBs) or surface area (Fig. 2g) (*r* = 0.841, CI: 0.689–0.922, two-tailed *P* value: *P* < 0.0001, R^2^ = 0.71, *n* = 30 MFBs), we found strong correlation, indicating that larger MFBs have more extensive synaptic contacts. This agrees with previous studies showing a linear relationship between MFB volume and active zone extent in organotypic slice cultures and in vivo^38^. The fraction of MFB surface area occupied by pSCRs (8.6 ± 1.7%) was consistent with previous quantifications of area occupied by active zones in serial-sectioning EM in adult rat (9.7%) on a smaller number of MFBs^39^. pSCR number was variable between boutons (3–28, mean 13.03 ± 5.93), similar as in EM data from adult rat^39^, and also correlated with bouton size (Supplementary Fig. 7b,c). These data demonstrate that CATS can identify synaptic transmission sites and deliver quantitative biological data at single-synapse level, consistent with EM reconstructions^37,39,40^ but including molecular information, at high throughput (imaging time for three-channel measurement per volume: ~1.5 h).

### Deep-learning-based synapse prediction

With the prominence of pSCRs, we hypothesized that coCATS may reveal synapse location based purely on local tissue structure. We trained a convolutional neural network with U-net architecture^41^ for image translation (Fig. 2i, Supplementary Note 1 and Supplementary Fig. 8). We trained the network with immunostainings as molecular ground truth and near-isotropically super-resolved coCATS data, using the resulting model to predict molecule location in unseen datasets. A model trained on coCATS and super-resolved BASSOON, present at excitatory and inhibitory synapses, was capable of guiding pSCR segmentation in MFBs, replacing immunostainings in our pSCR segmentation pipeline. This is remarkable, as thresholding alone, neglecting local context, was insufficient to identify pSCRs among dense CATS features. For validation, we correlated predicted with immunolabeled BASSOON in a dataset not included in the training (Supplementary Fig. 8a; Pearson correlation, *r* = 0.818). In addition to voxel-based correlation, we evaluated automated pSCR segmentation guided by immunostaining versus segmentation guided by predicted BASSOON and found high similarity (F1 = 0.84 at intersection over union (IOU) threshold 0.2; Fig. 2j,k, Supplementary Note 1 and Supplementary Fig. 8a,b). Denoising with N2V barely affected prediction outcome (Supplementary Fig. 8c,d). Predictions improved with super-resolved compared to confocal molecular signals as training input (Supplementary Fig. 8e,f). We furthermore benchmarked fully automated pSCR segmentations guided either by immunolabeling or predictions against manually generated ‘ground truth’ (Supplementary Fig. 8g–i). Both automated approaches detected a high fraction of synapses also without human intervention (F1 = 0.82 and 0.71 at IOU threshold 0.2 for immunolabeling and prediction-guided segmentations, respectively). These data demonstrate that deep-learning-based analysis within the CATS framework can reveal synaptic transmission sites, leveraging local context and structural labeling of pSCRs.

### Synaptic inputs of functionally characterized neurons

**Fig. 3:**
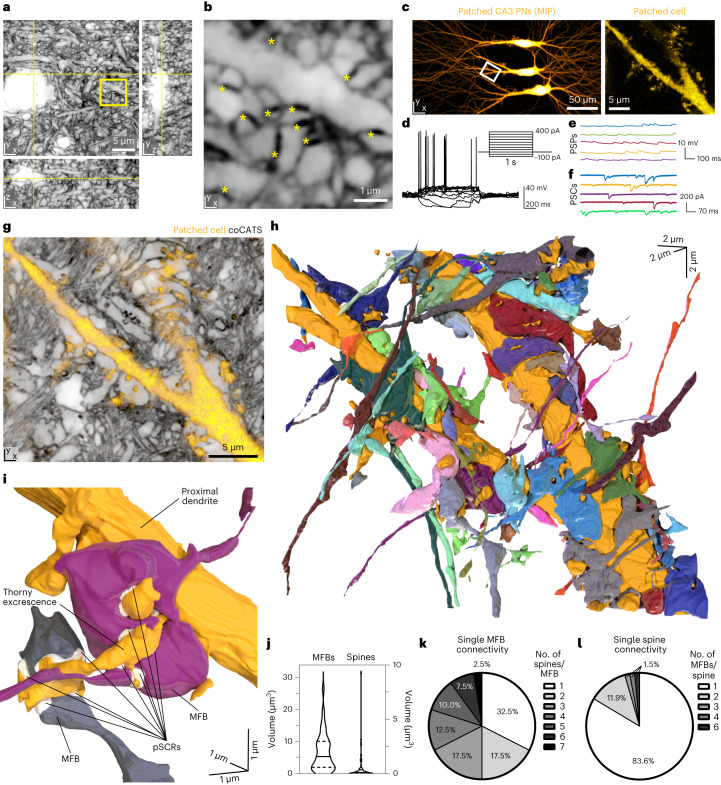
Reconstruction of CA3 PN local input field with coCATS. **a**, Orthogonal views of a coCATS imaging volume recorded with *z*-STED at near-isotropic resolution in neuropil of an organotypic hippocampal brain slice (N2V, raw data: Supplementary Fig. 4). Yellow lines indicate position of displayed planes. Label: ATTO643-NHS. **b**, Magnified view of the boxed region in **a**. Asterisks: pSCRs. Imaging data are representative of coCATS in *n* = 10 organotypic slices. **c**, Left: CA3 PNs in an organotypic hippocampal slice whole-cell patch-clamp recorded and filled with fluorescent dye (Lucifer yellow). Right: magnified view of a piece of proximal dendrite in the boxed region. MIP, maximum intensity projection. **d**, Action potential response of the middle PN elicited by current injection (inset). **e**,**f**, Spontaneous post-synaptic potentials (PSPs) and post-synaptic currents (PSCs) recorded from middle PN. **g**, coCATS (gray, *z*-STED, STAR RED-NHS, N2V, single *z*-section of volumetric dataset) overlaid with the intracellular label (yellow, confocal) of the middle PN provides super-resolved information on its local microenvironment. **h**, 3D rendering of the same proximal dendrite (gold) and 57 structures synaptically connected to it, reconstructed from the volumetric coCATS data. Connectivity was inferred by the presence of pSCRs between the positively labeled dendrite and the respective adjacent structures. **i**, 3D rendering of two MFBs (violet and gray) forming complex connections with one thorny excrescence of the proximal dendrite. pSCRs are indicated in white (identified by deep learning model from Fig. 2j,k). **j**, Violin plots with median (line) and quartiles (dashed lines) of the volumes of MFBs (n_MFB_ = 40) contacting the recorded PN and its spines (n_spine_ = 68). **k**,**l**, Quantification of connectivity pattern of individual MFBs and PN spines for that dendrite. Data in **c**–**g** are representative of coCATS labeling in combination with functional recordings and dye filling of various cell types in *n* = 6 organotypic slices. 3D reconstruction as in **h** and **i** was performed for *n* = 1 specimen, and analysis in **j**–**l** comprised n_spine_ = 68 spine structures and n_MFB_ = 40 MFBs. Three additional MFBs were only partially contained within the imaging volume and, thus, not included in quantifications.

To integrate structural with functional information, we performed coCATS in organotypic hippocampal slices (Fig. 3a,b and Supplementary Video 3) after whole-cell patch-clamp recordings. CATS revealed pSCRs and provided context to electrophysiologically characterized neurons, filled with fluorophores during recording for later identification (Fig. 3c–e and Supplementary Fig. 9). Recordings during and after coCATS labeling showed that activity (induced action potential generation) continued (Supplementary Fig. 10), demonstrating that neurons were functional at the time of fixation.

**Extended Data Fig. 5:**
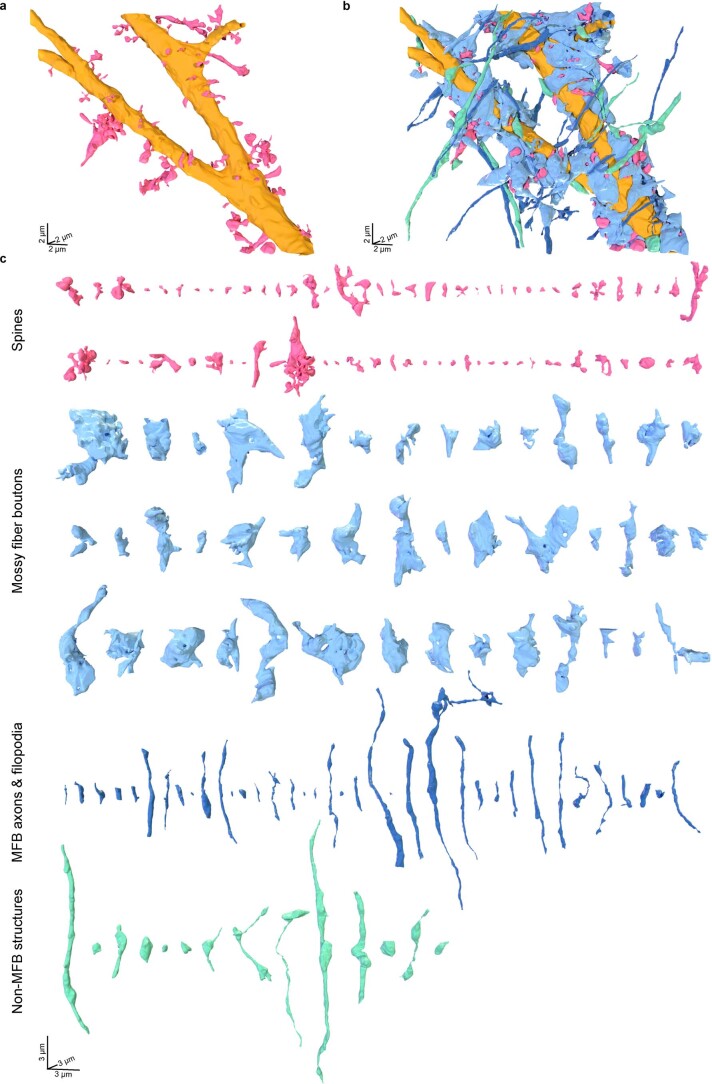
Structural characterization of the local input field in a CA3 pyramidal neuron proximal dendrite. **a**, 3D-rendering of the CA3 pyramidal neuron proximal dendrite in Fig. 3g based on coCATS data. The dendritic shaft is colored in gold, spines (n_spines_ = 68) are labeled in magenta. **b**, 3D-rendering of the same dendrite as in **a**, with associated cellular structures color-coded by identity, as inferred from morphology: MFBs (n_MFBs_ = 43, light blue), axons and filopodia of MFBs (n_axons/filopodia_ = 38, dark blue), structures in synaptic contact with the main dendrite, not identifiable as MFB-related structures (n_non-MFB_ = 14, turquoise). **c**, 3D-renderings of all structures reconstructed in **b**. Reconstruction was performed on *n* = 1 dataset.

CATS visualized neurons with surrounding structures, revealing key information missing with sparse positive cellular labeling alone (Fig. 3c,g). We mapped the synaptic inputs of a proximal dendrite in an electrophysiologically characterized CA3 PN at near-isotropic STED resolution (Fig. 3g,h and Extended Data Fig. 5). Proximity of pre-synaptic and post-synaptic structures is unreliable for predicting connectivity^42^. However, with deep-learning-based pSCR segmentation and manual validation, coCATS allowed us to identify structures connected by chemical synapses (Supplementary Fig. 11). We reconstructed 57 (43 MFB and 14 non-MFB) structures connected to a dendrite stretch of the recorded cell to clarify the 3D arrangement of MFBs and complex spines (Fig. 3h,i and Supplementary Video 4). Reconstructed MFBs displayed a wide range of sizes, with smaller mean volume and larger spread (Fig. 3j; 6.85 ± 5.95 µm^3^, n_MFB_ = 40 completely contained in imaging volume) than the manually selected MFBs in adult brain in Fig. 2, potentially reflecting an earlier developmental stage^38^ in the ~20-d in vitro cultures. The 68 reconstructed spines included complex structures—that is, quintessential thorny excrescences. However, the size distribution was skewed toward small spines contacting MFBs (Fig. 3j). We next evaluated connectivity of individual MFBs (Fig. 3k). Only ~1/3 of MFBs connected to single spines, whereas synaptic contact with multiple (up to seven) spines was more common. Conversely, especially small spines mostly contacted single MFBs, but some (16.4%), mostly elaborate spines, were contacted by more than one (up to six) MFBs (Fig. 3i,l). This highlights the complex organization of the mossy fiber circuitry, with signal integration occurring even at individual spine level. More broadly, it demonstrates the power of CATS to provide quantitative data on structural and functional connectivity.

### Synaptic output structure across regions

**Fig. 4:**
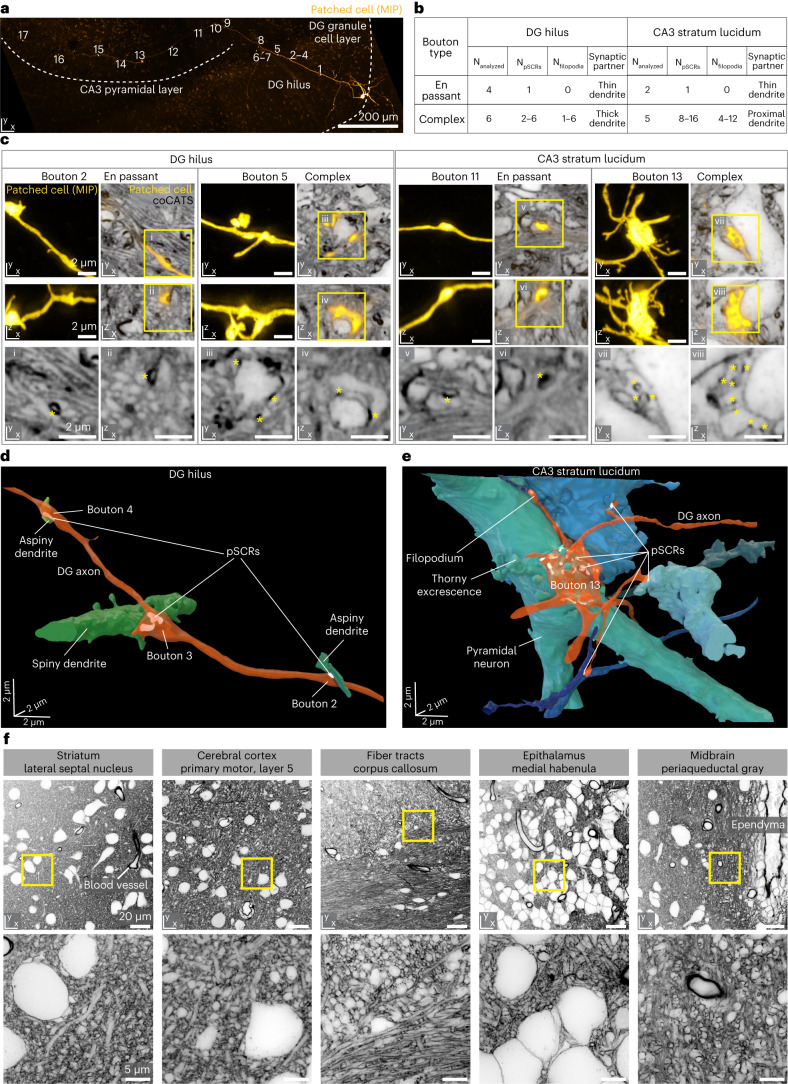
Tissue architecture and single-cell output structure at individual synapse level across brain regions. **a**, MIP of a whole-cell patch-clamped and biocytin-filled DG granule cell in organotypic hippocampal slice (confocal, visualized with AF594-streptavidin). Seventeen conspicuous boutons are marked along the main axon’s trajectory, projecting as mossy fiber from the DG granule cell layer through the hilus to the CA3 stratum lucidum. **b**, Characteristics of analyzed synaptic boutons. **c**, Single *xy* and *xz* planes of four example super-resolved volumes comprising specific synapses as marked in **a**, with coCATS (gray, *z*-STED, STAR RED-NHS, N2V) revealing local microenvironment of the positively labeled mossy fiber (yellow, *z*-STED, N2V) (raw data: Supplementary Fig. 4). Bottom: magnified views of the coCATS channel with asterisks indicating pSCRs used to identify synaptic partners. pSCRs were segmented with the same model as in Fig. 2j,k, followed by manual proofreading. **d**,**e**, 3D renderings of two axon stretches with boutons, pSCRs and synaptically connected structures in DG hilus and CA3 stratum lucidum. coCATS labeling in combination with functional recordings is representative of experiments in *n* = 6 organotypic slices. Following the axon trajectory with 3D reconstruction was done for *n* = 1 sample, with bouton characteristics extracted from a total of N_analyzed_ = 17 boutons imaged across multiple volumes along the axon. **f**, Architecture of various regions in near-natively preserved brain revealed by coCATS with in vivo microinjection. Organization of cell bodies, dendrites, axons, synapses, ependyma around liquor spaces and blood vessels is visible. Top: confocal; bottom: *xy*-STED. Images represent raw data from *n* = 5 brain slices obtained from *n* = 2 independent biological specimens with in vivo microinjection into LV and primary motor cortex, respectively. They are representative of coCATS in vivo microinjection in *n* = 10 and *n* = 4 animals for LV and cortical microinjection, respectively.

**Extended Data Fig. 6:**
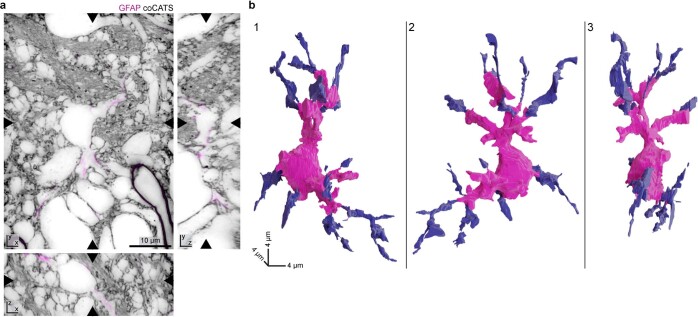
Astrocyte visualization with coCATS. **a**, Volumetric STED dataset in the mouse hippocampal CA3 stratum lucidum recorded at near-isotropic resolution after in vivo microinjection of coCATS label (gray, STAR RED-NHS) into the lateral ventricle and transcardial fixative perfusion. Glial fibrillary acidic protein (GFAP, magenta), an intermediate filament expressed in astrocytes, was immunolabeled (AF594) and imaged with near-isotropic STED. Data was denoised with N2V. Arrowheads at image edges indicate position of corresponding orthogonal sections. **b**, 3D-renderings of manual segmentations of an astrocyte in the imaging volume in **a**, viewed from three different perspectives. Manual segmentation of coCATS data was first performed blinded (magenta) to GFAP. Then the GFAP data was provided to the segmenter, allowing to assign additional structures (violet) in the CATS channel, in particular fine protrusions, to that cell. Note that segmentation was still based on the coCATS channel, as intermediate filaments do not fully trace out cell shape. coCATS in vivo microinjection into the lateral ventricle was performed in *n* = 10 biological specimens. Astrocyte 3D-reconstruction was performed once.

We next characterized the synaptic output field of a DG granule cell in an organotypic hippocampal slice. We performed coCATS after electrophysiological recording and biocytin filling and followed the main axon from the DG granule cell layer through the hilus to the CA3 stratum lucidum (Fig. 4a). We applied volumetric, near-isotropically resolving STED imaging around 17 conspicuous, mostly complex pre-synaptic boutons (Fig. 4b,c and Supplementary Fig. 12). Although the axon trajectory and bouton structure could be determined from the super-resolved, positive single-cell label, CATS was required to reveal structural context and identify post-synaptic partners via pSCRs, segmented by the deep learning pipeline with manual validation (Fig. 4c and Supplementary Figs. 12 and 13). We analyzed complex MFBs and smaller en passant boutons with identified pSCRs. En passant boutons displayed a single pSCR onto thin dendrites and lacked filopodia. In contrast, large boutons featured multiple pSCRs and filopodia in the hilus (4.0 ± 2.0 filopodia per bouton) and CA3 stratum lucidum (8.5 ± 3.4 filopodia per bouton). They formed complex synapses with hilar mossy cells and CA3 PNs, respectively, identifiable from their morphology and context in CATS. We reconstructed synaptic units in hilus (Fig. 4d and Supplementary Video 5) and CA3 stratum lucidum (Fig. 4e and Supplementary Video 6), showing differential complexity between en passant (boutons 2 and 4) and complex boutons (bouton 13), with the latter bouton contacting nine post-synaptic structures (Fig. 4e). Connections included engulfment of thorny excrescences by the main bouton and contacts via filopodial extensions. We also observed pSCRs at filopodia, which are thought to predominantly contact inhibitory interneurons^43^. Tracing axons from CATS data was not possible at the chosen resolution (Supplementary Fig. 14). We, therefore, used the positive label to follow the axon across regions in Fig. 4, whereas coCATS visualized tissue architecture. More generally, pairing CATS with molecular information can molecularly identify cell types or assign structures to individual cells, such as the sheet-like protrusions of an astrocyte (Extended Data Fig. 6 and Supplementary Video 7).

### Differential tissue architecture

**Extended Data Fig. 7:**
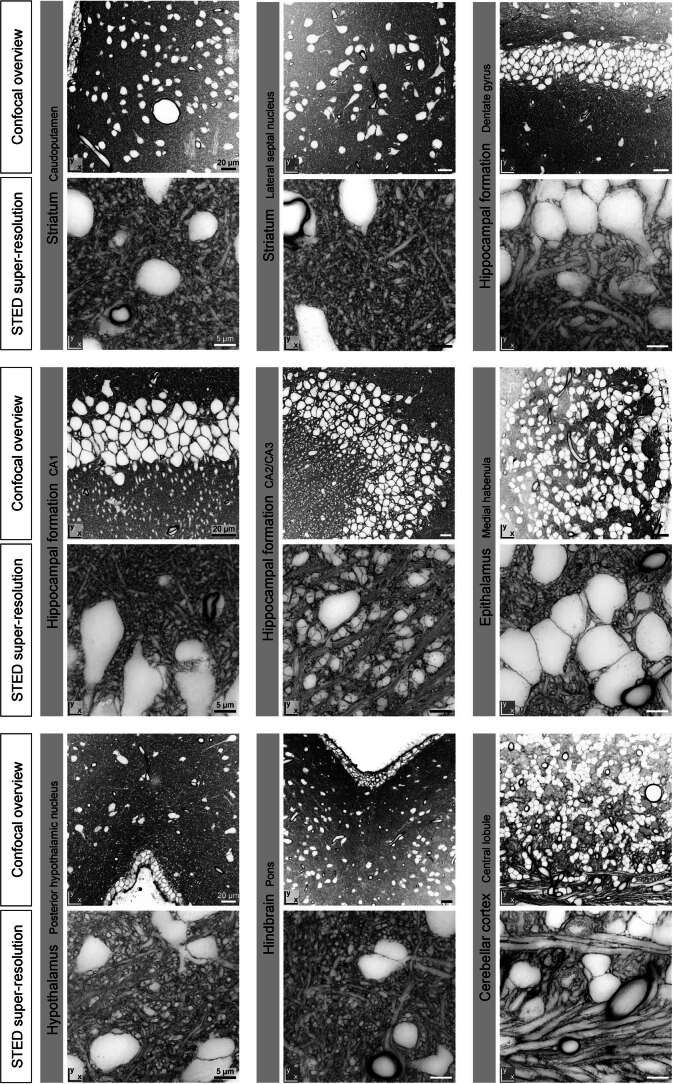
Tissue organization in diverse brain regions. coCATS in various brain regions after in vivo microinjection into the lateral ventricle or cortex. (*Top*) Confocal overview images. Scale bars: 20 µm. (*Bottom*) Higher magnification STED images with lateral resolution increase (*xy*-STED) from the same regions. Raw data. Scale bars: 5 µm. Imaging data is representative and was acquired across eight different brain sections from *n* = 3 individual biological specimens. coCATS labeling of various brain regions was achieved by in vivo microinjection into the lateral ventricle or cortex, which was performed in *n* = 10 and *n* = 4 biological specimens, respectively.

Seeking to reveal tissue architecture beyond hippocampus, we returned to in vivo coCATS labeling. Microinjection into LV or cortex (Supplementary Fig. 15) visualized the diversity of cellular architecture in cortex, hippocampus, striatum, corpus callosum, epithalamus, hypothalamus, hindbrain and cerebellum (Fig. 4f and Extended Data Fig. 7). Tissue was intact beyond ~200 µm of damage around the injection site (Supplementary Fig. 15). STED disclosed rich structural detail of neuronal and glial processes, synapses, axon bundles, blood vessels and ependyma. For some myelinated axons, the inner demarcation of the myelin sheath was visible (Supplementary Fig. 16), albeit at lower contrast than with rCATS below.

### CATS in previously fixed tissue

**Fig. 5:**
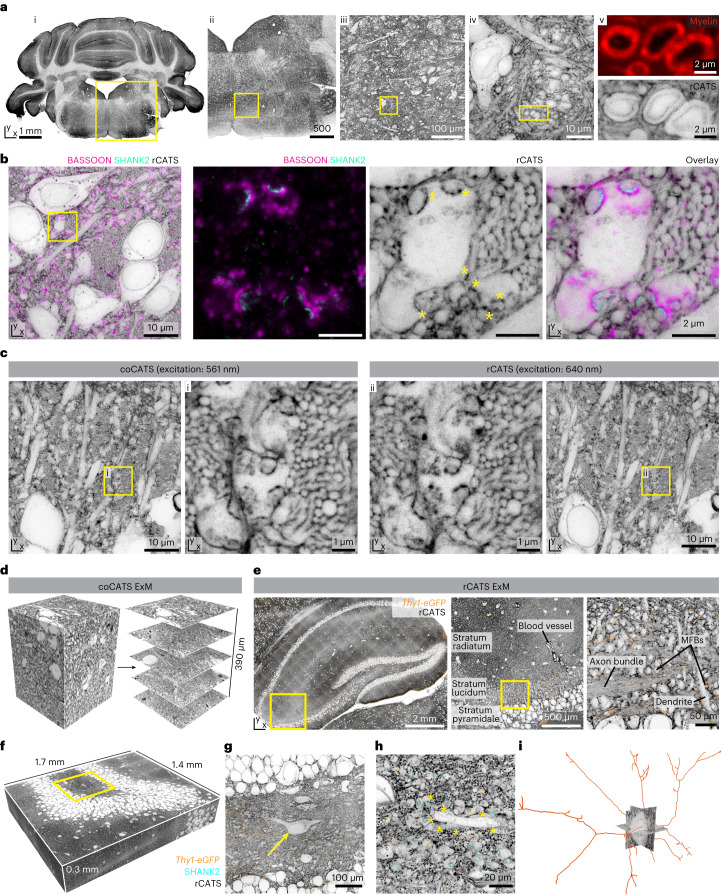
CATS in previously fixed tissue and CATS with ExM. **a**, rCATS (WGA-CF633) in coronal section of cerebellar cortex and hindbrain from fixative-perfused mouse. Overview (left) and progressive zoom-ins in the medulla as indicated. i–iii: confocal; iv: *xy*-STED; v, top: myelin sheaths (FluoroMyelin, confocal), bottom: rCATS (*xy*-STED). **b**, rCATS (gray, WGA-CF633, *xy*-STED) in hippocampal DG hilus of fixative-perfused mouse with SHANK2 (turquoise, *xy*-STED) and BASSOON (magenta, confocal) immunolabeling. Zoomed views: MFBs surrounded by mossy fibers. Asterisks: dense labeling at pSCRs. Data in **a**,**b** are representative of rCATS in n = 10 perfusion-fixed specimens. **c**, Combined coCATS (*xy*-STED, excitation 640 nm) and rCATS (*xy*-STED, excitation 561 nm) in CA3 by LV microinjection of AF594-NHS, perfusion fixation and rCATS labeling with WGA-CF633. Magnified views: mossy fibers and complex synapses. rCATS/coCATS co-labeling was performed in seven brain sections across *n* = 3 animals with various fluorophore combinations. **d**, Organotypic hippocampal slice with coCATS (NHS-PEG_12_-biotin), ~4-fold expanded via MAP^6^. Confocal imaging volume (left, N2V) and single planes at increasing depth (right). The ~400-µm axial range corresponds to ~100 µm in original tissue. Data are representative of experiments in *n* = 3 organotypic slices. **e**, Hippocampal section from perfusion-fixed *Thy1-eGFP* adult mouse (eGFP visualized by immunostaining, orange), with rCATS (WGA-biotin) and ~4-fold expansion by proExM^8^ and zoomed views in CA3 (confocal, raw). Scale bars refer to size after expansion throughout. **f**, 3D representation of DG crest volume (303 × 371 × 70 µm^3^ original size) in perfusion-fixed *Thy1-eGFP* mouse imaged with spinning-disc confocal microscopy after 4.5-fold expansion, with rCATS (gray, WGA-biotin, N2V) and immunostaining for SHANK2 (cyan, N2V) and eGFP (orange, N2V). **g**, Magnified view of single *xy* plane as indicated by yellow box. Arrow: hilar mossy cell. **h**, Different plane at higher magnification. The central dendrite belongs to the mossy cell in **g**, lined by MFBs with SHANK2 at synaptic sites. Yellow asterisks: subset of MFBs in contact with the dendrite. **i**, Skeletonization of major branches of the hilar mossy cell in **g** and **h** from rCATS data. Whole-section rCATS with proExM was performed in six brain slices across *n* = 4 animals and skeletonization in *n* = 1 dataset.

**Extended Data Fig. 8:**
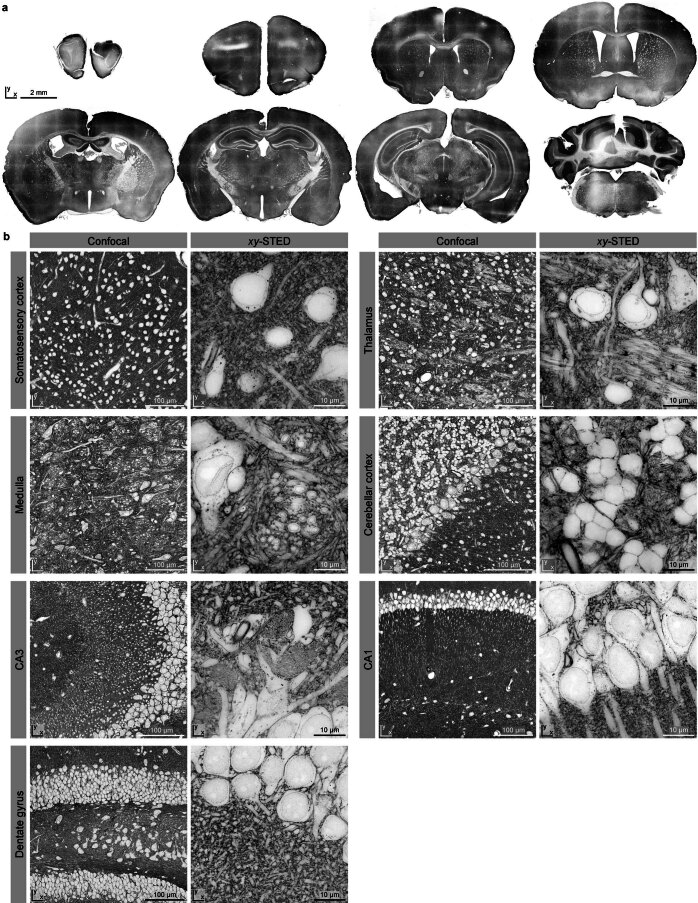
rCATS across the adult mouse brain. **a**, Serial coronal sections of a brain from a perfusion fixed adult mouse, revealing organ architecture with rCATS (WGA-CF633) in confocal overview imaging. Light areas typically correspond to regions rich in cell bodies or fiber tracts. Data are acquired across serial sections from the same biological specimen. **b**, rCATS in various example regions at two different magnifications, using WGA-AF594 or WGA-CF633 (see Supplementary Table 1 for detailed parameters). (*Left*) confocal, (*right*) *xy*-STED revealing differential tissue organization in different brain regions. The overview in the somatosensory cortex covers the superficial molecular layer (layer I, upper right corner) and layers II/III below. In the thalamus, imaging was performed in the ventral posterior nucleus, with the ad-axonal line in many of the fibers suggestive of myelination. In the medulla, bundles of thick nerve fibers are prominent. The ad-axonal line points to a myelination of these fibers. Imaging of the cerebellar cortex was performed in the vermal region, with the granular, Purkinje, and molecular layers visible in the overview. STED imaging was performed in the granular layer. In the hippocampal CA3 region overview, the different layers are conspicuous (left to right: stratum radiatum, stratum lucidum, stratum pyramidale). In the overview of the DG (*left*), the hilar region in the center is bordered by the granule cell layer above and below. The STED image (*right*) shows the transition between granule cell layer (top) and molecular layer (bottom) of the lower DG blade. In the CA1 region, the following layers can be discerned in the overview from top to bottom: stratum oriens, stratum pyramidale, stratum radiatum, stratum lacunosum moleculare. The STED image is from the transition between stratum pyramidale and stratum radiatum. All images are raw data. Imaging data in **b** were acquired across brain slices from *n* = 6 biological specimens.

**Extended Data Fig. 9:**
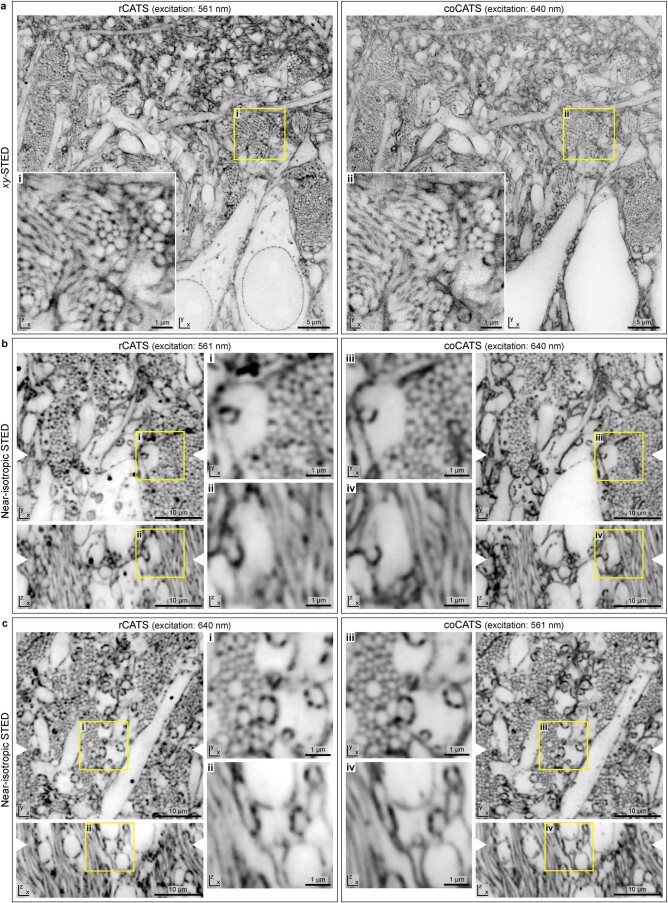
Direct comparison of coCATS and rCATS in the same specimen. **a**, STED imaging (*xy*-STED, raw data) in the CA3 region, with stratum radiatum at the top and stratum lucidum in the center in an adult mouse brain after injection of STAR RED-NHS into the lateral ventricle, perfusion fixation and rCATS labeling with WGA-AF594. Note that here, color channels are swapped relative to Fig. 5c, to account for differences in stimulated emission cross section between the fluorophores. The magnified region mainly contains mossy fibers. Both labeling paradigms yield detailed visualization of tissue architecture. **b**, Lateral and axial sections with near-isotropic STED imaging in a similarly prepared brain in the CA3 stratum lucidum using WGA-AF594 for rCATS and STAR RED-NHS for coCATS. STED imaging was performed at near-isotropic resolution with 80% of STED power with π-tophat phase modulation (*z*-STED pattern) and 20% of power 4π-helically phase modulated^24^. N2V was applied to both channels independently. **c**, Similar measurement but with color channels swapped, using WGA-CF633 for rCATS and AF594-NHS for coCATS. coCATS-rCATS co-labeling was performed in 7 brain sections total from *n* = 3 animals with different fluorophore combinations.

For several preparations, live labeling is not possible. We, therefore, screened binders to ECS-resident molecules widely and homogeneously distributed in mouse brain (rCATS). Different polysaccharide-binding proteins showed distinct labeling patterns, reflecting ECS molecular diversity (Supplementary Fig. 17). We chose wheat germ agglutinin (WGA) for rCATS. It binds to *N*-acetyl-d-glucosamine and sialic acid and has been used to outline blood vessels or cell bodies^44,45^. Labeling fixed mouse brain with fluorescent WGA revealed hippocampal architecture (Fig. 1b). In fact, rCATS in a serially sectioned mouse brain showed high-quality labeling across the organ (Extended Data Fig. 8). Zooming in and super-resolving various regions, including medulla, cortex, hippocampus, thalamus and cerebellum, revealed histoarchitecture at nanoscale detail (Fig. 5a and Extended Data Fig. 8). Carbohydrate-rich features, including nuclear pores, were distinguishable with rCATS. Myelinated axons, validated by myelin staining, typically showed an ad-axonal line in STED mode, allowing identification with rCATS (Fig. 5a and Supplementary Fig. 18). We furthermore confirmed that rCATS was compatible with immunostaining (Fig. 5b). Next, we compared rCATS and coCATS in the same specimen—that is, applying rCATS after in vivo microinjection of coCATS label and fixative perfusion. Both visualized mossy fibers, boutons and cell bodies in CA3 stratum lucidum (Fig. 5c and Extended Data Fig. 9), indicating that high-density labeling can also be obtained with rCATS. Slightly lower resolution is expected in the shorter wavelength (561 nm) than in the far-red (640 nm) excitation channel due to lower stimulated emission cross-section. However, channels can be assigned following experimental needs (Extended Data Fig. 9). Despite different labeling mechanisms, we observed dense features similar to pSCRs at synaptic transmission sites also in rCATS and put them into structural context of MFBs (Fig. 5b,c and Supplementary Fig. 19). However, in direct comparison, coCATS staining appeared somewhat more homogeneous and with higher signal-to-noise ratio, such that we restricted pSCR analysis to coCATS. We also characterized rCATS performance and depth penetration for different fixation and permeabilization conditions (Supplementary Note 2 and Supplementary Fig. 20).

### Large-scale tissue analysis with ExM

ExM involves hydrogel embedding, disruption of mechanical cohesiveness and isotropic swelling, while conserving spatial arrangements^5^, providing super-resolution with diffraction-limited read-out. It reduces autofluorescence and homogenizes refractive index, mitigating aberrations and scattering, thus clearing the tissue. This facilitates acquisition of extended, super-resolved volumes. We, therefore, sought to combine CATS’ capability to decode tissue architecture with the strengths of ExM. Expansion requires a label that is retained in the hydrogel and is minimally affected by the radical chemistry during polymerization and heat/chemical denaturation. Biotin fulfills this, such that we screened for biotin-containing coCATS labels (Supplementary Fig. 1). We found that an additional chemical group was required for sufficient extracellular-to-intracellular contrast and chose PEG_12_. We live-labeled organotypic hippocampal slices with NHS-PEG_12_-biotin and expanded ~4-fold with the magnified analysis of proteomes (MAP)^6^ (Fig. 5d and Supplementary Fig. 21) or protein-retention ExM (proExM)^8^ (Supplementary Fig. 21) approaches, using heat/chemical denaturation and enzymatic digestion to disrupt cohesiveness, respectively. We applied fluorophore-conjugated streptavidin for readout after expansion. This provided signal amplification and flexibility with downstream processing. We recorded confocal stacks of ~400-µm axial range, obtaining super-resolved context over a 100-µm range at native tissue scale.

Combining rCATS with expansion, we realized that WGA features few lysines for hydrogel anchoring, resulting in poor retention upon expansion (Supplementary Fig. 22). We developed a signal retention strategy (Supplementary Fig. 22), transferring information from biotinylated WGA to acrylamide-modified streptavidin co-polymerizing with the gel and read out with biotin-coupled fluorophores. Large-scale imaging of expanded samples with spinning-disc confocal microscopy allowed high-resolution visualization of tissue architecture (Fig. 5e,f and Supplementary Fig. 23). To illustrate the rich information contained in this data, we imaged a 1.4 × 1.7 × 0.32-mm^3^ (post-expansion; expansion factor 4.5; 303 × 371 × 70 µm^3^ pre-expansion; ~0.5 TB) volume of the DG crest and hilus, wherein rCATS provided structural context to sparse *Thy1-eGFP* neurons (where eGFP means enhanced green fluorescent protein) and excitatory synapses labeled for SHANK2 (Fig. 5f–h). We skeletonized major dendritic arborizations of an unlabeled example neuron. This cell, identified as a mossy cell by its morphology and connectivity with MFBs, can be studied in its 3D context, demonstrating the utility of rCATS for unbiased imaging and analysis of any neuronal population (Fig. 5g–i).

### CATS in human nervous tissue

**Fig. 6:**
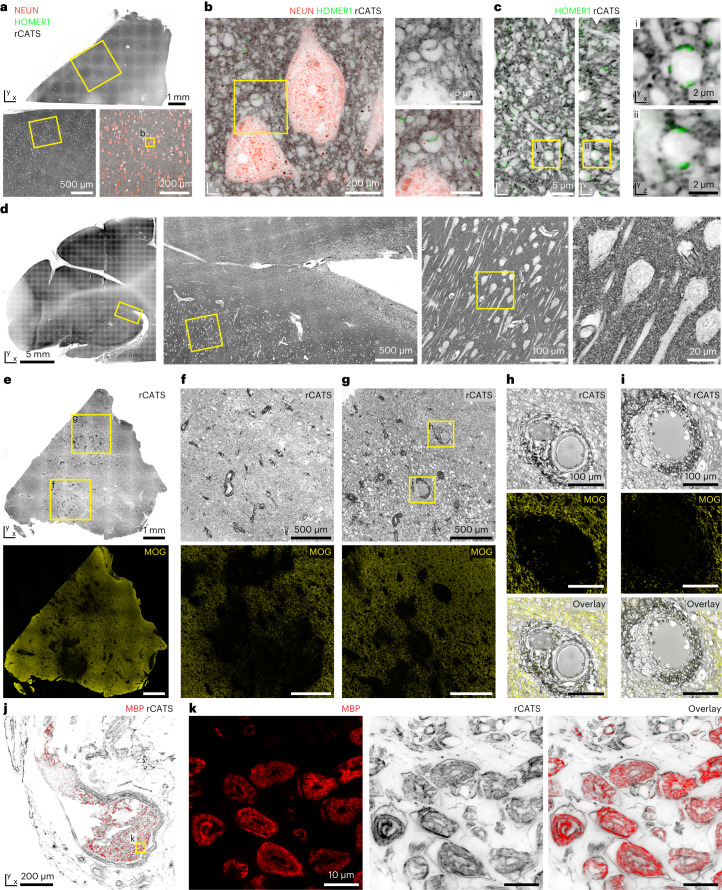
Tissue architecture in human nervous tissue. **a**, rCATS (gray, WGA-CF633) in temporo-medial cortex from a 35-year-old male patient undergoing epilepsy surgery, with staining for mature neurons (NEUN, orange, AF594) and excitatory synapses (HOMER1, green, AF488). Confocal overview (top) with progressive zooms (bottom). **b**, STED image (*xy*-STED) with zoom onto synapses with rCATS (top) and molecular information (bottom, confocal). **c**, Orthogonal views of imaging volume with rCATS (near-isotropic STED, gray) and HOMER1 (confocal, green). Arrowheads: positions of orthogonal views. N2V was applied to channels independently. rCATS was performed on surgery explants from *n* = 8 patients, and the best-preserved specimens were selected for display here and in Supplementary Fig. 24. **d**, rCATS (confocal, WGA-CF633) in archival human FFPE autopsy specimen of a 35-year-old female patient without brain pathology (postmortem interval >12 h, storage time 16 years). Progressive zooms in hippocampus. rCATS was performed in five slices from autopsy specimens of *n* = 2 individuals. **e**–**i**, rCATS in a patient with MOGAD. Archival FFPE tissue specimen from brain biopsy for histopathological diagnostics in a 53-year-old female patient. **e**, rCATS (top, WGA-CF633, confocal) and immunostaining for MOG (bottom, AF488, confocal). Absence of MOG indicates demyelination. **f**,**g**, Magnified confocal views. White voids indicate tissue edema. A subset of infiltrating immune cells features conspicuous rCATS labeling, likely reflecting intracellular accumulation of carbohydrate-containing myelin degradation products. **h**,**i**, rCATS (top), MOG immunolabeling (middle) and overlay (bottom, confocal) of blood vessels indicated in **g**. Perivascular inflammatory infiltrate displaces nervous tissue from vessel walls. Additional markers: Supplementary Fig. 26. Data are representative of *n* = 3 technical replicates from *n* = 1 patient with MOGAD. **j**,**k**, Peripheral human nerve (*N. suralis*) of a 44-year-old female patient, visualized by rCATS in FFPE nerve biopsy. **j**, Overview with rCATS (gray, WGA-CF633, confocal) and immunolabeling for MBP (red, AF488), with nerve and connective tissue sheath embedded in fatty/connective tissue. **k**, Higher magnification view, with rCATS (*xy*-STED) and MBP (confocal). Axons are enwrapped by myelin, with neurofilament H immunolabeling confirming location of central axon (Supplementary Fig. 27). rCATS data are representative of *n* = 2 technical replicates in *n* = 1 patient.

Conventional stainings for human clinical specimens, such as hematoxylin and eosin (H&E), coarsely represent tissue architecture. To test whether CATS is adoptable to human samples, we obtained fixed cortical tissue from a patient undergoing surgery for epilepsy treatment. Also in human samples, rCATS revealed contextual information at confocal and STED resolution in cortex (Fig. 6a,b) and hippocampus (Supplementary Fig. 24). Concomitant immunolabeling for mature neurons (NEUN), excitatory post-synapses (HOMER1) and neuronal processes (microtubule-associated protein 2) placed molecular information into tissue context. rCATS also allowed detailed, yet straightforward, assessment of tissue preservation, the major quality determinant for microanatomical studies in clinical material. In contrast, immunostainings alone made it challenging to determine effects of tissue degradation, as target molecules were sparsely distributed (Supplementary Fig. 24). We next tested whether rCATS was applicable to formalin-fixed and paraffin-embedded (FFPE) human postmortem brain. We obtained FFPE tissue stored for 16 years from a diagnostic pathology archive and found rCATS to reveal cellular architecture (Fig. 6d and Supplementary Fig. 25), despite a postmortem interval of more than 12 h before fixation. We then visualized tissue structure in human brain pathology, choosing an FFPE brain biopsy obtained for histopathological diagnosis of myelin oligodendrocyte glycoprotein (MOG) antibody-associated disease (MOGAD), a demyelinating inflammatory disease associated with auto-antibodies against the myelin component MOG. rCATS detailed the inflammatory cellular infiltrate, tissue edema and destruction of histoarchitecture, with MOG immunolabeling highlighting demyelinated regions (Fig. 6e–i and Supplementary Fig. 26). Labeling for immune cell antigens ionized calcium-binding adapter molecule 1 and CD68 highlighted microglia and macrophages (Supplementary Fig. 26). Testing applicability to the peripheral nervous system, we applied rCATS to an FFPE sural nerve biopsy from a patient suspected with peripheral neuropathy and validated locations of axon cylinders and myelin with immunolabelings for neurofilament H and myelin basic protein (MBP), respectively. rCATS broadly visualized tissue architecture, including individual axons, the nerve sheath, connective tissue and vasculature. Individual myelinated axons were spaced from each other, consistent with edema and moderate axonal polyneuropathy (Fig. 6j,k and Supplementary Fig. 27). rCATS is, thus, a valuable resource for studying tissue structure and single-cell morphology in clinical specimens of healthy and diseased individuals.

**Extended Data Fig. 10:**
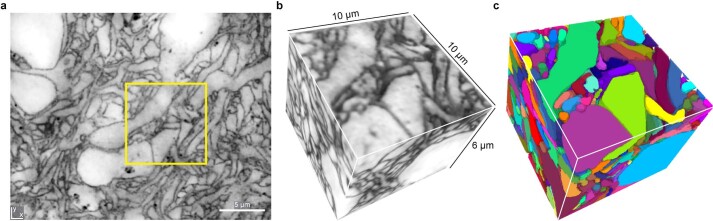
CoCATS in human cerebral organoid. **a**, Single plane of super-resolved volume (*z*-STED, N2V, adaptive histogram equalization). **b**, Subvolume of the same dataset, as indicated in **a**. **c**, Dense tissue reconstruction with coCATS via manual segmentation of the volume in **b**. Imaging data are representative of coCATS labeling in *n* = 5 human cerebral organoids cultured at 3 different time points. Dense manual reconstruction was performed in one dataset.

Finally, we sought to demonstrate applicability of CATS to human cerebral organoids, emerging as an experimentally tractable human model for brain development and disease^46^. We asked whether CATS could densely reconstruct the cellular constituents of an organoid volume. We chose coCATS, which is less dependent on deposition of extracellular matrix. Using STED at near-isotropic resolution allowed dense cellular segmentation (Extended Data Fig. 10 and Supplementary Video 8). The organoid showed lower complexity than the other sample types analyzed. However, this proof-of-principle experiment paves the way for large-scale dense reconstruction of complex tissue samples with light microscopy.

## Discussion

In this study, we developed CATS, a platform to map brain tissue architecture across spatial scales with light microscopy. CATS labeling is performed either in the living (coCATS) or the fixed (rCATS) state. Further downstream labeling and imaging ensue after fixation. This opens broad possibilities for molecular interrogation and analysis of diverse specimens and extended volumes. Contrary to sparse cellular labeling, CATS demarcates ECS and cell surfaces, thus displaying the tissue’s constituents in an unbiased fashion. This is possible in dense brain tissue at diffraction-limited resolution or comparatively moderate resolution increase over the diffraction limit, as CATS creates a boundary between cells, such that the structural imaging channel remains free from intracellular complexity.

CATS is applicable to diverse brain regions and a wide variety of commonly used tissue preparations, including native rodent brain, mouse organotypic slices, human cerebral organoids and previously fixed mouse and human brain, including surgery and archival FFPE specimens for histopathological diagnostics. We chose coCATS with 3D STED for nanoscale reconstructions and detection of pSCRs because of its indiscriminate, high-density labeling. However, in optimally preserved (perfusion-fixed) mouse brains, rCATS produced similar results. In coCATS, labeling depends on diffusion in living brain, extending hundreds of micrometers beyond the injection site. In rCATS, labeling depends on extracellular carbohydrate distribution and label diffusion into fixed tissue. To capitalize on CATS’ capability to visualize tissue architecture, care is warranted to preserve tissue structure. We opted for mild permeabilization allowing labeling a few tens of micrometers from the sample surface, well matched with the depth penetration of 3D STED microscopy. For large-scale imaging, we developed ExM strategies for both coCATS and rCATS. This increased resolution with conventional (high-speed) microscopes, but we observed somewhat reduced labeling density, presumably due to incomplete label anchoring. An obvious improvement will be optimization of signal retention^47^, whereas light-sheet microscopy can enhance imaging speed^14^. Our goal was to provide readily adoptable strategies for visualizing brain tissue architecture. Tracing the finest neurites, including tortuous axons, and their synaptic connections—that is, connectomic reconstruction—may ultimately be possible with CATS or similar approaches but will require increasing optical resolution or expansion factors^28,29,48–51^. CATS is a technologically straightforward approach for 3D tissue analysis in applications where EM resolution is not essential and directly bridges spatial scales (mm–nm), avoiding complex correlation between imaging modalities.

We used hippocampal circuitry as first application target. Quantifications of MFB geometry and connectivity were consistent with benchmark EM data^37,39,40^, whereas CATS easily incorporated molecular information and reduced requirements in time, personnel and equipment over classical serial-sectioning EM. For example, imaging the three volumes for reconstructing 30 MFBs in Fig. 2 required ~4-h hands-on sample preparation and 3 × 1.5-h imaging time.

Despite different labeling mechanisms, we observed pSCR features both in coCATS and rCATS, using them in coCATS to infer synaptic partners. Mere high-intensity features are not predictive of synaptic connections. However, combining high-intensity CATS features, 3D super-resolved context and immunolabeling or deep learning prediction of synaptic markers allowed us to decide, in most cases, whether a synaptic transmission site was present, distinguishing, for example, from immunolabeling background or other high-intensity CATS features. This differs from synapse detection in EM, where structural visualization at higher resolution, including synaptic vesicles, is used, with F1 scores in automated synapse detection varying according to approach and testing set size^52^ (Supplementary Note 1). We designed a deep learning image translation pipeline for predicting molecule location from CATS data, training on immunolabelings rather than human annotations, which are labor intensive to generate. The deep learning approach recalled ~82% of synapses identified by immunolabeling (IOU threshold 0.2; Supplementary Fig. 8). When using pSCRs to infer or quantify synaptic connections, we applied manual proofreading. Both of our automated approaches reduced human annotation time. Although we observed pSCRs in excitatory and inhibitory synapses, we were predominantly interested in the hippocampal circuitry, with excitatory mossy fiber synapses representing a large fraction of our training data. Generalization to arbitrary synapse types or purely automated synapse detection can potentially be achieved by a more diverse training base and refined prediction approaches.

Throughput of 3D reconstruction was limited by manual cell-shape segmentation and will benefit from deep learning adopted from EM connectomics^53–55^, as employed in super-resolution reconstruction of living brain tissue^24^, making large-scale studies of tissue architecture feasible. We expect CATS to seamlessly integrate with complementary technologies, including calcium imaging or viral circuit tracing^56,57^, similar to the structural/functional characterization demonstrated here with patch-clamp recordings.

High throughput, easy adoptability and seamless pairing of structural data with molecular and functional information puts CATS in an excellent position to phenotype brain tissue in an unbiased way in rodent and patient-derived human specimens and clarify structure–function relationships and disease correlates.

## Methods

### Samples

#### Animals

Animal procedures were performed in accordance with national law (BGBLA 114 and Directive 522), European Directive 2010/63/EU and institutional guidelines for animal experimentation and were approved by the Austrian Federal Ministry for Education, Science and Research (authorizations BMBWF-V/Sb: 2020-0.363.126 and 2021-0.547.215). Experiments performed on cultured organotypic brain slices involved organ extraction after killing the animal, which does not require ethics authorization.

Animals were housed in groups of 3–4 animals under controlled laboratory conditions (12-h light/dark cycle with lights on at 7:00, 21 ± 1 °C, 55 ± 10% humidity) with food (pellets, 10 mm) and autoclaved water ad libitum. Animals were housed in commercially available individually ventilated cages made from polysulfone with a solid cage floor, dust-free bedding (woodchips) and nesting material.

For all experiments, male and female mice were used interchangeably to demonstrate the technology. Adult (3–5 months) C57BL/6J and STOCK Tg(Thy1-eGFP)MJrs/J (*Thy1-eGFP*, Jackson Laboratory, 007788, hemizygous) mice were used for in vivo microinjection and/or perfusion experiments as indicated. Five- to 7-day-old C57BL/6J, *Thy1-eGFP* or *PSD95-HaloTag* mice^58,59^ (homozygous or heterozygous) (courtesy of Seth Grant, University of Edinburgh) were used to prepare organotypic hippocampal slice cultures. Available PSD95-HaloTag-positive slices were used in screening experiments in Supplementary Fig. 1 to reduce overall animal number, but HaloTag was not used for labeling.

#### Human surgery and archival specimens

Human hippocampal and cortical samples were obtained from patients undergoing temporal lobe surgery for epilepsy treatment after obtaining informed consent. Procedures were approved by the Ethics Committee of the Medical University of Vienna (authorizations EK 1188/2019 and EK 2271/2021). Patients did not receive compensation. Human archival autopsy and biopsy material from FFPE brain and nerve tissue was identified at the Neurobiobank of the Division of Neuropathology and Neurochemistry, Department of Neurology, Medical University of Vienna. Research use of these samples was approved by the Ethics Committee of the Medical University of Vienna, EK 1123/2015 and EK 1636/2019, which provides a common broad consent (biobank consent) according to the Austrian Research Organization Act 2018, §2d, para 3 (biomaterial can be used within an entire research area as long as the patient has not withdrawn).

#### Human cerebral organoids

Research involving human H9 embryonic stem cells (line WAe009, https://hpscreg.eu/cell-line/WAe009-A) and cerebral organoids derived thereof was approved by the Ethics Committee at the Institute of Science and Technology Austria (ISTA Ethics Committee, approval date 9 June 2020).

### Experimental methods

Information on labeling probes and concentrations for each measurement is detailed in Supplementary Table 1. For details on reagents, including antibodies and solutions with abbreviations, see the subsection ‘Reagents’ in Supplementary Information.

#### Fixative perfusion

Adult mice were first anaesthetized with isoflurane (1–2% (v/v)) and then deeply anesthetized with ketamine (80–100 mg kg^−1^ of body weight) and xylazine (10 mg kg^−1^) intraperitoneally, combined with metamizol (200 mg kg^−1^) subcutaneously for analgesia. After checking for deep anesthesia by toe pinch, they were transcardially perfused with 10 ml of ice-cold 1× PBS, followed by 80 ml of ice-cold fixative solution (4% (w/v) paraformaldehyde (PFA) EM grade, 0.1 M PB, 0.1 M NaOH, pH 7.4) at a flow rate of 7–8 ml min^−1^. Brains were dissected and post-fixed in 5 ml of fixative solution overnight (ON) at 4 °C on an orbital shaker.

#### Tissue processing

Perfused mouse brains were washed 3× for 1 h each with 1× PBS on an orbital shaker at room temperature. Serial coronal sections of 50–100-µm thickness were prepared with a vibratome (Leica VT 1200 S). Sections were kept in 0.02% (w/v) NaN_3_ in 1× PBS at 4 °C for short-term storage (1–2 weeks) or in cryo-protectant solution (60% (v/v) glycerol in 0.1 M PB) at −20 °C for long-term storage.

#### Tissue culture

##### Organotypic hippocampal slice culture

Organotypic hippocampal slices were prepared according to the membrane interface method with slight modifications^60^. Five- to 7-day-old mice were decapitated with surgical scissors. The brain was dissected and placed in ice-cold 10 mM d-glucose in HBSS (−/−). Hippocampi, including entorhinal cortex, were dissected, and slices were obtained perpendicular to the longitudinal axis of the hippocampus at 350-µm thickness with a tissue chopper (McIllwain). Microporous cell culture inserts (pore size 0.4 µm, PICM0RG50, Millicell) were placed in culture dishes in 1 ml of culture medium (MEM supplemented with 15% (v/v) heat-inactivated HS; 2% (v/v) B-27; 25 mM HEPES; 3 mM GlutaMAX; 2.8 mM CaCl_2_; 1.8 mM MgSO_4_; 0.25 mM ascorbic acid; 6.5 g L^−1^ d-glucose) and equilibrated at 37 °C with 5% CO_2_. Sliced hippocampi were transferred into a new dish with ice-cold 10 mM d-glucose in HBSS (−/−). Slices were inspected with a microscope, and 6–7 slices per brain were transferred onto the cell culture inserts, 3–4 slices per insert. Excess HBSS was withdrawn with a filter paper. Slices were cultured at 37 °C with 5% CO_2_. Medium was exchanged 2× per week. Fresh medium was pH equilibrated and temperature equilibrated in the incubator for ≥30 min before medium change. Slices were typically used for experiments 14–30 d after culture start (days in vitro (DIV)). The sample in Fig. 5d was cultured as described previously^24^.

##### Human cerebral organoids

H9 human embryonic stem cells (https://hpscreg.eu/cell-line/WAe009-A) were obtained from a commercial provider (WA09, lot no.: WIC-WA09-RB-001, WiCell). Authentication was performed by the provider by short tandem repeat analysis, karyotype analysis (G-banding) and flow cytometry for embryonic stem cell markers. Human cerebral organoids were generated with a modified protocol from ref. ^61^ as described previously^62^. In brief, human embryonic stem cells were dissociated with Accutase and seeded in ultra-low-binding 96-well plates (Corning) containing mTeSR1 medium supplemented with 50 µM Y-27632. Cells were fed every 2 d, and supplements were removed from the media after 3 d of culture. After the cells aggregated to embryoid bodies, these were transferred into low-adhesion 24-well plates containing neural induction medium (50 ml of DMEM/F-12, 0.5 ml of N-2, 0.5 ml of GlutaMAX supplement, 0.5 ml of MEM-NEAA, 1 µg ml^−1^ heparin). Day 0 of cerebral organoid formation was defined at the start of neuroepithelial tissue formation. The organoids were embedded in Corning Matrigel matrix droplets. Growth medium was first exchanged to cerebral organoid medium without vitamin A (125 ml of DMEM/F-12, 125 ml of neurobasal, 1.25 ml of N-2, 5 ml of B-27 without vitamin A, 2.5 µg ml^−1^ insulin, 50 µM 2-mercaptoethanol, 2.5 ml of GlutaMAX, 1.25 ml of MEM-NEAA, 2.5 ml of PenStrep), followed by cerebral organoid medium with vitamin A (250 ml of DMEM/F-12, 250 ml of neurobasal, 2.5 ml of N-2, 10 ml of B-27, 2.5 µg ml^−1^ insulin, 50 µM 2-mercaptoethanol, 5 ml of GlutaMAX, 2.5 ml of MEM-NEAA, 500 µM ascorbic acid, 5 ml of PenStrep, 0.2% (w/v) NaHCO_3_) 4 d later. The organoids were placed on a horizontal shaker and fed 2× per week.

#### Electrophysiological recordings

Electrophysiological recordings were obtained from hippocampal organotypic slice cultures at 10–21 DIV in artificial cerebrospinal fluid (ACSF; 125 mM NaCl, 25 mM NaHCO_3_, 25 mM glucose, 2.5 mM KCl, 1.25 mM NaH_2_PO_4_, 2 mM CaCl_2_ and 1 mM MgCl_2_, with pH maintained at 7.3, equilibrated with carbogen (95% O_2_/5% CO_2_)) at ~22 °C. Micropipettes were pulled from thick-walled borosilicate glass (2-mm outer diameter, 1-mm inner diameter) and filled with intracellular solution (135 mM K-gluconate, 20 mM KCl, 0.1 mM EGTA, 2 mM MgCl_2_, 4 mM Na_2_ATP, 0.3 mM GTP, 10 mM HEPES), with 1 mg ml^−1^ Lucifer yellow or 0.2% (w/v) biocytin as required. Pipettes were positioned using up to four LN Mini 25 micromanipulators (Luigs & Neumann) under visual control on a modified Olympus BX51 microscope with a ×60 immersion objective (Olympus LUMPlan FI/IR, ×60, numerical aperture (NA) 0.90, working distance (WD) 2.05 mm). Up to four neurons were simultaneously recorded in whole-cell patch-clamp configuration, with signals acquired on Multiclamp 700B amplifiers (Molecular Devices), low-pass filtered at 6 kHz and digitized at 20 kHz with a Cambridge Electronic Design 1401 mkII AD/DA converter. Signals were acquired using Signal 6.0 software (CED). Action potential phenotypes were recorded on sequential current pulse injections (−100 pA to +400 pA) in current-clamp configuration. Neurons were identified based on morphological properties and spike frequency upon current injection. In current-clamp recordings, pipette capacitance was 70% compensated. Recordings were analyzed using Stimfit^63^ and MATLAB-based scripts.

#### Stainings

##### Immunolabeling

Samples were permeabilized with 0.2–0.5% (v/v) TX in 1× PBS ON at 4 °C with gentle agitation and washed 3× for 30 min each with 1× PBS, or by 4–5 freeze–thaw cycles (see below), unless otherwise noted. Brain slices were blocked with blocking solution (5% (w/v) BSA, 1% (v/v) NGS, 0.02% (w/v) NaN_3_ in 1× PBS) for 4 h at room temperature with gentle agitation. Samples were incubated with primary antibodies (ABs) in 5% (w/v) BSA in 1× PBS ON at 4 °C or room temperature with gentle agitation. They were washed 3× for 30 min each with 5% (w/v) BSA in 1× PBS at room temperature with gentle agitation. Secondary AB incubation was performed in 5% (w/v) BSA, 1% (v/v) NGS, 0.02% (w/v) NaN_3_ in 1× PBS either ON at 4 °C or at room temperature with gentle agitation. Samples were washed thoroughly with 1× PBS.

##### Other stainings

Positive labeling of single cells by dye filling. For confocal imaging after patch-clamp recording in organotypic hippocampal slices, cells were filled with 1 mg ml^−1^ Lucifer yellow during recording (Fig. 3 and Supplementary Fig. 9). For STED super-resolution read-out, cells were filled with 0.2% (w/v) biocytin during recording (Fig. 4 and Supplementary Fig. 12). After fixation, the slices were permeabilized with 0.2% (v/v) TX in 1× PBS for 7 h at 4 °C with gentle agitation. Slices were washed 3× for 30 min each with 1× PBS at room temperature with gentle agitation, followed by a 2-h blocking step in 5% (w/v) BSA and 1% (v/v) NGS in 1× PBS at room temperature with gentle agitation and incubation with 4 µg ml^−1^ Alexa Fluor 594-streptavidin in 1× PBS ON at 4 °C with gentle agitation. They were then washed 3× for 30 min each with 1× PBS at room temperature with gentle agitation.

Additional lectin stainings. For lectin stainings other than WGA, perfusion-fixed mouse brain sections were permeabilized with 0.5% (v/v) TX in 1× PBS ON at 4 °C with gentle agitation. Samples were washed 3× for 30 min each with 1× PBS at room temperature with gentle agitation.

For LEL labeling, the samples were incubated with 2.5 µg ml^−1^ LEL DyLight 594 in 1× PBS for 2 h at room temperature with gentle agitation. The samples were washed 3× for 30 min each with 1× PBS at room temperature with gentle agitation before imaging.

For biotin-conjugated lectins, samples were incubated with 5–8 µg ml^−1^ lectin in 1× PBS with 2 mM CaCl_2_ for 20 h at 4 °C with gentle agitation and washed 3× for 30 min each with 1× PBS at room temperature with gentle agitation, followed by incubation with 4 µg ml^−1^ Alexa Fluor 594-streptavidin for 2 h at room temperature or 4 °C ON on an orbital shaker. The samples were washed again 3× for 30 min each with 1× PBS.

Hyaluronic acid-binding protein. Adult mouse PFA perfusion-fixed coronal brain sections were washed 3× for 30 min each with 1× PBS at room temperature with gentle agitation and incubated with 10% (w/v) BSA and 0.2% (v/v) TX in 1× PBS ON at 4 °C on an orbital shaker. The samples were then incubated with 10 µg ml^−1^ HABP-biotin in 10% (w/v) BSA and 0.2% (v/v) TX in 1× PBS for 48 h at 4 °C with gentle agitation. The sections were washed 3× for 30 min each with 1× PBS at room temperature with gentle agitation.

FluoroMyelin staining. Perfusion-fixed coronal brain sections were washed 3× for 30 min each with 1× PBS at room temperature on an orbital shaker. Sections were permeabilized with 0.5% (v/v) TX in 1× PBS ON at 4 °C with gentle agitation and washed 3× for 30 min each with 1× PBS at room temperature with gentle agitation, followed by rCATS staining. The sections were then incubated with FluoroMyelin in 1× PBS (diluted according to the manufacturer’s recommendations) ON at room temperature with gentle agitation. The samples were washed 3× for 30 min each with 1× PBS on an orbital shaker.

Nuclear stain. Nuclei were stained with 0.5–1 µg ml^−1^ DAPI (1:5,000–10,000 dilution of a 5 mg ml^−1^ stock in ddH_2_O) for 15–30 min at room temperature with gentle agitation. DAPI incubation was performed in 1× PBS for all samples, except for expanded hydrogels, which were incubated in ddH_2_O. After the staining, samples were washed 2× for 15 min each with 1× PBS or ddH_2_O (expanded hydrogels). Nuclear stains were performed as the last step before imaging.

#### coCATS

##### Stereotactic surgery for in vivo microinjection of coCATS labeling compounds

Adult mice were first anesthetized with isoflurane (1–2%) and then deeply anesthetized with ketamine (80–100 mg kg^−1^ of body weight) and xylazine (10 mg kg^−1^) intraperitoneally, combined with metamizol (200 mg kg^−1^) subcutaneously for analgesia. The head was shaved; OleoVital was applied to the eyes; and the animals were head fixed in a stereotactic frame (David Kopf Instruments). Bregma and lambda were aligned to the same height. A small hole was drilled at the injection coordinate, and the injection pipette was lowered to the brain surface (used as vertical reference point) and advanced into the tissue. Using a microinjection pump (Nanoliter 2010, World Precision Instruments), highly concentrated coCATS labeling solution (20 mM amine-reactive compound in DMSO) was injected over 10 min using the following coordinates (measured from bregma):

- LV: 1.20–1.25 mm caudally, ±2–2.1 mm laterally and 2 mm vertically, for injections into the right or left LV. A total volume of 500 nl was injected at 50 nl min^−1^.
- Cortex: 0.71 mm caudally, 1.65 mm laterally and 1 mm vertically, for injections into the right primary motor cortex. A total volume of 100 nl was injected at 10 nl min^−1^.

After injection, the pipette was left for 5 min in situ to prevent backflow before slowly retracting it. Mice were placed on a heating pad during and after surgery until transcardial perfusion. The level of anesthesia was confirmed by toe pinch. If necessary, additional ketamine/xylazine was administered. The procedure was followed by transcardial perfusion 40–45 min after onset of dye delivery.

##### coCATS labeling of organotypic hippocampal slice cultures

Organotypic slices were used at 14–25 DIV for experiments. A piece of membrane including a slice was cut from the cell culture insert and immersed in carbogen-equilibrated, pre-warmed ACSF with HEPES (20 mM d-glucose, 4.8 mM KCl, 125 mM NaCl, 26 mM NaHCO_3_, 1.25 mM NaHPO_4_×H_2_O, 2 mM CaCl_2_, 1.3 mM MgCl_2_, 7.5 mM HEPES in ddH_2_O, pH 7.4). coCATS labeling compound was freshly prepared in carbogen-equilibrated ACSF with HEPES from a highly concentrated stock (typically 20–100 mM in DMSO). For direct fluorophore labeling, 40–50 µM STAR RED-NHS or 50 µM ATTO643-NHS (Fig. 4a,b) was used. For expansion experiments, 250 µM NHS-PEG_12_-biotin was used (Fig. 5d and Supplementary Fig. 21). The slice was immersed in coCATS labeling solution and incubated at 37 °C for 20–25 min (direct labeling with fluorophore) or 45 min (biotin labeling for expansion) with gentle agitation. The sample was washed 2× for 1 min with carbogen-equilibrated ACSF with HEPES. If not otherwise stated, the sample was immersion fixed with fixative solution (4% (w/v) PFA EM grade, 0.1 M PB, 0.1 M NaOH in ddH_2_O, pH 7.4) for 1 h at room temperature, followed by ON incubation at 4 °C in the same solution with gentle agitation.

For the screening experiments in Supplementary Fig. 1, organotypic hippocampal slices were incubated with 40–50 µM of the various NHS-conjugated fluorophores for 25–30 min at 37 °C in carbogen-equilibrated ACSF with HEPES with gentle agitation.

For screening of biotin or click chemistry derivatives, live labeling with the respective biotin probes was performed in carbogen-equilibrated ACSF with HEPES for 45 min at 37 °C with gentle agitation, using concentrations as indicated in Supplementary Table 1. After washing and fixation, samples were washed 3× for 30 min each with 1× PBS at room temperature with gentle agitation and permeabilized with 0.2% (v/v) TX in 1× PBS ON at 4 °C with gentle agitation. Samples were washed 3× for 30 min each with 1× PBS at room temperature with gentle agitation and incubated with read-out probe in 1× PBS ON at 4 °C with gentle agitation and then washed 3× for 30 min each in 1× PBS with gentle agitation, followed by confocal imaging.

##### Expansion of organotypic slice cultures with coCATS labeling

Expansion with the MAP approach. After coCATS labeling with an amine-reactive biotin derivative, the sample was ~4-fold expanded according to the MAP protocol^6^ with slight modifications. The sample was immersed in fixative solution (4% (w/v) PFA in 1× PBS) for 10 min at room temperature with gentle agitation. It was then carefully dissociated from the cell culture insert with a brush, placed into MAP solution (30% (w/v) AA, 10% (w/v) PFA, 7% (w/v) SA, 0.1% (w/v) BIS, 0.1% (w/v) VA-044 in ddH_2_O) and incubated ON at room temperature with gentle agitation. The sample was washed 3× for 30 min each with 1× PBS. It was then transferred to a gelation chamber^50^ consisting of a coverslip and two 100-µm-thick spacers placed on a Superfrost slide. The sample was immersed in fresh MAP solution, and a second coverslip was placed on top of the spacers. The gelation chamber was placed in a humidified chamber, and gelation was performed for 1 h at 45 °C. The sample was removed from the gelation chamber and immersed in MAP denaturation solution (200 mM SDS, 200 mM NaCl, 50 mM Tris in ddH_2_O, pH 9). The sample was denatured ON at 37 °C in a humidified environment. Fresh MAP denaturation solution was pre-heated to 70 °C. The sample was immersed in the solution and incubated for 1 h at 70 °C in a water bath. The temperature of the water bath was then increased to 95 °C over 30 min and kept at 95 °C for 1 h. The sample was expanded ~4-fold by immersing in ddH_2_O with three fluid exchanges at 30-min intervals. The gel was trimmed, removing portions not containing biological sample. It was blocked in 5% (w/v) BSA in 1× PBS for 30 min at room temperature with gentle agitation, and CATS signal was read out after incubation with 5 µg ml^−1^ STAR 635P neutravidin in 0.1× PBS ON at room temperature with gentle agitation and re-expansion by incubating 3× for 30 min each in ddH_2_O.

Expansion with proExM. coCATS-labeled organotypic hippocampal slice cultures were ~4× expanded with proExM^8^. After coCATS labeling with an amine-reactive biotin derivative, the sample was immersion fixed with fixative solution (4% (w/v) PFA in 1× PBS) for 1 h at room temperature with gentle agitation and carefully dissociated from the cell culture insert with a brush. It was washed 3× for 30 min each with 1× PBS and permeabilized with 0.2–0.5% (v/v) TX in 1× PBS ON at 4 °C with gentle agitation, followed by washing 3× for 30 min with 1× PBS on an orbital shaker. It was then incubated with 100 µg ml^−1^ AcX in MES buffer (100 mM MES, 150 mM NaCl in ddH_2_O, pH 6) ON at room temperature with gentle agitation, washed 3× for 30 min each in 1× PBS and pre-incubated in ice-cold proExM gelation solution (8.6% (w/v) SA, 2.5% (w/v) AA, 0.15% (w/v) BIS, 11.7% (w/v) NaCl, 0.2% (w/v) TEMED, 0.2% (w/v) APS, 0.01% (w/v) TEMPO in 1× PBS) for 1 h on ice with gentle agitation. The sample was transferred to a gelation chamber consisting of a coverslip and two 100-µm-thick spacers placed on a Superfrost slide, and excess gelation solution was removed. A second, larger coverslip was placed on top of the spacers. Fresh gelation solution was pipetted into the gap between the coverslips until it was filled. The gelation chamber was placed in a humidified chamber, and the sample was incubated for 2 h at 37 °C until gelation was complete. The gelation chamber was disassembled, and the sample stuck to the bottom coverslip was washed once with 1× PBS. The sample was immersed in digestion solution (8 U ml^−1^ proteinase K, 0.8 M guanidine HCl, 50 mM Tris, 2 mM CaCl_2_, 0.5% (v/v) TX in ddH_2_O, pH 8) and incubated for 14–17 h at 50 °C in a humidified environment with gentle agitation. The sample was expanded ~4-fold by immersing in ddH_2_O with three fluid exchanges at 30-min intervals. The sample was trimmed to remove gel portions not containing biological sample, incubated with 5 µg ml^−1^ STAR 635P neutravidin in 0.1× PBS ON at room temperature with gentle agitation and re-expanded by incubating it 3× for 30 min each in ddH_2_O.

##### coCATS viability test

To obtain electrophysiological traces after coCATS labeling, 14–16 DIV slice cultures were first incubated in 0.25% (v/v) DMSO (control) or 50 µM STAR RED-NHS (dye incubation) in carbogen-equilibrated ACSF with HEPES for 25 min at 37 °C with gentle agitation. Slices were briefly washed with carbogen-equilibrated ACSF with HEPES, and 3–4 CA1 PNs per slice were recorded.

To obtain functional recordings during coCATS labeling incubation, CA3 PNs from a 16-DIV organotypic hippocampal slice culture were first recorded for 10 min. ACSF was exchanged with dye solution (50 µM STAR RED-NHS in ACSF), and the recording was continued for another 20 min without solution flow to reproduce the coCATS labeling procedure.

##### coCATS labeling of cerebral organoids

Cerebral organoids were incubated in 40 µM STAR RED-NHS in pre-warmed carbogen-equilibrated ACSF containing HEPES for 30 min at 37 °C with gentle agitation. The samples were briefly washed with fresh ACSF solution and fixed with 4% (w/v) PFA in 0.1 M PB and 0.1 M NaOH, pH 7.4 for 1 h at room temperature with gentle agitation. The samples were washed 3× for 30 min each in 1× PBS before imaging.

#### rCATS

##### rCATS labeling of fixed brain tissue

Mouse brain tissue. Coronal brain sections of 100–200-µm thickness obtained from PFA fixative-perfused animals were permeabilized by repeated freeze–thaw cycles. For this, they were washed 3× for 30 min each with 1× PBS and incubated with 30% (w/v) sucrose in 1× PBS ON at 4 °C with gentle agitation. Sections were placed on a Superfrost slide, immersed in sucrose solution and positioned on dry ice until the solution was completely frozen and then removed and left to thaw at room temperature. Freeze–thawing was repeated 4–5 times. Sections were then washed 3× for 30 min and immunostained as described above. To achieve rCATS labeling, the secondary AB solution was complemented with 5–8 µg ml^–1^ WGA CF633 plus 0.02% (w/v) NaN_3_, and the samples were incubated for 20 h at room temperature with gentle agitation. Alternatively, we used permeabilization by ON incubation with 0.2% (v/v) TX (concentration unless otherwise noted) in 1× PBS at 4 °C with gentle agitation as indicated for specific experiments.

Human brain surgery specimens. We obtained surgical specimens of hippocampus or cortex from eight individuals and applied rCATS. The data in Fig. 6a–c are from the cerebral cortex of a 35-year-old male patient undergoing hippocampal surgery for treatment of epilepsy, diagnosed with sclerosis of the hippocampus. The data shown in Supplementary Fig. 24 are from a hippocampal brain section from a 36-year-old male patient diagnosed with epilepsy with sclerosis of the hippocampus. Previously to the temporal lobe surgery, during which tissue used here was extracted, a brain tumor was removed. Histology of the hippocampus showed no presence of neoplastic tissue. Immediately after resection, the tissue samples were either transferred to physiological saline (0.9% (v/v) NaCl in water) and fixed by immersion in 4% (w/v) PFA within 5 min and post-fixed on an orbital shaker at 4 °C ON, or they were transported from the operating theater to the laboratory (55 min) in sucrose-based ACSF (containing 64 mM NaCl, 25 mM NaHCO_3_, 2.5 mM KCl, 1.25 mM NaH_2_PO_4_, 10 mM glucose, 120 mM sucrose, 0.5 mM CaCl_2_ and 7 mM MgCl_2_), equilibrated by bubbling with carbogen (95% O_2_/5% CO_2_) before immersion fixation in 4% (w/v) PFA ON at 4 °C. After PFA fixation, the tissue was washed with 1× PBS 3× for ≥15 min each. The samples were embedded in 3% (w/v) agarose and sliced with a vibratome (Leica VT 1200 S) at 100–200-µm thickness. The vibratome slices were cryoprotected with 30% (w/v) sterile-filtered sucrose until they sunk in the solution. Samples were stored at −80 °C until further use. The tissue was brought to room temperature and permeabilized by freeze–thawing five times. Samples were washed 3× for 30 min each with 1× PBS and blocked with 5% (w/v) BSA, 1% (v/v) NGS and 0.02% (w/v) NaN_3_ in 1× PBS for 4 h at room temperature with gentle agitation. They were then incubated with primary ABs in 5% (w/v) BSA, 1% (v/v) NGS and 0.02% (w/v) NaN_3_ in 1× PBS for 20 h at room temperature with gentle agitation. They were washed 3× for 30 min each with 1× PBS with gentle agitation and incubated with secondary ABs in 5% (w/v) BSA, 1% (v/v) NGS and 0.02% (w/v) NaN_3_ in 1× PBS for 20 h at room temperature with gentle agitation. The samples were washed 3× for 30 min each with 1× PBS with gentle agitation and then incubated with 5–6 µg ml^−1^ WGA CF633 in 1× PBS ON at room temperature with gentle agitation. The samples were washed 3× for 30 min each with 1× PBS.

Human archival brain tissue. For pathohistological evaluation, archival FFPE tissue blocks were sectioned at 4-µm thickness on a microtome, transferred to a water bath and mounted on Superfrost glass slides. H&E (Supplementary Fig. 25b) and Luxol fast blue (Supplementary Fig. 26c) stainings were performed according to standard protocols. Immunohistochemistry stainings (Supplementary Figs. 26 and 27) with macrophage marker CD68 and axon marker neurofilament H were performed after deparaffinization and rehydration. Sections were first incubated in 3% hydrogen peroxide in methanol to block endogenous peroxidase activity, followed by antigen retrieval in pH 6 citrate buffer. Sections were incubated with the primary ABs in antibody diluent/blocking solution (DAKO) overnight at 4 °C. Samples were washed 3× with Tris/HCl buffer, followed by 25-min incubation at room temperature with EnVision Flex+ kit as detection system (DAKO). 3′,3-diaminobenzidine as chromogen was used for 10 min at room temperature to visualize antibody reaction. Slides were digitalized on a NanoZoomer 2.0-HT digital slide scanner C9600 (Hamamatsu Photonics), and NPD.Viewer2 was used for export to .tiff files.

For rCATS, archival FFPE tissue blocks were sectioned at 6–8-µm thickness on a microtome, transferred to a water bath and mounted on silane-coated coverslips. Coverslip coating was performed with (3-aminopropyl)triethoxysilane according to the manufacturer’s instructions. In brief, coverslips were cleaned with soap and distilled water. After drying, they were incubated in 2% silane solution (prepared freshly as 2 ml of silane in 98 ml of acetone and stirred for 5 min) for 2 min at room temperature, followed by washing twice with fresh acetone and air-drying. After mounting, sections were air-dried and then baked at 60 °C for 2.5–4 h. They were deparaffinized twice for 5 min with xylol with gentle agitation, followed by two 5-min washes in 100% EtOH. Sections were successively rehydrated in 96%, 80% and 70% EtOH in ddH_2_O for 5 min each and washed twice for 5 min with ddH_2_O. Antigen retrieval was performed for 20 min at 95 °C with a low-pH (35.8 mM citric acid, 128.4 mM Na_2_HPO_4_ in ddH_2_O, pH 6.0) or high-pH (10 mM Tris, 1 mM EDTA in ddH_2_O, pH 9.0) antigen retrieval solution, depending on requirements for subsequent antibody staining. Samples were left in antigen retrieval solution for 30 min at room temperature to cool down. They were washed twice for 5 min with ddH_2_O and blocked with 5% (w/v) BSA, 1% (v/v) NGS, 0.02% (w/v) NaN_3_ in 1× PBS for 2 h at room temperature in a humidified environment. Primary AB incubation was performed in 5% (w/v) BSA, 1% (v/v) NGS, 0.02% (w/v) NaN_3_ in 1× PBS for 2 h at room temperature in a humidified environment, followed by washing 3× for 5 min with 1× PBS. Sections were then incubated with secondary ABs and 5 µg ml^−1^ WGA in 5% (w/v) BSA, 1% (v/v) NGS, 0.02% (w/v) NaN_3_ in 1× PBS ON at room temperature in a humidified environment, washed 3× for 5 min with 1× PBS and mounted on Superfrost slides in Fluoromount G.

Overview imaging was performed on a confocal microscope (Leica Sp8 inverted or Sp8 upright) with an air objective (Leica HC PL Fluotar ×10/NA 0.3/ WD 11.0 mm). Higher-magnification overview images were acquired with a water immersion objective (Leica HC Fluotar ×25/NA 0.95/WD 2.5 mm or Leica HC PL APO ×40/NA 1.10/WD 0.65 CORR CS2). High-resolution confocal and STED images were acquired with a water (Olympus UPLSAPO60XW ×60/NA 1.20/WD 0.28) or silicone oil immersion (Olympus UPLSAPO100XS ×100/NA 1.35/WD 0.20 mm) objective on an Abberior Expert Line STED microscope.

Characterizing the effect of fixation and tissue permeabilization on rCATS labeling. For whole-hemisphere immersion fixation of mouse brain (Supplementary Fig. 20a), the brain was excised after killing the animal and hemispheres were separated, immersed in fixative solution (4% PFA in 0.1 M PB, 0.1 M NaOH, pH 7.4) and fixed for 20 h at 4 °C before rCATS labeling using WGA-CF633.

To characterize the effect of detergent permeabilization on rCATS labeling (Supplementary Fig. 20), mouse brains from freshly perfused animals were cut into 300-µm-thick coronal sections. The sections were permeabilized with the following conditions: (1) five freeze–thaw cycles; (2) 0.5% (v/v) TX in 1× PBS at 4 °C for 20 h with gentle agitation; or (3) 0.5% (v/v) TX in 1× PBS at room temperature for 20 h with gentle agitation. The effect of detergent in the labeling solution was tested by adding or omitting 0.1% (v/v) TX to the solution containing 4 µg ml^−1^ WGA CF633 in 5% (w/v) BSA + 1% (w/v) NGS + 0.02% (w/v) NaN_3_ in 1× PBS and incubating for 20 h at room temperature with gentle agitation.

rCATS labeling with expansion in previously fixed mouse brain. rCATS-labeled brain tissue was ~4-fold expanded with proExM^8^. After pre-expansion imaging of eGFP signal on a spinning-disc confocal microscope, the 100-µm-thick brain sections from PFA fixative-perfused adult mouse were permeabilized with 0.2% (v/v) TX in 1×PBS ON at 4 °C and immunostained as described above. They were incubated with 16.7 µg ml^−1^ WGA-biotin in 1× PBS for 20 h at room temperature with gentle agitation, washed 3× for 30 min each with 1× PBS and then incubated with 20 µg ml^−1^ streptavidin acrylamide for 20 h at room temperature with gentle agitation. Samples were washed 3× for 30 min each with 1× PBS, followed by anchoring with 100 µg ml^−1^ AcX in MES buffer (100 mM MES, 150 mM NaCl in ddH_2_O, pH 6) ON at room temperature with gentle agitation. They were washed 3× for 30 min each in 1× PBS with gentle agitation and pre-incubated in ice-cold proExM gelation solution (8.6% (w/v) SA, 2.5% (w/v) AA, 0.15% (w/v) BIS, 11.7 % (w/v) NaCl, 0.2% (w/v) TEMED, 0.2% (w/v) APS, 0.01% (w/v) TEMPO in 1× PBS) for 1 h on ice with gentle agitation. Samples were transferred to a gelation chamber consisting of a coverslip and two 100-µm-thick spacers on a Superfrost slide. Excess gelation solution was removed. A second coverslip was placed on top of the spacers. The gap between the coverslips was then filled with fresh gelation solution. The gelation chamber was placed in a humidified environment for 2 h at 37 °C until gelation was complete and carefully disassembled. Samples were washed with 1× PBS and immersed in digestion solution (8 U ml^−1^ proteinase K, 0.8 M guanidine HCl, 50 mM Tris, 2 mM CaCl_2_, 0.5 % (v/v) TX in ddH_2_O, pH 8), incubated for 14–17 h at 50 °C in a humidified environment with gentle agitation and washed 3× for 1 h each in 1× PBS at room temperature with gentle agitation. They were incubated with 6 µg ml^−1^ ATTO643 biotin in 0.1% (v/v) TX in 1× PBS ON at room temperature with gentle agitation and washed with 1× PBS. Samples were ~4× expanded by immersing in ddH_2_O with three fluid exchanges at 30-min intervals.

#### Imaging

Data were acquired on the following microscopes. A detailed summary of the labeling and imaging parameters for each dataset can be found in Supplementary Table 1. Power values refer to the power at the sample determined with a slide powermeter head (Thorlabs, S170C).

##### Confocal imaging

Confocal imaging was performed for volumetric imaging of expanded organotypic slice cultures and overview imaging of expanded brain slices from fixative-perfused animals with a Leica Sp8 inverted microscope with a super-continuum pulsed white light laser (for excitation at 490 nm and 630 nm), a 405 nm continuous-wave diode laser and HyD GaAsP detectors. Imaging was performed with a ×40 water objective (Leica HC PL APO ×40/NA 1.10 W CORR CS2, WD 0.65 mm) using Leica LAS X software version 2.5.7.23225.

##### Spinning-disc confocal microscopy

Spinning-disc confocal microscopy was performed on an Andor Dragonfly featuring a Nikon Ti2E inverted microscope with motorized stage, a spinning disc with two pinhole disc patterns (25-µm and 40-µm hole diameter) and four continuous-wave excitation lasers (405 nm, 488 nm, 561 nm and 637 nm) and an Andor Zyla 4.2 Megapixel sCMOS camera. Overview images of brain sections from fixative-perfused animals were acquired with a ×10 air objective (Nikon CFI P-Apo ×10 lambda/NA 0.45/WD 4.0 mm). Overview images of organotypic slice cultures were acquired with a ×20 air objective (Nikon CFI P-Apo ×20 lambda/NA 0.75/WD 1.0 mm). Volumetric data of expanded rCATS specimens were acquired with an LWD ×20 water objective (Nikon CFI P-Apochromat ×20/NA 0.95/WD 0.95 mm) or a ×40 water objective (Nikon Apochromat LWD ×40 lambda S/NA 1.15/water/WD 0.6 mm). Andor Fusion software version 2.2 was used for data acquisition and stitching of tiles, unless otherwise stated.

##### STED imaging

Confocal and STED imaging were performed on an inverted STED microscope (Abberior Instruments, Expert Line) using a ×60 water immersion objective (Olympus UPLSAPO60XW ×60/NA 1.20/WD 0.28 mm) or a ×100 silicone oil objective (Olympus UPLSAPO100XS ×100/NA 1.35/WD 0.20 mm), both with correction collar, and pulsed lasers (excitation: 488 nm, 561 nm, 640 nm; STED: 775 nm; pulse repetition rate: 40 MHz) with time-gated fluorescence detection using photon-counting avalanche photodiodes (APDs) and bandpass filters at 525/50 nm (Semrock, F37-516), 605/50 nm (Chroma, F49-605) and 685/70 nm (Chroma, F49-686). Galvanometric mirrors and a sample piezo stage (Physik Instrumente, P-735.ZRO) were used for lateral and axial scanning, respectively. Instrument control was performed with Imspector versions 14.0.3052 or 16.3.13031.

For confocal imaging, typically a pinhole size of 0.5–1.0 Airy units, 10–20-µs dwell time and 1–2 line accumulations were used.

Typical parameters for single-plane STED imaging with lateral resolution enhancement were: 10–20-µs dwell time, 3–5 line accumulations, 0.8–6.0-µW (561 nm) and 0.5–5.0-µW (640 nm) excitation power and 24–100-mW STED power. The 488-nm excitation channel was used in confocal mode with 0.5–5.0-µW excitation power. Pixel size was 30 × 30 nm^2^ or 50 × 50 nm^2^. A spatial light modulator was used for 2π-helical phase modulation to generate the *xy*-STED pattern and to partially compensate for aberrations.

Typical parameters for volumetric *z*-STED imaging were: 10–15-µs dwell time, 2–3 line accumulations, 1–2-µW (561 nm) and 0.3–2.6-µW (640 nm) excitation power and 43–88-mW STED power and voxel size 50 × 50 × 50 nm^3^. The 405-nm and 488-nm excitation channels were used in confocal mode with 6.8–16.0-µW and 0.2–5.0-µW excitation power, respectively. The spatial light modulator was used to create a π-top-hat phase modulation (*z*-STED), predominantly increasing axial resolution. In Fig. 6c, subpanels of Extended Data Figs. 3 and 4, Extended Data Figs. 6 and 9b and Supplementary Figs. 14 and 18, near-isotropically resolved STED data were collected with a combination of z-STED (80%) and 4π-helical phase modulation (20%) patterns according to ref. ^24^. Volumetric imaging was typically performed with all power assigned to the *z*-STED pattern and in *xzy*-scan mode, with *y* being the slowest scan axis. For STED imaging, pinhole size was 0.5–0.9 Airy units. Acquisition was tiled with a custom-written Python script controlling sample stage position, and tiles were stitched with the Fiji plugin ‘Grid/Collection stitching’.

### Data analysis

#### Visualization

Lookup tables of CATS images were inverted using ImageJ/Fiji^64^ version 1.53f51, unless otherwise stated. Display ranges were adjusted for visualization. Intensity lookup tables were linear, unless stated otherwise. For displaying CATS plus immunostaining images, channels were saved separately in RGB format. Using GIMP version 2.10.30, black was set to transparent (alpha = black), and immunostainings were overlayed with the CATS data. Lucifer yellow signal collected in two spectral channels was added up with the Fiji/Calculator Plus plugin. 3D visualizations were done with Blender 2.92 (https://www.blender.org/) unless otherwise noted. Schematics in Fig. 1a and Supplementary Figs. 2a and 22a were created with BioRender (https://www.biorender.com/).

#### Denoising

Volumetric datasets were denoised with N2V version 0.2.1 (ref. ^36^) with the following parameters, unless otherwise stated: 3D mode, patch size 16 × 32 × 32 or 32 × 32 × 32 (*zyx*); number of patches per image: all; patches augmented eight times by rotation and axis mirroring; neighborhood radius: 5; percent pixel manipulation: 1.5; number of epochs: 75–80; number of steps per epoch: 100; batch size: 8–16, using a workstation with Intel Xeon W ‘Skylake’ W-2145, 3.60-GHz processor, 128 GB RAM and NVIDA GeForce RTX 2080Ti graphics card. Software was installed from GitHub (https://github.com/juglab/n2v). Results were visually inspected for artifacts from denoising before further processing. Raw data are displayed in Supplementary Figs. 4 and 21 for comparison.

#### Manual segmentation

Datasets for 3D renderings were five-fold upsampled in the lateral directions with nearest-neighbor interpolation using ImageJ/Fiji to ease manual segmentation, resulting in 10 ×10 × 50-nm voxel size. Manual segmentation was performed in VAST^65^ version 1.4.0, downloaded from https://lichtman.rc.fas.harvard.edu/vast/.

#### Identification and segmentation of pSCRs via immunostaining

coCATS data after in vivo stereotactic injection (Fig. 2, analysis of three imaging volumes) were analyzed with custom-written Python version 3.7.12 pipelines implemented with JupyterLab version 3.2.4. For visual inspection, Napari version 0.4.12 (10.5281/ZENODO.3555620) was used.

##### BASSOON and SHANK2 segmentation

For background removal and smoothing, datasets for immunostained BASSOON (confocal) and SHANK2 (STED) were smoothed with two different Gaussian filters using scipy.ndimage.gaussian_filter with sigma of 15 and 1 voxel, corresponding to background and signal, respectively. Background was then subtracted from signal, and negative values were clipped to zero. Resulting images were transformed into binary masks by global Otsu thresholding. No N2V was applied for segmentation.

##### pSCR segmentation

After denoising of 3D CATS data with N2V, contrast stretching with the skimage.exposure.rescale_intensity function was performed using the 1st and 99th intensity percentile (imaging volumes 1 and 2) or the 1st and 98th percentile (imaging volume 3) as limits. Next, the SHANK2 mask was dilated using skimage.morphology.binary_dilation with a ball of 2-voxel radius. Volumetric CATS data were then multiplied with the corresponding dilated binary SHANK2 mask to isolate regions containing pSCRs. Then, a global threshold was applied to the resulting image to create a binary CATS mask. The global threshold (*thr*) was computed individually for each dataset and was set between the 95th percentile (*p95*) and 100th percentile (*p100*) of voxel brightness, as judged by visual inspection using *thr* = p95 + *s* × (p100 − p95), with *s* = 0.65 for imaging volumes 1 and 2 and *s* = 0.6 for imaging volume 3. To obtain instance segmentations, the connected components of the CATS mask were labeled using the skimage.measure.label function. Objects smaller than 8 voxels were discarded.

##### Co-localization and association with MFBs

We classified segments obtained from the CATS channel as pSCRs in case of spatial overlap with both SHANK2 and BASSOON. For this, we first defined the overlap region between the BASSOON and non-dilated SHANK2 masks and retained CATS segments as pSCRs that had at least 1-voxel overlap with this intersection region. We next associated pSCRs with individual MFBs. MFB volume segmentations were performed manually as described above and scaled back to the original voxel size (5× downscaling in lateral dimensions). MFB masks were dilated using skimage.morphology.binary_dilation with a ball of 2-voxel radius. Then, pSCRs that overlapped with dilated MFBs (at least 1 voxel) were extracted and assigned to that bouton.

##### Manual proofreading

pSCR segments were manually proofread and corrected in VAST. For processing in VAST, MFB and pSCR segmentations were combined, conserving manually segmented MFB shape. pSCRs were subsequently proofread, and empty voxels between pSCR segments and manually created MFB segments were filled in.

#### MFB quantification

For single-bouton quantification in Fig. 2c–g, 10 MFBs each from three volumetric datasets containing coCATS, BASSOON and SHANK2 labeling were selected based on their characteristic shape and presence of pSCRs sandwiched between BASSOON and SHANK2 staining. MFB volumes were manually segmented. The Scikit-image implementation of the marching cubes algorithm was used to create 3D meshes of individual MFBs from volume segmentations. Bouton surface area A_MFB_ and volume V_MFB_ were computed using a custom-written script in Blender 2.92. Bouton volumes were computed with bmesh.calc_volume, and areas were computed as the sum of mesh face areas.

Areas of MFBs occupied by pSCRs were found using a custom Python script. For each 3D segmented pSCR, voxels that touched the bouton segment were extracted to define the pSCR–MFB contact area, and their coordinates were converted into point cloud data (pcd) format using the open3d library. The ball-pivoting algorithm from the open3d library with two radii (1 voxel and 1.5 voxels) was used to create a triangle mesh from the point cloud. Minor corrections to the surfaces were made in Blender where necessary. The area of contact between a pSCR and an MFB was computed as sum of the corresponding mesh face areas. The total area of individual boutons occupied by pSCRs (A_pSCR/MFB_) is the sum of the pSCR–MFB contact areas of all pSCRs connected to one bouton.

#### Deep-learning-based prediction of synapse location

To predict synapse location purely from coCATS data, immunostained synaptic markers in the pSCR segmentation pipeline were replaced by a deep-learning-based prediction of molecule location. Here, instead of using super-resolved images of immunostained SHANK2 to guide pSCR segmentation, we used super-resolved BASSOON location predicted from the CATS channel. To predict BASSOON location, we used image translation with a U-Net convolutional neural network^41^ trained with super-resolved coCATS and BASSOON immunostaining data. Code was adapted from https://github.com/Li-En-Good/VISTA (ref. ^30^).

##### Data pre-processing

Thirteen STED volumes containing BASSOON immunostainings and coCATS were used for training (~85,000 µm^3^). CATS volumes were denoised with N2V and converted to 16-bit format. For background subtraction and denoising of the BASSOON channel, two different Gaussian filters using scipy.ndimage.gaussian_filter with sigma of 15 and 1 voxel were applied, corresponding to background and signal channels, respectively. Background was subtracted from signal, and any negative values were set to zero. For BASSOON immunostainings, no N2V was applied for training.

##### Training

The image translation network was trained for 10,000 iterations with batch size 8, buffer size 5 and patch size 64 × 64 × 32 (*xyz*). Training was performed on a single node of a high-performance computing cluster available at ISTA, with an Intel Xeon CPU E5–2680 v4 @ 2.40-GHz CPU and 256 GB RAM, assigning up to 64 GB RAM and one CPU according to availability. Training took ~2 h, accelerated with a single GPU (GeForce RTX 2080 Ti). Prediction took ~3.5 min.

##### Segmentation

For automatically segmenting pSCRs followed by manual proofreading (Figs. 3 and 4 and Supplementary Figs. 8g (pSCRs_immuno/proofread_), 11 and 13), the predicted super-resolved BASSOON signal was thresholded to obtain a binary mask, dilated using skimage.morphology.binary_dilation with a 2-voxel radius ball and multiplied with the corresponding coCATS data to isolate regions containing pSCRs. CATS data were contrast stretched and binarized by thresholding at *thr* = p93 + 0.4 × (p100 − p93), with p93 being the 93rd intensity percentile.

For direct quantification of automatically generated pSCR segments (Fig. 2i–k and Supplementary Fig. 8a–i), a refined local thresholding procedure was established. Here, 3D CATS data were processed with N2V and contrast stretched, and then a smoothing/background subtraction step was performed by convolution with two Gaussians with sigma of 7 and 0.3 voxels, corresponding to background and signal channels, respectively. The background channel was subtracted from the signal channel, and negative values were clipped to zero. To account for the blurring effect of N2V and background subtraction, a grayscale erosion step with skimage.morphology.erosion with a ball of 1-voxel radius was performed. We then used skimage.filters.threshold_multiotsu with three classes, taking the higher of two outputs for thresholding of CATS data. We selected segments by multiplying with the corresponding BASSOON mask (generated from immunolabelings or prediction via Otsu thresholding), dilated with a ball of 1-voxel size. Finally, we obtained instance segmentations with skimage.measure.label and removed objects smaller than 6 voxels.

##### Validation of deep-learning-based synapse prediction

Validation metrics. Voxel-based and object-based metrics were used to assess performance of the synapse prediction model, using a test dataset (224 × 224 × 96 voxels) not included in the training, whose size was chosen to account for the specific model architecture.

For voxel-based evaluation, the Pearson correlation coefficient *r* between BASSOON location predicted from CATS images and BASSOON immunostaining was computed using the numpy.corrcoef function in Python.

For object-based evaluation, pSCR segments based on predicted BASSOON (pSCRs_prediction_) and pSCR segments based on immunostained BASSOON (pSCRs_immuno_) of the same region were compared using the F1 score (Fig. 2k and Supplementary Fig. 8). First, corresponding objects from immunostaining-derived and prediction-derived segmentations were found based on spatial overlap of at least 1 voxel. For each pair, the IOU (ratio of overlapping volume versus combined volume) was computed. We additionally give the Dice coefficient (2× the number of voxels in the overlapping volume divided by the sum of the number of voxels of the individual volumes), as indicated. If a segment from the immunostaining pipeline overlapped with more than one segment from the deep-learning-based pipeline, the immunostaining/deep learning segment pair with the larger IOU or Dice coefficient was retained. The number of true positives (*N*_TP_) was determined as the number of pSCRs_prediction_ segments with a corresponding pSCRs_immuno_ segment having IOU (Dice coefficient) above threshold. Conversely, if the IOU (Dice coefficient) was below this threshold, they were considered as false positives (*N*_FP_). The number of false negatives (*N*_FN_) was determined as the number of pSCRs_immuno_ segments that did not have a corresponding pSCRs_prediction_ segment with IOU (Dice coefficient) above threshold. For calculating F1, the threshold was set to 0.2. Precision (*P*), recall (*R*) and F1 (ranging from 0 to 1) were calculated as a function of IOU (Dice coefficient) threshold according to

$$P\,=\,\frac{{N}_{{\rm{TP}}}}{{N}_{{\rm{TP}}}+{N}_{{\rm{FP}}}}$$

$$R\,=\,\frac{{N}_{{\rm{TP}}}}{{N}_{{\rm{TP}}}+{N}_{{\rm{FN}}}}$$

$${\rm{F}}1\,=\,2\bullet \frac{P\bullet R}{P+R}$$

Effect of denoising on deep-learning-based synapse prediction. To study the effect of the N2V algorithm on performance of deep-learning-based synapse prediction (Supplementary Fig. 8c,d), a network was trained with raw instead of N2V-denoised CATS data and super-resolved BASSOON. Processing for the BASSOON immunostaining channel was identical as before. CATS pre-processing included or omitted N2V, and then contrast stretching and background subtraction were applied with Gaussians of sigma = 7 voxels for the background channel and 0.3 and 0.5 voxels for N2V and raw data, respectively, in the signal channel. Grayscale erosion and multi-Otsu thresholding were performed as before.

Effect of super-resolution imaging on deep-learning-based synapse prediction. To compare performance of deep-learning-based synapse prediction for super-resolution versus diffraction-limited acquisition of the molecular ground truth channel (Supplementary Fig. 8e,f), we trained a model with super-resolved coCATS data using either super-resolved or confocal data of immunostained BASSOON as molecular ground truth, with ~65,000-µm^3^ overall training volume for each, and used the respective predictions for pSCR detection in CATS data.

#### Reconstruction of local synaptic input field

For reconstruction of synaptic input onto a CA3 PN proximal dendrite (Fig. 3), coCATS data were denoised with N2V. The positively labeled dendrite was manually segmented based on super-resolved coCATS data. Then, all structures forming synaptic connections with the proximal dendrite were identified by presence of pSCRs in CATS data and segmented. 3D meshes were created as described above and 5× upsampled axially in Blender to account for lateral upsampling during manual segmentation.

To extract spines, the dendrite segment was morphologically opened (erosion followed by dilation) with skimage.morphology.binary_opening (ball with 3-voxel radius) to detach spines from the main branch. The largest fragment (main branch) was dilated with skimage.morphology.binary_dilation by 1 voxel and subtracted from the original PN segment, yielding a binary mask containing all complex spines, which was converted to an instance segmentation with skimage.measure.label. Minor manual corrections to the resulting objects were done in VAST. Spine meshes were generated with the marching cubes algorithm and imported into Blender to determine their volumes as described above.

MFBs were identified by the following characteristics: (1) bulbous enlargement in close proximity to the dendrite and (2) one or multiple contacts (pSCRs) with spines of the dendrite. An axon segment associated with the bouton was typically running alongside other axons in a mossy fiber bundle, often roughly perpendicular to the PN proximal dendrite. Segmented structures that did not fulfill these characteristics were classified as non-MFB structures. Only structures situated completely or near-completely (≥80% as judged from lower-resolution overview images) in the imaging volume were included.

To measure volume of MFBs (Fig. 3j), axons and filopodia were detached from the main bouton bodies by erosion and dilation adapted for each object, followed by manual corrections in VAST, generation of 3D meshes and computation of volumes as before.

We used the image translation model trained on super-resolved CATS and BASSOON in Fig. 2j for pSCR segmentation (Supplementary Fig. 11). For prediction of BASSOON location, the CATS dataset in Fig. 3 (456 × 530 × 160 voxels) was denoised with N2V and cut into overlapping 3D patches (224 × 224 × 96 voxels). Predictions of BASSOON location for individual patches were combined and fed into the pSCR segmentation pipeline without the background subtraction and grayscale erosion steps, applying contrast stretching (1st and 98th intensity percentile) and thresholding at *thr* = p96 + 0.65 × (p100 − p96) to CATS data, followed by manual proofreading.

To quantify interactions between MFBs and spines (Fig. 3h,i), an interaction was identified if one or more pSCRs connected these two structures. For each pSCR, a list was generated with MFBs and spines having at least 1-voxel overlap with the respective pSCR mask dilated by a ball of 1-voxel radius. If a pSCR displayed overlap with more than one MFB, the one with the largest overlap was retained. These lists were used to determine the number of MFBs interacting with each spine and number of spines interacting with each MFB.

#### Reconstruction of synaptic output field

For reconstruction of the synaptic output field in Fig. 4d,e, the positively labeled DG granule cell axon was segmented from the super-resolved intracellular channel using Otsu thresholding. In Fig. 4d, the resulting binary mask was additionally eroded with skimage.morphology.binary_erosion by 1 voxel. Structures forming synaptic connections with the displayed boutons of the positively labeled cell were identified via existence of pSCRs in coCATS data and manually segmented without upsampling. pSCR segmentation was performed using image translation as described in the previous subsection, where CATS channels were contrast stretched using the 1st and 99th intensity percentile and binarized using *thr* = p95 + 0.65 × (p100 − p95). To associate pSCRs with the DG granule cell, the segmentation masks of this positively labeled neuron were dilated using skimage.morphology.binary_dilation with a 2-voxel-radius ball in each imaging volume, and pSCRs that overlapped (at least one voxel) with dilated masks were retained. 3D meshes of the segmented positively labeled DG granule cell axon, associated post-synaptic structures and pSCRs were processed with Blender for visualization.

#### rCATS ExM data processing

We used the Fiji plugin BigStitcher version 0.8.3 (ref. ^66^) to create a BigStitcher dataset with the Automatic Loader (Bioformats) option and arranged tiles with the ‘Move tiles to regular grid’ option with 10% overlap in each dimension. For alignment and visualization in BigDataViewer^67^, volumes were re-saved (using default options) to the chunked, pyramidal image format N5 with six pre-computed resolutions levels. Using BigStitcher, we used the ‘average over channels’ option for pairwise shift calculation between adjacent tiles, which we computed at 2× subsampled resolution. Only shifts resulting in a Pearson correlation coefficient exceeding 0.7 were used in the global optimization, where we used the ‘Identify wrong links and handle unconnected tiles, STRICT’ option. We fused and exported aligned tiles to HDF5/BigDataViewer files as 16-bit unsigned integer type with tri-linear interpolation and smooth image blending for fusion. To not exceed the available computer memory of 128 GB, we used the ‘Cached’ image option. For HDF5 creation, we used BigStitcher’s default parameters.

For denoising the post-expansion image, we used custom Python scripts based on the BigDataViewer/N5 dataset and N2V (version 0.2.1). For training, 6,000 patches of size 64 × 64 × 64 (*xyz*) were randomly sampled across the entire volume per color channel. We trained for 60 epochs with 256 steps per epoch. Each channel was trained independently, yielding an N2V model per channel. We predicted the denoised output for each N5 chunk in parallel with SLURM on a high-performance cluster with GPU acceleration using a custom Python script.

To determine the expansion factor, manual alignment of the pre-expansion and post-expansion volumes was performed with BigWarp^68^ by matching landmarks in the eGFP channels in 3D. The effective expansion factor was extracted from BigWarp’s landmark file using a custom Fiji/Groovy script.

For manual skeletonization, the denoised multi-channel post-expansion volume was converted from BigDataViewer/HDF5 format to the webKnossos data structure. Because the volumes exceeded available main memory of 128 GB, we created custom Python scripts based on the webKnossos Python library (version 0.10.5) to convert image data block-wise.

Skeletonization of a mossy cell (Fig. 5i) was performed with webKnossos (version 22.05.1)^69^ installed on a local server (https://docs.webknossos.org/webknossos/installation.html, section: Installation on your Server) with slight modifications. Specifically, we explicitly attached the ports for both the datastore (9000) and tracingstore (9000) and started ‘Docker’ through Docker-compose specifying the Docker tag 22.05,1. Due to the large size of the dataset, we used file system transfer. The local server ran on a 2× AMD EPIC MILAN 75F3 processor, 32-core 32C/64T, 2.95 GHz with 512 GB of memory, 2 TB Intel NVMe-SSD and 4× NVIDIA A6000 GPUs. A cell soma in the center of the volumetric dataset was skeletonized with the webKnossos-skeleton tool with orthogonal views.

#### Axon tracing

We quantified the distance over which axons in the CA3 stratum lucidum could be traced purely from coCATS data. For this, in vivo microinjection of STAR RED-NHS into the LV of an adult *Thy1-eGFP* mouse was performed as described, followed by transcardial fixative perfusion. Coronal sections were immunolabeled for eGFP with AF594. Volumetric datasets were acquired with near-isotropic STED resolution to super-resolve both the coCATS and the sparse intracellular label. Both color channels were denoised with N2V, and single seeds for skeletonization were placed in 10 eGFP-positive axons in webKnossos. Then, a tracer received the coCATS dataset with the seed points but without the positive label, such that they were blinded to the eGFP signal. The person traced the axons from the coCATS data in both directions from the seed points with webKnossos. A second person then inspected the dataset using the sparse positive label as reference. Starting from the seed, the skeleton was followed in both directions until the first incorrectly placed skeleton node was encountered. This node, and all subsequent nodes, were deleted. The lengths of the resulting traces were measured and quantified.

#### Dense reconstruction of human cerebral organoid volume

The 3D dataset was denoised using N2V, and intensity was normalized to the maximum value. To account for a slight gradient in image intensity with depth, adaptive histogram equalization was implemented with a custom Python script based on the skimage.exposure.equalize_adapthist function. The clip limit was set to 0.02 and the kernel size to 1/5 of the stack size in each dimension. Cellular structures were manually segmented as described and visualized using the 3D viewer in VAST.

### Statistics and reproducibility

#### Experimental reproducibility

In all images, representative data from single experiments are shown. To confirm reproducibility of the technology, we performed a series of technical replicates that were typically recorded across several biological specimens, as indicated below. For some of the procedures that we performed routinely, such as in vivo microinjection, the stated number of replicates gives a lower bound, and we did not count additional replicates beyond *n* = 10. For high-quality representation of tissue structure, optimum tissue preservation, labeling and imaging conditions are required. We discarded datasets that were of lower quality.

Figure 1: Fig. 1b: Data are representative of coCATS experiments in *n* = 10 organotypic hippocampal slices and rCATS in *n* = 10 fixative-perfused animals. Figure 1c,d,e: Images are representative of coCATS with in vivo microinjection into LV in *n* = 10 animals. Figure 2: Fig. 2a: Imaging data are representative of in vivo microinjection into the LV in *n* = 10 animals. Figure 2b–g: Renderings and quantitative analysis of n_MFB_ = 30 MFBs reconstructed (10 from each of three imaging volumes recorded across two brain sections (one animal)); 22 MFBs are displayed in Fig. 2b and eight MFBs in Supplementary Fig. 7. Figure 2h represents one of the three imaging volumes used for MFB visualization and quantification. Figure 2j,k: Training was performed on *n* = 13 imaging volumes recorded across four brain sections from *n* = 3 animals and testing on *n* = 1 dataset. Figure 3: Fig. 3a,b: Imaging data are representative of coCATS in *n* = 10 organotypic slices. Figure 3c–g: Data are representative of coCATS labeling in combination with functional recordings and dye filling of various cell types in *n* = 6 organotypic slices. Figure 3h,i: 3D reconstruction was performed for *n* = 1 specimen, and analysis in Fig. 3j–l comprised one dendrite with n_spine_ = 68 spine structures and n_MFB_ = 40 MFBs. Three additional MFBs were only partially contained within the imaging volume and, thus, not included in quantifications. Additionally, 14 non-MFB structures in synaptic contact with the dendrite were reconstructed. Figure 4: Fig. 4a–e: coCATS labeling in combination with functional recordings is representative of experiments in *n* = 6 organotypic slices. Following the axon trajectory with 3D reconstruction was done for *n* = 1 sample, with bouton characteristics extracted from a total of N_analyzed_ = 17 boutons imaged across multiple volumes along the axon trajectory. Reconstructions were performed on two imaging volumes, as seen in Fig. 4d,e. Figure 4f: coCATS images represent raw data from *n* = 5 brain slices obtained from *n* = 2 independent biological specimens with in vivo microinjection into LV and cortex, respectively. They are representative of coCATS in vivo microinjection in *n* = 10 and *n* = 4 animals for LV and cortical microinjection, respectively. Figure 5: Fig. 5a,b: Data are representative of rCATS in *n* = 10 perfusion-fixed specimens. Figure 5c: rCATS/coCATS co-labeling was performed in *n* = 7 brain sections (technical replicates) across *n* = 3 animals with various fluorophore combinations. Figure 5d: Data are representative of coCATS with MAP in *n* = 3 organotypic slices. Figure 5e–i: Whole-section rCATS with proExM was performed in six brain slices across *n* = 4 animals and skeletonization in *n* = 1 dataset. Figure 6: Fig. 6a–c: rCATS was performed on surgery explants from *n* = 8 patients, and the best-preserved specimens were selected for display here and in Supplementary Fig. 24. Figure 6d: rCATS was performed in five slices from autopsy specimens of *n* = 2 individuals. Figure 6e–i: Data are representative of *n* = 3 technical rCATS replicates from *n* = 1 patient with MOGAD. Figure 6j, k: rCATS data are representative of *n* = 2 technical replicates in peripheral nerve biopsy of *n* = 1 patient. Extended Data Fig. 1: Comparison of confocal versus STED performance in coCATS-labeled specimens was performed in *n* = 3 biological specimens in three independent imaging sessions. Imaging of fluorescent beads is representative for typical microscope performance and was acquired in one imaging session. Extended Data Fig. 2: Displayed data are from a single dataset representative of coCATS with in vivo microinjection into the LV performed in *n* = 10 animals. Extended Data Figs. 3 and 4: Tests for pSCR location relative to synaptic markers in Extended Data Figs. 3 and 4 were performed for a total of 10 markers across 17 brain slices from *n* = 6 animals. Extended Data Fig. 5: Reconstruction was performed on *n* = 1 dataset (same as Fig. 3g), including the positively labeled dendrite with spines (n_spines_ = 68), MFBs (n_MFBs_ = 43) with axons and filopodia (n_axons/filopodia_ = 38), and structures in synaptic contact with the main dendrite, not identifiable as MFB-related structures (n_non-MFB_ = 14). Extended Data Fig. 6: coCATS in vivo microinjection into the LV was performed in *n* = 10 biological specimens. Astrocyte 3D reconstruction was performed once. Extended Data Fig. 7: Imaging data are representative and were acquired across eight different brain sections from *n* = 3 individual biological specimens. coCATS labeling of various brain regions was achieved by in vivo microinjection into the LV or cortex, which was performed in *n* = 10 and *n* = 4 biological specimens, respectively. Extended Data Fig. 8: Serial imaging data in **a** are from a single specimen, and data in **b** were acquired across brain slices from *n* = 6 biological specimens. Extended Data Fig. 9: coCATS/rCATS co-labeling was performed in seven brain sections total from *n* = 3 animals with different fluorophore combinations. Extended Data Fig. 10: Imaging data are representative of coCATS labeling in *n* = 5 human cerebral organoids cultured at three different timepoints. Dense manual reconstruction was performed in one dataset. Supplementary Fig. 1: All probes that were used for subsequent routine experiments—that is, STAR RED NHS, ATTO643 NHS and NHS-PEG12-biotin—were tested 3× in organotypic slice cultures from different culture timepoints (*n* = 3 biological specimens). All other probes were tested in *n* = 2 biological specimens, except for AF546 NHS, AF594 NHS and maleimide-PEG11-biotin. AF546 NHS and AF594 NHS were tested only once, as the staining pattern matched the pattern of other NHS-conjugated fluorophores. Maleimide-PEG11-biotin was tested only once, as the result matched the labeling pattern of Atto643 maleimide. Supplementary Fig. 2: Serial whole-brain sectioning and overview imaging of the dye distribution in the brain after LV injection was performed in *n* = 5 animals. Injection of coCATS label into the LV and imaging as described for the various datasets throughout the manuscript were performed in *n* = 10 animals. Supplementary Fig. 3: Training N2V networks in independent N2V runs to obtain *n* = 5 technical replicates for the same volumetric dataset was done in *n* = 1 specimen. Supplementary Fig. 4: The data displayed are representative comparisons of raw versus denoised imaging data as displayed in the main figures and were recorded across *n* = 5 biological specimens. Supplementary Fig. 5: Displayed images show representative examples of automated and proofread pSCR segmentations. coCATS in vivo microinjection into the LV for labeling CA3 stratum lucidum was performed in *n* = 10 biological specimens. Supplementary Fig. 6: Comparison of confocal versus STED performance in coCATS-labeled specimens is representative of imaging in *n* = 3 biological specimens. It is furthermore representative of the improved tissue visualization with *xy*-STED or *z*-STED imaging over diffraction-limited imaging in a number of measurements throughout the manuscript, recorded across multiple biological specimens (see, for example, Figs. 1c, 4f and 5a and Extended Data Figs. 1, 3, 4, 7, 8 and 9). The illustration experiment in **g** was done in *n* = 1 sample and is representative of routinely setting the correction collar to the desired imaging depth in our (STED) imaging experiments. The correction collar was set to 0.17 once and imaged. The other values were set and imaged twice. Supplementary Fig. 7: **a** shows eight reconstructed MFBs representing, together with the 22 reconstructed boutons in Fig. 2b, the total of 30 MFBs quantified in Fig. 2c–g (n_MFB_ = 30, with 10 selected from each of three imaging volumes, recorded across two brain sections (*n* = 2 technical replicates) from one animal (*n* = 1 biological replicate)). Analysis in **b** was performed on the n_MFB_ = 30 reconstructed MFBs. Supplementary Fig. 8: coCATS in vivo microinjection into the LV as used here was performed in *n* = 10 biological specimens. The N2V deep learning model in **a**–**d**,**f**,**i** was trained on *n* = 13 denoised volumetric imaging datasets recorded across four brain sections coming from three animals (*n* = 3 biological replicates). The raw deep learning model in **c**,**d** was trained on the same datasets without denoising. The deep learning model trained on confocal BASSOON (**e**) was trained on *n* = 8 volumetric imaging datasets recorded across three brain slices from two animals. The training data for the DL model based on STED-BASSOON in **e** was size matched to training on confocal BASSOON and consisted of *n* = 9 volumetric imaging datasets recorded across four brain slices from three animals. Supplementary Fig. 9: coCATS labeling in combination with functional recordings and dye filling of various cell types was performed in six organotypic brain sections (*n* = 6 biological specimens). Supplementary Fig. 10: Measurements in **a** were performed in cultures prepared at three different timepoints and comprised 11 control cells recorded across three slices (*n* = 11 cells) and nine cells recorded across four dye-exposed slices (STAR RED-NHS, *n* = 9 cells). Electrophysiological recording during dye incubation (**b**) was performed in *n* = 3 biological specimens. Supplementary Fig. 11: coCATS labeling in combination with functional recordings and dye filling of various cell types was performed in six organotypic brain sections (*n* = 6 biological specimens). The data stem from a single imaging volume (same as Fig. 3g–i). Supplementary Fig. 12: coCATS labeling in combination with functional recordings and dye filling of various cell types was performed in six organotypic brain sections (*n* = 6 biological specimens). All boutons positively labeled here belong to a single cell (same as Fig. 4a–d) and were acquired across multiple imaging volumes along the axon in the same organotypic slice (*n* = 1 biological specimen). Supplementary Fig. 13: Same dataset as in Fig. 4e. coCATS labeling in combination with functional recordings and dye filling of various cell types was performed in six organotypic brain sections (*n* = 6 biological specimens). Supplementary Fig. 14: Tracing was performed in *n* = 10 axons from one imaging volume in *n* = 1 biological specimen. Supplementary Fig. 15: The data displayed were acquired from the same biological specimen. coCATS in vivo microinjection in the cortex was performed in four animals (*n* = 4 biological specimens). Supplementary Fig. 16: coCATS combined with myelin labeling was performed in two brain sections (*n* = 2 technical replicates) from one biological specimen. Supplementary Fig. 17: After initial screening, involving *n* = 3 biological replicates for WGA, this lectin was used for further experiments. The other lectins and HABP were not further pursued after testing in *n* = 1 brain section each. Supplementary Fig. 18: rCATS in combination with sparse *Thy1-eGFP* labeling was performed in *n* = 3 biological specimens. rCATS in combination with fluoromyelin labeling in **d** was performed on four brain slices across *n* = 3 animals. Supplementary Fig. 19: rCATS in perfusion-fixed brain slices was performed in *n* = 10 biological specimens. Exemplary MFB segmentation from rCATS data was performed for two MFBs from one imaging volume. Supplementary Fig. 20: rCATS labeling in perfused brain slices, as seen in **a**, was performed in *n* = 10 biological specimens. rCATS labeling of immersion-fixed half-hemispheres was performed once. The effect of permeabilization conditions on rCATS and antibody labeling depth (**b**–**d**) was tested twice, in *n* = 2 independent biological specimens. Supplementary Fig. 21: coCATS labeling of organotypic brain sections in combination with MAP (**a**,**b**) was performed in *n* = 3 biological specimens. coCATS labeling of organotypic brain sections in combination with proExM (**c**,**d**) was performed in *n* = 3 biological specimens. Supplementary Fig. 22: Test experiments with the various anchoring compounds (**b**) were performed once for each of the two expansion protocols, with and without anchors. Higher labeling intensity upon anchoring with streptavidin acrylamide was confirmed twice—that is, in a total of *n* = 3 biological replicates each for proExM and MAP. Supplementary Fig. 23: Whole coronal brain slice expansion in combination with proExM was performed in six samples (*n* = 6 technical replicates) across *n* = 4 animals. The representative imaging data displayed here were acquired in a single specimen. Supplementary Fig. 24: rCATS imaging in surgery explants was performed in *n* = 8 patients with epilepsy, from whom we selected the samples in Fig. 6a–c and Supplementary Fig. 24 for quality of structural representation. Supplementary Fig. 25: Data are representative of rCATS imaging in *n* = 5 brain sections obtained across *n* = 2 autopsy specimens, of which the displayed sample featured better structural preservation. Comparison with H&E staining was performed once. Supplementary Fig. 26: Imaging was performed for one patient with MOGAD (*n* = 1) on three brain sections (*n* = 3 technical replicates) for the rCATS labeling in combination with immunostaining. Comparison with Luxol fast blue and CD68 staining was performed once. Supplementary Fig. 27: rCATS labeling in combination with immunostaining of a human peripheral nerve biopsy was performed for one patient (*n* = 1, same as in Fig. 6j,k) with *n* = 2 technical replicates (two sections). Comparison with neurofilament H staining (**g**) was performed once.

#### Statistics

Graphs, except for line profile graphs, were created with GraphPad Prism (version 9.0.2). Graphs of line profiles, created with ImageJ/Fiji, were generated with Excel 2016. All statistical tests were performed with GraphPad Prism.

Graphs in Fig. 2c–e and Supplementary Fig. 14c show individual data points (gray circles) as well as mean and s.d. Graphs in Fig. 2f,g and Supplementary Fig. 7b,c show individual data points (gray circles) and linear regression lines with their corresponding R^2^ values. Pearson correlation was performed to test the extent of linear correlation in these datasets and is reported in the form of Pearson correlation coefficient (*r*) with 95% CI and two-tailed *P* value (*P*). Volume distributions in Fig. 3j are displayed as violin plots with medians (lines) and quartiles (dashed lines). Single MFB and single spine connectivities in Fig. 3k,l are displayed as pie charts including percentage of all instances. The graph in Supplementary Fig. 10a shows mean and s.d. of 11 (control) and nine (STAR RED-NHS) cells per current. Unpaired two-tailed *t*-test was used to compare whether the difference between the two experimental groups (control and STAR RED-NHS) for each current was significant (S, *P* < 0.05) or not significant (NS, *P* > 0.05).

### Reporting summary

Further information on research design is available in the Nature Portfolio Reporting Summary linked to this article.

## Online content

Any methods, additional references, Nature Portfolio reporting summaries, source data, extended data, supplementary information, acknowledgements, peer review information; details of author contributions and competing interests; and statements of data and code availability are available at 10.1038/s41587-023-01911-8.

### Supplementary information

Supplementary Information List of reagents, Supplementary Figs. 1–27, Notes 1 and 2 and References.

Reporting Summary

Supplementary Table 1 Labeling and imaging parameters for individual measurements.

Supplementary Video 1 3D rendering of a hippocampal MFB. Volume segmentation (blue) and MFB surface area occupied by pSCRs (white) of the enlarged MFB in Fig. 2b. 3D reconstruction from volumetric, near-isotropically resolved (*z*-STED) coCATS data acquired in the CA3 stratum lucidum of an adult, coCATS in vivo microinjected and perfusion-fixed mouse.

Supplementary Video 2 coCATS volume and MFB segmentations in the CA3 stratum lucidum. Fly-through (*xz* view) of the volume in Fig. 2h, including BASSOON (magenta, confocal, N2V), SHANK2 (turquoise, *z*-STED, N2V) and coCATS (gray, *z*-STED, N2V) as well as 10 segmented MFBs. MFB surface areas occupied by pSCRs are indicated in white. Step size, 50 nm (537 optical sections corresponding to 26.85 µm). For visualization, contrast-limited adaptive histogram equalization (CLAHE, ImageJ) was used to account for differential photobleaching.

Supplementary Video 3 coCATS volume in hippocampal neuropil. Fly-through of coCATS volume in Fig. 3a (*xy* view, *z*-STED, N2V), acquired in the neuropil of an organotypic hippocampal slice. Step size, 50 nm (220 optical sections corresponding to 11 µm).

Supplementary Video 4 Reconstructing the local input field of a CA3 PN from volumetric coCATS data. Fly-through of coCATS imaging volume (gray, *z*-STED, N2V) with intracellular positive labeling of a CA3 PN (yellow, confocal) (*xy* view, 160 optical sections corresponding to 8 µm). 3D renderings of the CA3 PN proximal dendrite (gold) and 57 synaptically connected structures (multi-colored) reconstructed from coCATS data are shown, as displayed in Fig. 3h. Connected structures were identified by the presence of pSCRs (white).

Supplementary Video 5 Reconstruction of the local output structure of a mossy fiber in the DG hilus. Volume rendering of a piece of DG granule cell axon (mossy fiber, orange) with three MFBs and their corresponding post-synaptic structures (green), identified by the presence of pSCRs (white), as displayed in Fig. 4d.

Supplementary Video 6 Reconstruction of the local output structure of a single MFB in the CA3 stratum lucidum. Volume rendering of an MFB (orange), including its axon and filopodial extensions, and nine post-synaptic structures (turquoise/blue) identified by the presence of pSCRs (white), as displayed in Fig. 4e.

Supplementary Video 7 Reconstruction of an astrocyte in the CA3 stratum lucidum. Volume rendering of an astrocyte from the coCATS imaging volume in Extended Data Fig. 6, recorded with STED at near-isotropic resolution. An initial manual segmentation (magenta) was performed based on coCATS data alone. Additionally taking into account specific immunolabeling for glial fibrillary acidic protein, an intermediate filament type expressed in astrocytes, facilitated assigning and segmenting further fine cellular protrusions (violet) based on CATS data.

Supplementary Video 8 coCATS volume in a human cerebral organoid. Fly-through along the *z* direction and volume rendering of a piece of cerebral organoid (29.9 × 22.9 × 8.7 µm^3^; Extended Data Fig. 10), coCATS labeled and imaged at near-isotropic super-resolution by STED microscopy, using contrast limited adaptive histogram equalization (CLAHE) for visualization.

## Data Availability

Source data are available at the Institute of Science and Technology Austria’s data repository at 10.15479/AT:ISTA:13126 (https://research-explorer.ista.ac.at/record/13126).

## Code availability

Code related to this publication is available at https://github.com/danzllab/CATS.
