# Supplementary material for: Imaging brain tissue architecture across millimeter to nanometer scales
Source: Nat Biotechnol. 2023 Aug 31;42(7):1051–64. doi: 10.1038/s41587-023-01911-8 (PMC11252008; doi:10.1038/s41587-023-01911-8)
Supplement: Supplementary file 1 — List of reagents, Supplementary Figs. 1–27, Notes 1 and 2 and References. [file 41587_2023_1911_MOESM1_ESM.pdf]

nature biotechnology

Article https://doi.org/10.1038/s41587-023-01911-8

Imaging brain tissue architecture across millimeter to nanometer scales

In the format provided by the authors and unedited

Table of contents

1 Reagents 2

Chemicals & solutions 2
Probes & antibodies 4

2 Supplementary Figures and Notes 7

Supplementary Fig. 1 7
Supplementary Fig. 2 9
Supplementary Fig. 3 10
Supplementary Fig. 4 12
Supplementary Fig. 5 14
Supplementary Fig. 6 16
Supplementary Fig. 7 18
Supplementary Note 1: Prediction and validation of synapse location 19
Supplementary Fig. 8 23
Supplementary Fig. 9 25
Supplementary Fig. 10 26
Supplementary Fig. 11 27
Supplementary Fig. 12 28
Supplementary Fig. 13 30
Supplementary Fig. 14 31
Supplementary Fig. 15 32
Supplementary Fig. 16 34
Supplementary Fig. 17 35
Supplementary Fig. 18 37
Supplementary Fig.19 39
Supplementary Note 2: Fixation, permeabilization and antigen retrieval protocols for CATS. 41
Supplementary Fig. 20 43
Supplementary Fig. 21 45
Supplementary Fig. 22 46
Supplementary Fig. 23 48
Supplementary Fig. 24 50
Supplementary Fig. 25 51
Supplementary Fig. 26 52
Supplementary Fig. 27 54

3 References 56

1 Reagents

Chemicals & solutions

Table 1: List of chemicals.

Reagent	Abbr.	Source	Identifier
2-Mercaptoethanol (50 mM)	-	ThermoFisher Scientific/ Gibco	31350010
2-(N-morpholino)ethanesulfonic acid	MES	Sigma Aldrich/ Merck	M3671
(3-Aminopropyl)triethoxysilane	-	Sigma Aldrich/ Merck	A3648
3′,3-diaminobenzidine	DAB	Agilent/DAKO	K3468
4-(2-hydroxyethyl)-1-piperazineethanesulfonic acid	HEPES	Sigma Aldrich/ Merck	H3375
4-(2-hydroxyethyl)-1-piperazineethanesulfonic acid 1 M	HEPES 1 M	ThermoFisher Scientific/ Gibco	15630056
4-hydroxy-2,2,6,6-tetramethylpiperidine-	TEMPO	Sigma Aldrich/ Merck	176141
6-((acryloyl)amino)hexanoic acid, succinimidyl ester	AcX	ThermoFisher Scientific	A20770
Accutase	-	Sigma Aldrich/ Merck	A6964
Acrylamide	AA	Sigma Aldrich/ Merck	A9099
Acrylic acid N-hydroxysuccinimide ester	NAS	Sigma Aldrich/ Merck	A8060
Adenosine-5'-triphosphate-disodium salt hydrate	Na_2ATP	Sigma Aldrich/Merck	A3377
Agarose	-	Sigma Aldrich/ Merck	#A9539
Ammonium persulfate	APS	Sigma Aldrich/ Merck	A3678
Antibody diluent	-	Agilent/ Dako	S080983-2
Ascorbic acid	-	Sigma Aldrich/ Merck	A5960
B-27 supplement (50X) serum free	B27	ThermoFisher Scientific/ Gibco	17504044
B-27 supplement (50X) (-vitamin A)	B27 (-vitA)	ThermoFisher Scientific/ Gibco	12587010
Biocytin	-	ThermoFisher Scientific/ Invitrogen	B1592
Bovine serum albumin, albumin fraction V (pH 7.0)	BSA	PanReac AppliChem	A1391,0500
Calcium chloride dihydrate	$CaCl_2$	Sigma Aldrich/ Merck	C3881
Citric acid monohydrate	Citric acid	Fisher Scientific	10345410
Crimson microspheres 40 nm	-	Abberior	NA
D-glucose	-	Sigma Aldrich/ Merck	G8270
Disodium hydrogen phosphate	Na_2HPO_4	Sigma Aldrich/ Merck	S9763
DMEM/F-12, HEPES	DMEM/F-12	ThermoFisher Sientific/ Gibco	11330057
EnVision® Flex+ kit	-	Agilent/DAKO	K800221-2
Ethanol	EtOH	Sigma Aldrich/Merck	32221
Ethylenediaminetetraacetic acid (0.5 M), pH 8.0, RNase-free	EDTA	ThermoFisher Scientific/ Invitrogen	AM9261
Ethylene glycol-bis(2-aminoethylether-N,N,N',N'-tetraacetic acid	EGTA	Sigma Aldrich/Merck	E0396
Fetal bovine serum	FBS	ThermoFisher Scientific	10270106
Fluoromount G™	-	Biomedica	0100-01
Glutamax	-	ThermoFisher Scientific/ Gibco	35050038/ 35050061
Glycerol Bioxtra	-	Sigma Aldrich/ Merck	G6279
Guanidine hydrochloride	HCl	Sigma Aldrich/ Merck	G3272
Guanosine 5'-triphosphate sodium salt hydrate	GTP	Sigma Aldrich/ Merck	G8877
Hank's Balanced Salt Solution (without calcium and magnesium)	HBSS (-/-)	ThermoFisher Scientific/ Gibco	14175053
Hank's Balanced Salt Solution (calcium, magnesium)	HBSS (+/+)	ThermoFisher Scientific/ Gibco	24020091

Heparin sodium salt from porcine intestinal mucosa	Heparin	Sigma Aldrich/ Merck	H3149
Horse serum	HS	ThermoFisher Scientific/ Gibco	26050088
Insulin solution human	Insulin	Sigma Aldrich/ Merck	I9278
Isoflurane	-	Virbac/ Vetflurane	NA
Ketaminol 100 mg/ml	Ketamine	MSD Tiergesundheit	NA
L-Ascorbic acid	Vitamin C	Sigma Aldrich/ Merck	A4544
Magnesium chloride	$MgCl_2$	Sigma Aldrich/ Merck	M8266
Magnesium sulfate	$MgSO_4$	Sigma Aldrich/ Merck	M2643
Corning® Matrigel® Matrix	-	Corning	356235
Mc Coy’s 5A (modified) medium	Mc Coy’s	ThermoFisher Scientific/ Gibco	26600023
Minimum Essentials Medium, Earl’s salts w/o glutamine	MEM	ThermoFisher Scientific/ Gibco	11095080
Minimum Essentials Medium, Non-Essential Amino Acids Solution (100X)	MEM-NEAA	ThermoFisher Scientific/ Gibco	11140050
mTeSR1 medium	-	StemCell Technologies	85850
N-2 Supplement (100x)	N-2	ThermoFisher Scientific/ Gibco	17502048
Neurobasal medium	Neurobasal	ThermoFisher Scientific/ Gibco	21103049
Normal goat serum	NGS	ThermoFisher Scientific/ Invitrogen	31872
Novalgin 0.5 g/ml	Metamizol	Sanofi	NA
N,N-dimethylacrylamide	DMAA	Sigma Aldrich/ Merck	274135
N,N‘ methyleneis-acrylamide	BIS	Sigma Aldrich/ Merck	M7279
N,N,N‘,N‘-tetramethyl-ethyelenediamine	TEMED	Sigma Aldrich/ Merck	T9281
OleoVital eye ointment (Dexpanthenol 2%)	-	Fresenius Kabi	NA
Paraformaldehyde	PFA	Sigma Aldrich/ Merck	158127
Formaldehyde EM grade	PFA EM grade	TAAB Laboratory and Microscopy	P026
Penicillin-Streptomycin (10,000 U/ml)	PenStrep	ThermoFisher Scientific/ Gibco	15140122
Potassium chloride	KCl	Sigma Aldrich/ Merck	P5405
Potassium gluconate	K-gluconate	Sigma Aldrich/ Merck	G4500
Potassium persulfate	KPS	Sigma Aldrich/ Merck	379824
Proteinase K	-	New England Biolabs (NEB)	P8107S
Sodium azide	NaN_3	Sigma Aldrich/ Merck	71289
Sodium bicarbonate	$NaHCO_3$	Sigma Aldrich/ Merck	S5761
Sodium acrylate	SA	Sigma Aldrich/ Merck	408220
Sodium chloride BioXtra	NaCl	Sigma Aldrich/ Merck	S7653
Sodium chloride (human tissue)	NaCl	Braun	3570160
Sodium dodecyl sulfate	SDS		436143
Sodium hydrogencarbonate	$NaHCO_3$	Millipore	106329
Sodium hydroxide	NaOH	Sigma Aldrick/ Merck	S5881
Sodium phosphate monobasic monohydrate	$NaHPO_4*H_2O$	Sigma Aldrick/ Merck	71504-MM
Sucrose/ D-(+)saccharose	Sucrose	Sigma Aldrick/ Merck	84097
Triethanolamine	-	Sigma Aldrich/ Merck	122262
Tris 1 M, pH 8.0, RNase-free	-	ThermoFisher Scientific/ Invitrogen	AM9856
Triton X-100	TX	Sigma Aldrich/ Merck	X100
Urea	-	Sigma Aldrich/ Merck	U5378
Xylenes, 98.5%	Xylene	Sigma Aldrich/ Merck	247642
Xylasol 20 mg/ml	Xylazine	Livisto	NA
Y-27632 dihydrochloride	Y-27632	StemCell Technologies	72302

Abbr.=abbreviation

Table 2: List of solutions.

Solution	Abbr.	pH	Component	Molarity (mM)
0.2 M phosphate buffer	0.2 M PB	7.4	Sodium phosphate dibasic heptahydrate ($Na_2HPO_4*7H_2O$)	150.82
			Sodium phosphate monobasic monohydrate ($NaH_2PO_4*H_2O$)	49.18
10X phosphate buffered saline	10X PBS	7.4	Sodium Phosphate dibasic (Na_2HPO_4)	100.00
			Potassium Phosphate Monobasic (KH_2PO_4)	19.84
			Potassium chloride (KCl)	26.83
			Sodium chloride (NaCl)	1368.90
1X phosphate buffered saline	1X PBS	7.4	Sodium Phosphate dibasic (Na_2HPO_4)	10.00
			Potassium Phosphate Monobasic (KH_2PO_4)	1.98
			Potassium chloride (KCl)	2.68
			Sodium chloride (NaCl)	136.89

Probes & antibodies

Table 3: List of probes and antibodies.

Reagent	Abbr.	Working c.	Source	Identifier
4'6-diamidino-2-phenylindole	DAPI	2.5-5 µg/ml	Sigma Aldrich/ Merck	D9542
Alexa Fluor 488-avidin	AF488-avidin	20 µg/ml	ThermoFisher Scientific/ Invitrogen	A21370
Alexa Fluor 488 donkey anti-rabbit IgG (H+L)	AF488 anti-rb	5 µg/ml	Jackson ImmunoResearch	711-545-152
Alexa Fluor 488 donkey anti-guinea pig IgG (H+L)	AF488 anti-gp	1-2 µg/ml	Jackson ImmunoResearch	706-545-148
Alexa Fluor 488 goat anti-mouse IgG (H+L)	AF488 anti-m	6-20 µg/ml	Thermo Fisher Scientific/ Invitrogen	A11001
Alexa Fluor plus 488 goat anti-rabbit IgG (H+L)	AF488 + anti-rb	4 µg/ml	Thermo Fisher Scientific/ Invitrogen	A32731
Alexa Fluor 488-N-hydroxysuccinimide	AF488-NHS	40-50 µM	Jena Bioscience	APC-002-1
Alexa Fluor 546 goat anti-guinea pig IgG (H+L)	AF546 anti-gp	5 µg/ml	Thermo Fisher Scientific/ Invitrogen	A11076
Alexa Fluor 546-N-hydroxysuccinimide	AF546-NHS	40-50 µM	Thermo Fisher Scientific/ Invitrogen	A20002
Alexa Fluor 594 goat anti-guinea pig IgG (H+L)	AF594 anti-gp	4-8 µg/ml	Thermo Fisher Scientific/ Invitrogen	A11076
Alexa Fluor 594 goat anti-mouse IgG (H+L)	AF594 anti-m	4-8 µg/ml	Thermo Fisher Scientific/ Invitrogen	A11005
Alexa Fluor 594 goat anti-rabbit IgG (H+L)	AF594 anti-rb	8 µg/ml	ThermoFisher Scientific/ Invitrogen	A11037
Alexa Fluor 594-N-hydroxysuccinimide	AF594-NHS	40-50 µM	Jena Bioscience	APC-004-1
Alexa Fluor 594-streptavidin	-	4 µg/ml	Thermo Fisher Scientific/ Invitrogen	S32356
Alexa Fluor 647 donkey anti-sheep IgG (H+L)	AF 647 anti-sh	4 µg/ml	Thermo Fisher Scientific/ Invitrogen	A21448
Anti-Bassoon monoclonal mouse (SAP7F407)	Anti-BASSOON, m	2-4 µg/ml	Abcam	ab82958, clone: SAP7F407
Anti-CD68 monoclonal, mouse	Anti-CD68, m	1:100 dilution	DAKO	M0814, clone: KP1

Anti-Gephyrin monoclonal, mouse	Anti-GEPHYRIN, m	2 µg/ml	Synaptic Systems	147111, clone: 3B11
Anti-glial fibrillary acidic protein monoclonal, mouse	Anti-GFAP, m	2 µg/ml	Synaptic Systems	173011, clone: 134B1
Anti-green fluorescent protein monoclonal, mouse	Anti-GFP, m	4 µg/ml	ThermoFisher Scientific/ Invitrogen	A11120, clone: 3E6
Anti-green fluorescent protein polyclonal, rabbit	Anti-GFP, rb	5 µg/ml	ThermoFisher Scientific/ Invitrogen	A11122
Anti-Homer1 polyclonal, rabbit	Anti-HOMER1, rb	2 µg/ml	Synaptic Systems	160003
Anti-ionized calcium binding adaptor molecule 1 polyclonal, rabbit	Anti-IBA1, rb	1-2 µg/ml	Wako Chemicals/ Fujifilm	019-19741
Anti-microtubule associated protein 2 polyclonal, guinea pig	Anti-MAP2, gp	1 µg/ml	Synaptic Systems	188004
Anti-Munc13-1 polyclonal, guinea pig	Anti MUNC13-1, gp	2 µg/ml	Synaptic Systems	126104
Anti-myelin basic protein polyclonal, mouse	Anti-MBP, m	NA (1:200 dilution)	Millipore	AB5864
Anti-myelin oligodendrocyte glycoprotein, monoclonal, mouse	Anti-MOG, m	2 µg/ml	Atlas Antibodies	AMAb91067, clone: CL2858
Anti-NEUN polyclonal, guinea pig	Anti-NEUN, gp	2 µg/ml	Synaptic Systems	266004
Anti-Neurofilament H, phosphorylated (SMI31), monoclonal, mouse	Anti-Neurofilament H, m	1:1000-1:5000 dilution	BioLegend, Covance	801601, SMI31P, clone: SMI31
Anti-Shank2 polyclonal, guinea pig	Anti-SHANK2, gp	2-4 µg/ml	Synaptic Systems	162204
Anti-Synapsin1/2 polyclonal, guinea pig	Anti-SYP1/2, gp	2 µg/ml	Synaptic Systems	106004
Anti-Synaptobrevin2 monoclonal, mouse	Anti-VAMP2, m	2 µg/ml	Synaptic Systems	104211, clone: 69.1
Anti-Synaptophysin 1 monoclonal, mouse	Anti-SYPH1, m	2 µg/ml	Synaptic Systems	101011, clone: 7.2
Anti-Synaptophysin 1 polyclonal, guinea pig	Anti-SYPH1, gp	2 µg/ml	Synaptic Systems	101004
Anti-vesicular gamma-aminobutyric acid transporter polyclonal, rabbit	Anti-VGAT, rb	3 µg/ml	Synaptic Systems	131003
Anti-vesicular glutamate transporter 1 polyclonal, rabbit	Anti-VGLUT 1, rb	10 µg/ml	Synaptic Systems	135302
ATTO643-azide	-	20-33 µM	Atto-Tec	AD643-101
ATTO643-biotin	-	4-8 µg/ml	Atto-Tec	AD 643-71
ATTO643-maleimide	-	10 µM	Atto-Tec	AD 643-41
ATTO643-N-hydroxysuccinimidyl ester	ATTO643-NHS	40-50 µM	Atto-Tec	AD 643-31
ATTO643-streptavidin	-	4 µg/ml	Atto-Tec	AD 643-61
Concanavalin A	-	5 µg/ml	Vectorlabs/ Szabo Scandic	VECB-1005
Dibenzocyclooctyne-PEG4-N-hydroxysuccinimidyl ester	NHS-PEG_4-DBCO	50 µM	Sigma Aldrich/ Merck	764019
Endo-bicyclononyne-PEG12-N-hydroxysuccinimidyl ester	NHS-PEG_{12}-BCN	250 µM	Tebu-bio	BP-23766

FluoroMyelin™ Green Fluorescent Myelin Stain	FluoroMyelin	1:300 (concentration NA)	ThermoFisher Scientific/ Invitrogen	F34651
Hyaluronic acid binding protein, biotinylated	HABP-biotin	10 µg/ml	Merck	385911
Jacalin biotin	-	5 µg/ml	Vectorlabs/ Szabo Scandic	VECB-1155
Lucifer Yellow CH lithium salt	Lucifer Yellow	0.2% (w/v)	Sigma Aldrich/ Merck	L0259
Lycopersicon esculentum (tomato) lectin DyLight 594	LEL DyLight 594	2.5 µg/ml	ThermoFisher Scientific	L32471
Maleimide-PEG11-biotin	Maleimide-PEG_{11}-biotin	200 µM	ThermoFisher Scientific	21911
N-hydroxysuccinimidyl ester-PEG12 – dibenzylcyclooctyne	NHS-PEG_{12}-DBCO	200 µM	Tebu-bio	BP-24149
N-hydroxysuccinimidyl ester –PEG12-biotin, EZ-Link™	NHS-PEG_{12}-biotin	250-1000 µM	ThermoFisher Scientific	A35389
Pentafluorophenyl ester-dPEG36-biotin	PFP-PEG_{36}-biotin	250 µM	Broadpharm	BP-24318
Phaseolus vulgaris erythro-agglutinin biotin	PHA-E biotin	5 µg/ml	Vectorlabs/ Szabo Scandic	VECB-1125
Phaseolus vulgaris leuco-agglutinin biotin	PHA-L biotin	5 µg/ml	Vectorlabs/ Szabo Scandic	VECB-1115
STAR 580-azide	-	10-20 µM	Abberior	ST580-004
STAR 580 goat anti-mouse IgG	STAR 580 anti-m	10-15 µg/ml	Abberior	ST580-1001
STAR 580 goat anti-rabbit IgG	STAR 580 anti-rb	10-15 µg/ml	Abberior	ST580-1002
STAR 635P-neutravidin	-	5-6 µg/ml	Abberior	ST635P-0121
STAR RED-N-hydroxysuccinimidyl ester	STAR RED-NHS	40-50 µM	Abberior	STRED-0002
STAR RED-streptavidin	-	3-4 µg/ml	Abberior	STRED-0120
Streptavidin-acrylamide	Strept-acrylamide	4-20 µg/ml	ThermoFisher Scientific	S21379
Sulfo-dibenzylcyclooctyne-N-hydroxysuccinimidyl ester	NHS-sulfo-DBCO	200 µM	Jena Bioscience	CLK-A124-10
Sulfo- N-hydroxysuccinimidyl ester-biotin, long chain, EZ Link™	NHS-sulfo-biotin	1000 µM	ThermoFisher Scientific	21338
Tetrafluorophenyl ester-dPEG12-biotin	TFP-PEG_{12}-biotin	250 µM	Sigma Aldrich/ Merck	QBD10008
Vicia villosa lectin biotin	VVL biotin	5 µg/ml	Vectorlabs/ Szabo Scandic	VECB-1235
Wheat germ agglutinin Alexa Fluor 594	WGA AF594	5 µg/ml	ThermoFisher Scientific	W11262
Wheat germ agglutinin, biotinylated	WGA biotin	15-20 µg/ml	Vectorlabs/ Szabo Scandic	VECB-1025
Wheat germ agglutinin CF633	WGA CF633	5-10 µg/ml	Biotium	29024-1
Wisteria floribunda lectin biotinylated	WFL biotin	8 µg/ml	Vectorlabs/ Szabo Scandic	VECB-1355

c.=concentration

2 Supplementary Figures and Notes

Supplementary Fig. 1

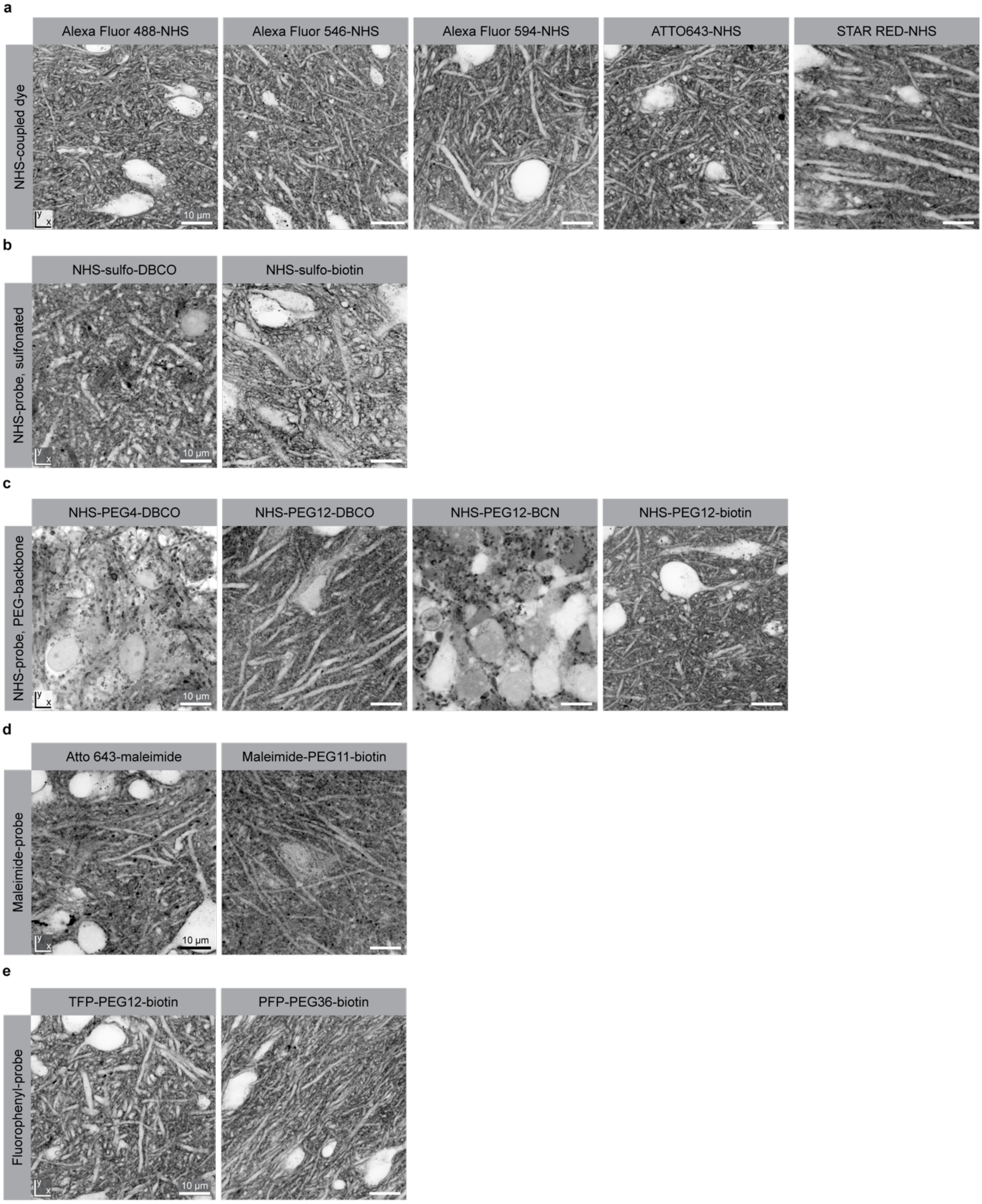

Supplementary Fig. 1| Screening for coCATS labeling probes. *Rationale:* Screening was performed in organotypic hippocampal brain slices (15-30 DIV) to identify compounds for coCATS that are i) compatible with live labeling, ii) provide high extra- to intracellular contrast, iii) mediate covalent attachment to molecules in the extracellular space and on cell surfaces, iv) have sufficient tissue penetration, v) achieve homogeneous/high-density labeling and vi) are compatible with downstream super-resolution imaging, specifically STED and expansion microscopy. For covalent attachment, amine-reactive groups (N-

Hydroxysuccinimidine (NHS), tetrafluorophenyl (TFP), and perfluorophenyl (PFP)) and sulfhydryl-reactive maleimide were tested. Readout labels comprised fluorescent dyes or moieties for downstream targeting with fluorescent readout probes (biotin, click chemistry). Directly dye labeled samples were imaged without permeabilization to avoid additional processing steps that may affect structural preservation. For biotin and click readout, the sample was permeabilized. Biotin readout was particularly useful for experiments involving expansion microscopy, as post-expansion fluorophore addition avoids damage to fluorophores by hydrogel radical chemistry or denaturation steps and provides signal amplification. Samples were live incubated with coCATS labels, followed by immersion-fixation, and imaging in confocal mode. **a,** Hydrophilic, negatively charged, NHS-coupled dyes yielded high extra- to intracellular contrast and homogeneous labeling. **b,** Sulfo-groups increased hydrophilicity and extra- to intracellular contrast for readout moieties that do not provide membrane impermeability by themselves. Dibenzocyclooctyne (DBCO) is a compound for click-chemistry mediated readout. Its NHS ester derivative did not produce satisfactory results, as DBCO is lipophilic and led to aggregate formation in tissue. In contrast, biotin, with subsequent readout via streptavidin, proved to be an excellent readout moiety. **c,** A polyethyleneglycol (PEG) backbone similarly increases hydrophilicity. We observed that a chain length of 11-12 PEG molecules, but not 4 PEG molecules, was sufficient for mediating high extra- to intracellular contrast. Biotin was preferable over DBCO or another click-chemistry compound, bicyclononyne (BCN), also in this case, as it did not produce aggregates in the tissue. **d,** Maleimide compounds covalently react with sulfhydryl groups. The compounds tested here yielded pronounced extracellular labeling but produced a less homogeneous and more granular staining pattern compared to amine reactive compounds. **e,** TFP and PFP are amine reactive compounds that are more resistant to hydrolysis than NHS esters. They produced high quality labeling. The long PEG chain in PFP-PEG_{36}-biotin was not necessary for ensuring extra- to intracellular contrast and merely increased probe size. All scale bars: 10 µm. All probes which were used for subsequent routine experiments, i.e. STAR RED NHS, ATTO643 NHS and NHS-PEG12-biotin, were tested three times in organotypic slice cultures from different culture time points (n=3 biological specimens). All other probes were tested in n=2 biological specimens, except for AF546 NHS, AF594 NHS and maleimide-PEG11-biotin. AF546 NHS and AF594 NHS were tested only once, as the staining pattern matched the pattern of other NHS-conjugated fluorophores. Maleimide-PEG11-biotin was tested only once, as the result matched the labeling pattern of Atto643 maleimide.

Supplementary Fig. 2

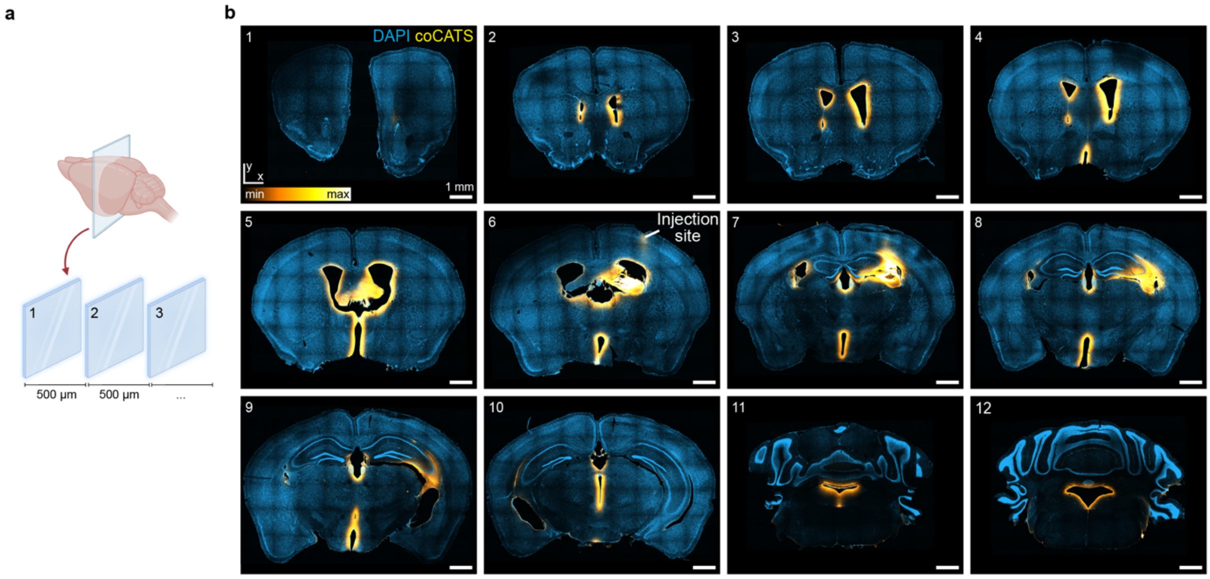

Supplementary Fig. 2| coCATS labeling pattern after in vivo microinjection into the lateral ventricle. coCATS labeling solution was delivered by in vivo microinjection into the left lateral ventricle of an adult anesthetized mouse, followed by a 40 min incubation and perfusion-fixation. **a,** Schematic of serial sectioning scheme of the brain after perfusion-fixation. The entire brain was sectioned into 50 µm thick coronal slices. Every 10th slice, spaced 500 µm apart, was used for overview imaging in panel b. **b,** Tile scans of coronal sections, counter-stained with DAPI (blue), acquired with a spinning disc confocal microscope. Intensity lookup table for CATS (yellow) is not inverted. (1) No staining is visible in the anterior sections. (2,3) Labeling commences in both hemispheres around the anterior region of the lateral ventricle where parts of the caudate-putamen and lateral septal nucleus line the lateral ventricle. (4) By diffusion into the third ventricle, tissue adjacent to it, including in the hypothalamus, is labeled. (5) Further labeling of parts of the striatum, thalamus and hypothalamus, as well as fiber tracts, is visible around the lateral and third ventricles. (6-9) The injection site is visible close to the cortical surface. Strong labeling of the CA3 can be seen throughout the majority of the hippocampus. (10) Labeling of tissue around the third ventricle, including dorsal hypothalamus. (11,12) Diffusion into fourth ventricle leads to staining of parts of the medulla, pons, and a portion of the central lobule of the cerebellar cortex. Serial whole-brain sectioning and overview imaging of the dye-distribution in the brain after lateral ventricle injection was performed in n=5 animals. Injection of coCATS label into the lateral ventricle and imaging as described for the various datasets throughout the manuscript were performed in n=10 animals.

Supplementary Fig. 3

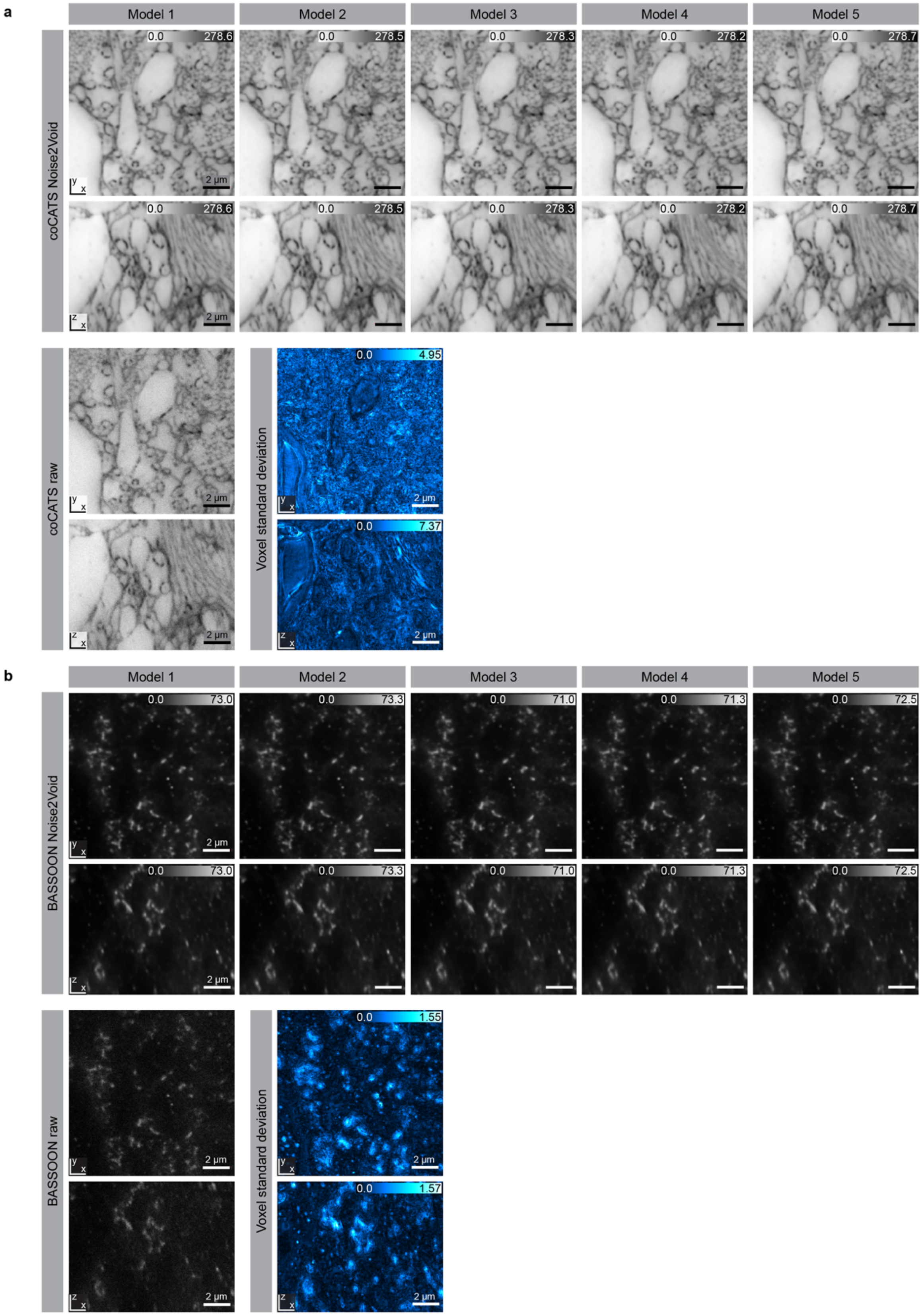

Supplementary Fig. 3| Validation of Noise2Void (N2V) network prediction for denoising CATS and immunostaining data on the same dataset. Single *xy*- and *xz*-planes of **a,**

coCATS labeling and **b,** super-resolved BASSOON immunostaining after in vivo microinjection and perfusion-fixation of an adult mouse. Volumetric imaging data was acquired with a STED microscope, using a *z*-STED pattern for resolution improvement. Figure panels show data denoised with 5 independent N2V network models, raw data, and voxel-wise standard deviation (s.d.) of the 5 independent N2V models. Voxel-wise s.d. reflects the s.d. from the mean of intensity at every voxel across the 5 N2V network models. Highest disagreement is seen in the coCATS channel for areas with low intensity, such as cell bodies, and for BASSOON at the border of immunostaining signals. Note the high degree of similarity of the different N2V outcomes both for the CATS and the immunostaining channels, showing consistency of denoising outcome. This is also reflected by the overall low standard deviation between the models. Scale bars: 2 µm. Training N2V networks in independent N2V runs to obtain n=5 technical replicates for the same volumetric dataset was done in n=1 specimen.

Supplementary Fig. 4

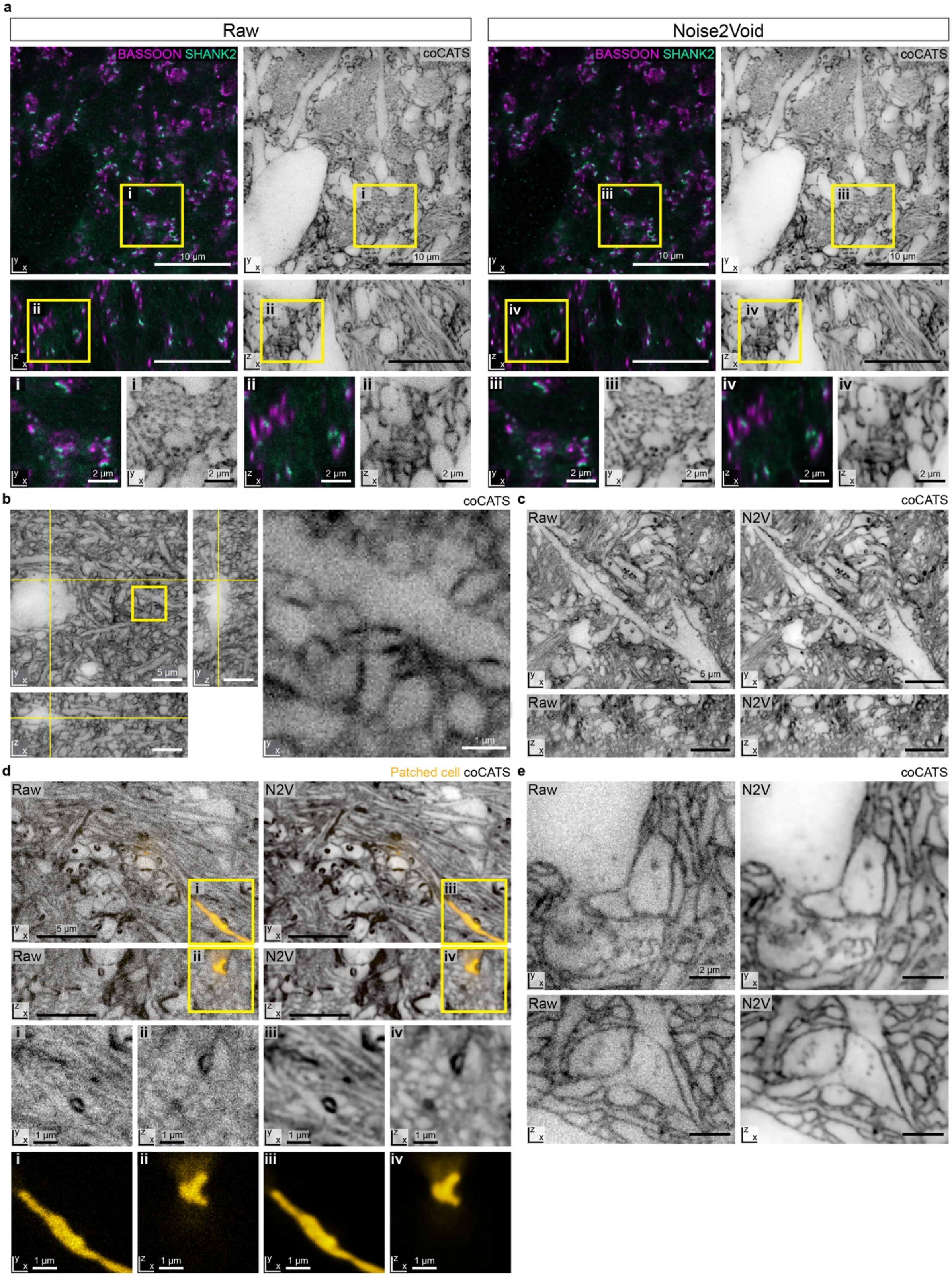

Supplementary Fig. 4| Comparison of raw vs. Noise2Void (N2V)-denoised imaging data. **a,** Single *xy*- and *xz*-sections, as well as zoomed views (i-iv) from one of the three volumetric imaging datasets used for MFB- and pSCR-segmentation in Fig. 2. Raw (*left*) and N2V-

denoised (*right*) data of BASSOON (magenta, confocal), SHANK2 (turquoise, *z*-STED) and coCATS (gray, *z*-STED). Denoising outcome is representative of the 3 imaging volumes analyzed in Fig. 2. **b,** coCATS raw data (*z*-STED) corresponding to the dataset in Fig. 3a,b, showing the same *xy*-, *xz*-sections and zoomed view of the boxed region. **c,** Raw (*left*) and N2V-denoised (*right*) single *xy*- and *xz*-sections of the volumetric coCATS imaging dataset (*z*-STED) displayed in Fig. 3g, used for reconstruction of the input field of a CA3 pyramidal neuron proximal dendrite. **d,** Single *xy*-and *xz*-sections with zoomed views of the boxed regions (i-iv), corresponding to one of the volumetric imaging datasets used for the characterization of a DG granule cell output field (Fig. 4 c,d), displaying coCATS (gray, *z*-STED) and intracellular label (yellow, *z*-STED) **e,** Single *xy*- and *xz*-sections of the volumetric imaging dataset of a human cerebral organoid (coCATS, *z*-STED) displayed in Extended Data Fig. 10 without (*left*) and with (*right*) N2V. The data displayed are representative comparisons of raw vs. denoised imaging data as displayed in the main figures and were recorded across *n*=5 biological specimens.

Supplementary Fig. 5

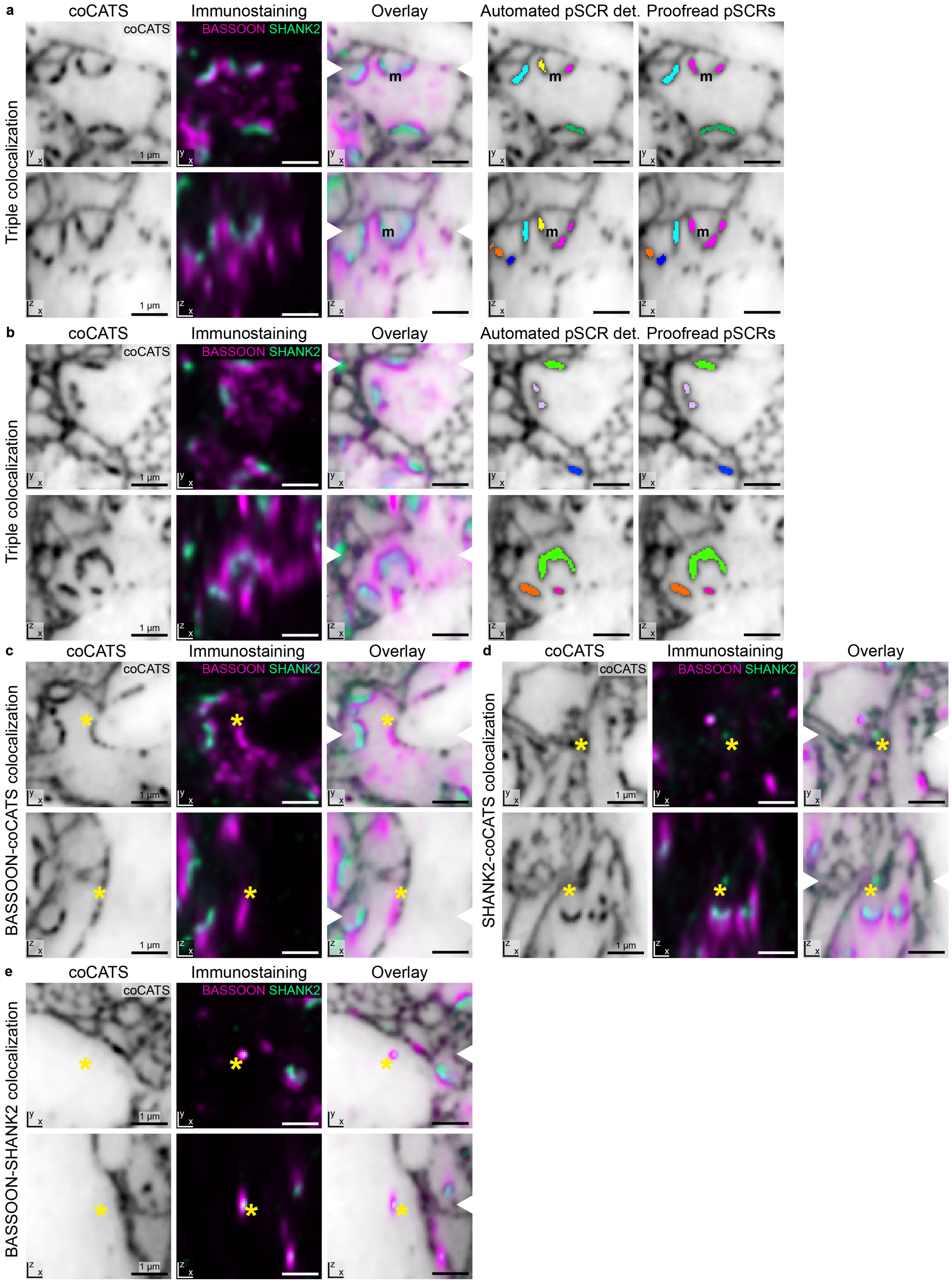

Supplementary Fig. 5| Specificity of automated synapse detection. Examples of colocalization types between BASSOON (magenta, confocal), SHANK2 (turquoise, *z*-STED), and high intensity coCATS features (gray, *z*-STED) taken from the 3 imaging volumes used

for reconstruction and quantification of single-MFB properties in Fig. 2. Single *xy*- and *xz*-sections of coCATS, immunostaining, and overlay, with all channels denoised by N2V. White arrowheads in the overlays indicate positions of the orthogonal views. **a,b,** Orthogonal views for two examples with various instances of BASSOON, high intensity coCATS, and SHANK2 triple colocalization, resulting in pSCR detection (det.) by the automated pipeline. pSCR instance segmentations are displayed multi-color and shown as raw output of the automated segmentation and after manual proofreading of the 3D data. m: example of segments that were merged during proofreading. Scale bars: 1 µm. **c,** Example of colocalization (asterisk) of BASSOON and coCATS feature without SHANK2 staining present, likely corresponding to an inhibitory synapse. **d,** Example of colocalization (asterisk) of SHANK2 and coCATS feature, without BASSOON staining present. Note that SHANK2 signal is weak compared to local surrounding, hinting towards non-specific antibody binding close to a region of high intensity in the coCATS channel. **e,** Example of colocalization (asterisk) of BASSOON and SHANK2 in a region without high intensity coCATS feature. Here, BASSOON and SHANK2 are closely associated and the signal shapes approximate the microscope point spread functions for confocal and STED. This is thus likely a result of non-specific binding of antibodies. In the *xz*-view, the difference in resolution between STED (green) and confocal (magenta) is particularly evident. Cases of double detection, as displayed in c-e, are not classified as synapses by our pipeline for automated pSCR segmentation.
Here, automated pSCR detection involved global thresholding of coCATS data in close spatial proximity to synaptic molecules after denoising with N2V, as in Fig. 2a. Note that for the quantifications of automated pSCR detection in Supplementary Fig. 8, a refined local thresholding scheme of CATS data was applied, involving additional smoothing/background subtraction and grayscale erosion steps before thresholding.
Displayed images show representative examples of automated and proofread pSCR segmentations. coCATS in vivo microinjection into the lateral ventricle for labeling CA3 stratum lucidum was performed in n=10 biological specimens.

Supplementary Fig. 6

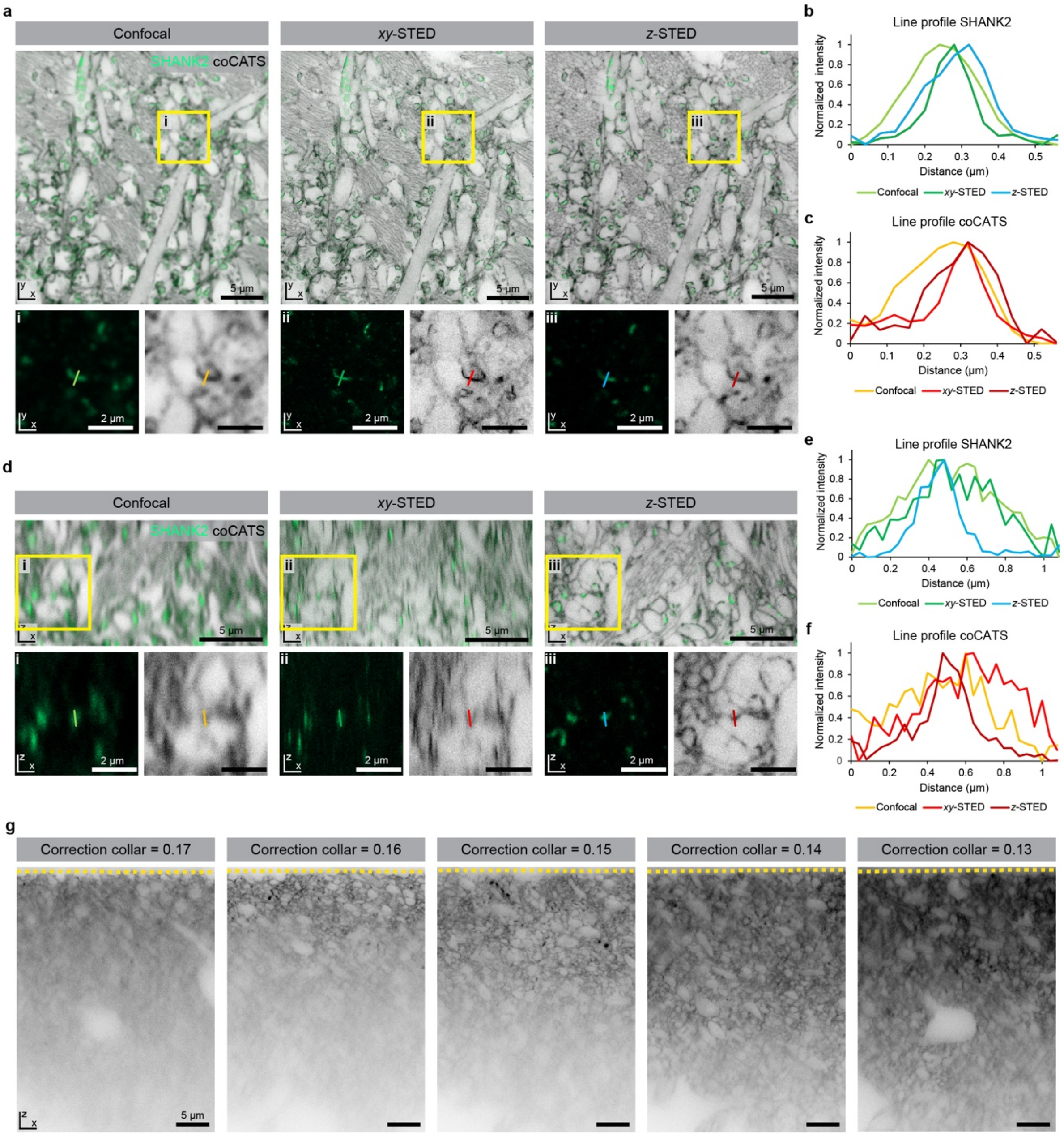

Supplementary Fig. 6| Characterization of STED performance for visualization of tissue architecture and molecule location. a, Single sections in the focal plane (*xy*-view) of confocal, *xy*-STED, and *z*-STED images for coCATS (gray, STAR RED-NHS, in vivo microinjection into the lateral ventricle) and immunolabeling for SHANK2 (green, AF594) of the same region in the hippocampal CA3 stratum lucidum. Bottom panels are magnified views as indicated by the boxes for the immunolabeling and the CATS channels. Resolution is improved both with *xy*-STED and with *z*-STED, with a higher lateral resolution gain by *xy*-STED. **b,c,** Line profiles as indicated in the bottom panels in a. **d,** Axial sections for confocal, *xy*-STED, and *z*-STED in the same specimen. While for *xy*-STED axial resolution is unchanged from confocal imaging, *z*-STED substantially improves axial resolution. **e,f,** Corresponding line profiles as indicated in d. Comparison of confocal vs. STED performance in coCATS-labeled specimens is representative of imaging in n=3 biological specimens. It is furthermore representative of the improved tissue visualization with *xy*- or *z*-STED imaging over

diffraction-limited imaging in a number of measurements throughout the manuscript, recorded across multiple biological specimens (see e.g. Fig. 1c, Fig. 4f, Fig. 5a, Extended Data Fig. 1,3,4,7,8,9). **g,** Imaging in *z*-STED mode as a function of the objective lens' correction collar setting. As the *z*-STED pattern is highly sensitive to spherical aberration from refractive index mismatch, optimum imaging performance is typically achieved over an axial range of ~10 µm in the tissue (see Extended Data Fig. 1d). By varying the correction collar setting, the region of refractive index compensation can be shifted deeper into the tissue. Due to aberrations and scattering, the practical limit with standard sample preparation and imaging procedures (i.e. without dedicated aberration and scattering reduction by e.g. adaptive optics or sample clearing) for high imaging performance is a few tens of micrometers, typically around ~50 µm. The illustration experiment in panel g was done in $n=1$ sample and is representative of routinely setting the correction collar to the desired imaging depth in our (STED) imaging experiments. The correction collar was set to 0.17 once and imaged. The other values were set and imaged twice.

Supplementary Fig. 7

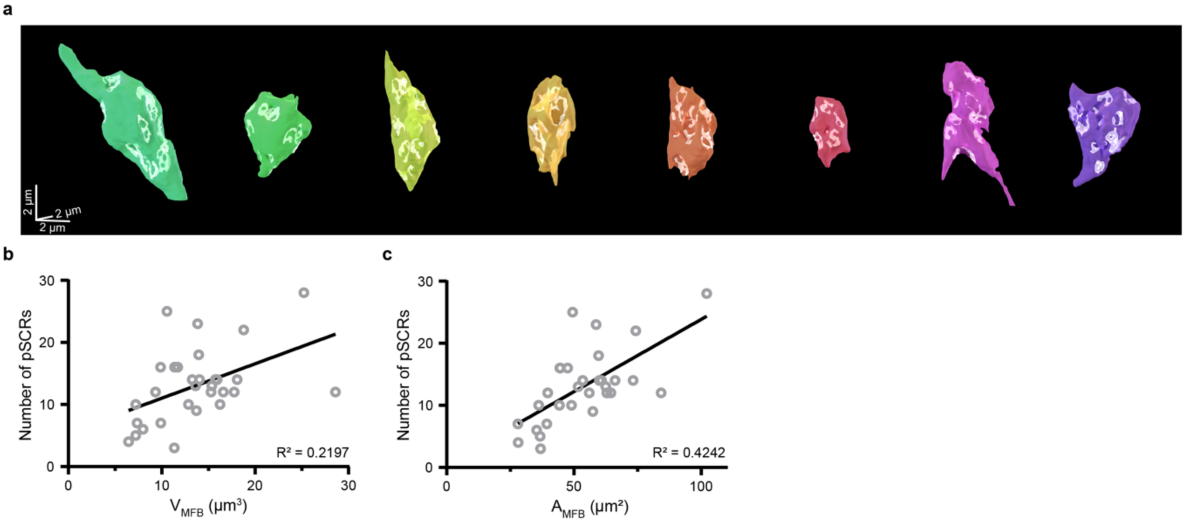

Suppl. Fig. 7| Additional reconstructed MFBs and quantifications. a, Eight additional reconstructed MFBs with pSCRs (white shaded areas) representing together with the 22 reconstructed boutons in Fig. 2b the total of 30 MFBs quantified in Fig. 2c-g (n_{MFB}=30, with 10 selected from each of 3 imaging volumes, recorded across two brain sections (*n*=2 technical replicates) from one animal (*n*=1 biological replicate)). **b,c,** Number of pSCRs associated with individual boutons as a function of MFB volume (V_{MFB}) and MFB surface area (A_{MFB}), including linear regression (V_{MFB}: Pearson correlation coefficient r=0.4687, CI: 0.131-0.709, p=0.009; A_{MFB}, r=0.6513, CI: 0.380-0.819, p<0.0001). Data points represent the individual 30 MFBs in Fig. 2b-g and panel a. Pearson correlation was performed to test the extent of linear correlation in these datasets and is reported in form of Pearson correlation coefficient (r) with 95% confidence intervals (CI), and two-tailed p-value (p). The correlation of MFB volume or MFB surface area with the number of associated pSCRs is lower than with the pSCR area per bouton ($A_{pSCR/MFB}$, Fig. 2f,g), indicating that the number of transmission sites may be less stringently controlled than the overall area of an MFB devoted to synaptic transmission. Note, however, that any merges of adjacent pSCRs due to finite resolution would be directly reflected in pSCR count but less so in pSCR covered surface area.

Supplementary Note 1: Prediction and validation of synapse location.

We employed two types of automated segmentation strategies for detecting and quantifying putative synaptic cleft regions (pSCRs) from coCATS data, one guided by specific molecular labeling and the other guided by deep-learning based prediction of molecule location (for technical details see Methods section). For analysis of biological structures, including pSCR quantification and identification of synaptically connected partners in **Fig. 2b-h,3,4** and **Supplementary Fig. 5,7,11,12,13,19**, we additionally applied manual proofreading of pSCRs in all cases. Accordingly, displayed pSCR segments and analyses were ultimately based on human annotation (unless otherwise noted), using the automated segmentations as guide, which reduced time requirement for human input.

The immunolabeling guided approach for pSCR detection relied on colocalization as defined by partial spatial overlap between segments derived from thresholding of BASSOON immunolabeling, SHANK2 immunolabeling, and CATS (**Figure 2a**). The CATS labeling pattern at pSCRs was corroborated by STED imaging in relation to a total of 10 synaptic markers (**Extended Data Fig. 3,4**). For automated segmentation of CATS features followed by manual proofreading, we used simple global thresholding in regions of CATS data cropped around SHANK2 molecular signals and corrected segments and their shapes manually by inspecting both the immunolabeling STED channels and local tissue context in 3D CATS data (**Supplementary Fig. 5**). Where available, structural segmentations of mossy fiber boutons and synaptic partners displayed in 3 orthogonal planes were taken into account during pSCR proofreading.

For guiding pSCR detection by deep-learning based prediction of molecule location (**Fig. 2i-k,3,4**), we used an image translation U-net trained on CATS and immunolabeling for the synaptic marker BASSOON, both super-resolved with 3D STED microscopy. For training, we used the immunolabeling data directly after a smoothing/background subtraction step without any human intervention, i.e. no further attempt was undertaken to delete unspecific staining from the immunolabeling channel. For volumetric CATS data but not for the immunolabeling channel, we applied denoising with N2V before training.

We first tested the quality of predicted molecule location in a voxel-based manner, calculating the Pearson correlation coefficient ($r=0.818$) between the smoothed/background subtracted BASSOON STED and predicted BASSOON data for the volume in **Supplementary Fig. 8a**, which was not part of the training.

We then used the predicted synaptic molecule locations to distinguish high-intensity CATS features at pSCRs from other high-intensity CATS features not associated with synaptic transmission sites. Similarly as in the immunolabeling based pipeline described above, we applied global thresholding of CATS data in regions around predicted BASSOON location. Final displayed and analyzed pSCR segments were then generated by manual proofreading of the automatically generated segments. For this, the structural CATS data, predicted molecular signal, and, where available, cellular segmentations were inspected in 3 orthogonal planes and automatically generated segments were manually corrected.

We then applied several object-based validation steps to test for the quality of fully automated pSCR segmentations based on this pipeline, i.e. without applying manual proofreading. For this, we slightly refined the thresholding of CATS data to automatically generate segments that

were more similar to the segments generated by a human annotator. We first applied a similar smoothing/background subtraction step as we did for immunolabelled BASSOON also to the CATS data. We then applied a grayscale erosion algorithm to account for the blurring effect introduced by the N2V and background subtraction steps. We now performed global thresholding of the entire preprocessed imaging volume and afterwards selected those segments that were in close vicinity to a predicted BASSOON location. Finally, we removed all segments smaller than 6 voxels.

In a first test (**Fig. 2j,k, Supplementary Fig. 8a-b**), using a dataset that was not part of the training, we compared pSCR segments generated in this way using predicted BASSOON ($pSCR_{prediction}$) vs. pSCR segments generated using immunolabelled BASSOON imaged by STED microscopy ($pSCR_{immuno}$) in the same dataset. Segments were thus created in both cases from the coCATS data and no manual proofreading was applied. With this, we thus evaluated to what extent immunolabeling can be replaced by a deep-learning based prediction of synaptic molecule location in order to guide pSCR segmentation to synaptic sites. We computed the F1 score, which is a common metric for the similarity between two segmentations calculated as the harmonic mean of precision and recall (see Methods section), as a function of intersection over union (IOU) threshold (**Fig. 2k**). IOU gives the fraction of voxels in the overlap region relative to the total number of voxels in the volume covered by either of the segments. The IOU threshold is a user-defined value that sets the degree of overlap between test and reference segments used for classification as true positive, false positive, and false negative detections. Here, *true positive* detections corresponded to $pSCR_{prediction}$ segments that had at least one $pSCR_{immuno}$ segment with IOU above threshold, resulting in the number of true positives N_{TP}. $pSCR_{immuno}$ segments that lacked a corresponding $pSCR_{prediction}$ segment with IOU above threshold were counted as *false negative* detections (number of false negatives: N_{FN}). Conversely, $pSCR_{prediction}$ segments that lacked a $pSCR_{immuno}$ segment with IOU above threshold were classified as *false positive* (number of false positives: N_{FP}). These were then used to calculate recall and precision. Recall (R) is defined as the number of correctly predicted objects relative to the total number of objects that should have been predicted ($R=N_{TP}/(N_{TP}+N_{FN})$). Precision (P) is given as the number of correctly detected objects in relation all detected objects ($P=N_{TP}/(N_{TP}+N_{FP})$. Both R and P vary with the threshold chosen for IOU and the F1 score ($F1=2*P*R/(P+R)$) combines these two into a number between 0 and 1.

As expected from the high Pearson correlation between immunolabeled and predicted BASSOON, a large fraction of $pSCR_{immuno}$ segments were recaptured when applying predicted BASSOON as guidance. When requiring at least 20% voxel overlap (IOU threshold=0.2), the F1 score was 0.84, corresponding to P=0.87 and R=0.82. Concretely, out of 412 synaptic transmission sites identified based on the BASSOON deep learning prediction, 358 had a corresponding segment based on BASSOON immunolabeling (true positives), whereas 81 of the BASSOON immunolabeling based segments were missed (false negatives). 54 of prediction-based segments did not have a corresponding immunolabeling-based segment (false positives). The deep learning pipeline is thus more likely to miss segments than to generate spurious ones. As expected, in this setting, the F1 score varied only slowly as a function of IOU threshold, as the shape of the pSCR segments was created identically in both cases by thresholding the CATS signal. Accordingly, segmentations only differed in which segments were retained based on spatial coincidence with either predicted or immunolabelled

BASSOON signal. For comparison, we also plotted the F1 score as a function of the Dice coefficient (2 times the number of voxels in the overlapping volume divided by the sum of the number of voxels of the individual volumes), another metric of segment overlap in common use.

We next tested whether preprocessing volumetric coCATS data with N2V had an appreciable effect on the quality of prediction of BASSOON location (**Supplementary Fig. 8c,d**), again testing on a pSCR segmentation level. Results were very similar regardless of whether the image translation model was trained on coCATS data that was preprocessed with or without denoising by N2V.

We then proceeded to testing whether training the image translation model for prediction of molecule location on super-resolved CATS plus super-resolved immunolabeling data had a benefit over training on super-resolved CATS plus immunolabeling data obtained with conventional confocal imaging (**Supplementary Fig. 8e,f**). Here, we saw a pronounced difference in the accuracy of BASSOON prediction, which is reflected in a lower curve for the F1 score when using diffraction-limited BASSOON signal for training. For a meaningful comparison, we matched the total volumes of super-resolved and diffraction limited BASSOON imaging data used for training.

In a final validation, we tested the similarity between automatically generated pSCR segments and segments generated by a human annotator (**Supplementary Fig. 8g-i**) in a volumetric dataset comprising 503 synapses. The human annotator took both the CATS data and the BASSOON immunostainings into account, both being 3D super-resolved by STED microscopy. They used segments generated by simple thresholding without prior background subtraction as starting point and proofread them, as in **Fig 2a**. $pSCR_{immuno}$ and $pSCR_{prediction}$ segments were automatically generated using the refined preprocessing pipeline described above. Comparing $pSCR_{immuno}$ segmentations (without human intervention) against the manually proofread versions tested how well our simple thresholding algorithm for immunolabelled BASSOON captured BASSOON location and how well the automated thresholding of the CATS data reflected pSCR shapes. Evaluating $pSCR_{prediction}$ segmentations in this way further included the quality of deep-learning based prediction of BASSOON location. Overall, the fully automated processing pipeline detected a large fraction of synapses correctly, as evidenced by the high F1 score at moderate IOU thresholds when comparing against the proofread "ground truth" (F1=0.82 and 0.71 for immunolabeling and prediction based segments, respectively, at IOU threshold=0.2). As expected, manual proofreading/ segmentation removed and added segments relative to the automated approaches, as illustrated in **Supplementary Fig. 8i**. This is mostly reflected by the difference of the F1 scores from 1 at moderate IOU thresholds. Differences in segment shapes between automated thresholding and manually generated segments additionally led to a progressive decline in F1 scores with increasing IOU threshold.

Note that determining the F1 score as a function of IOU threshold accounts for differences in segment shapes. F1 scores reported for automated synapse detection[1–4] in the EM literature vary according to the precise approach and testset size (see e.g. Dorkenwald[1] *et al.*, Fig. 2 for an overview). Commonly, F1 is used to quantify *detection* of synapses rather than synapse segment shapes. Further major differences between synapse detection in our work and EM are the possibility to take molecular information into account in CATS, whereas in EM, also

intracellular structural elements, in particular membrane bound synaptic vesicles, are visualized and spatial resolution is higher.

Supplementary Fig. 8

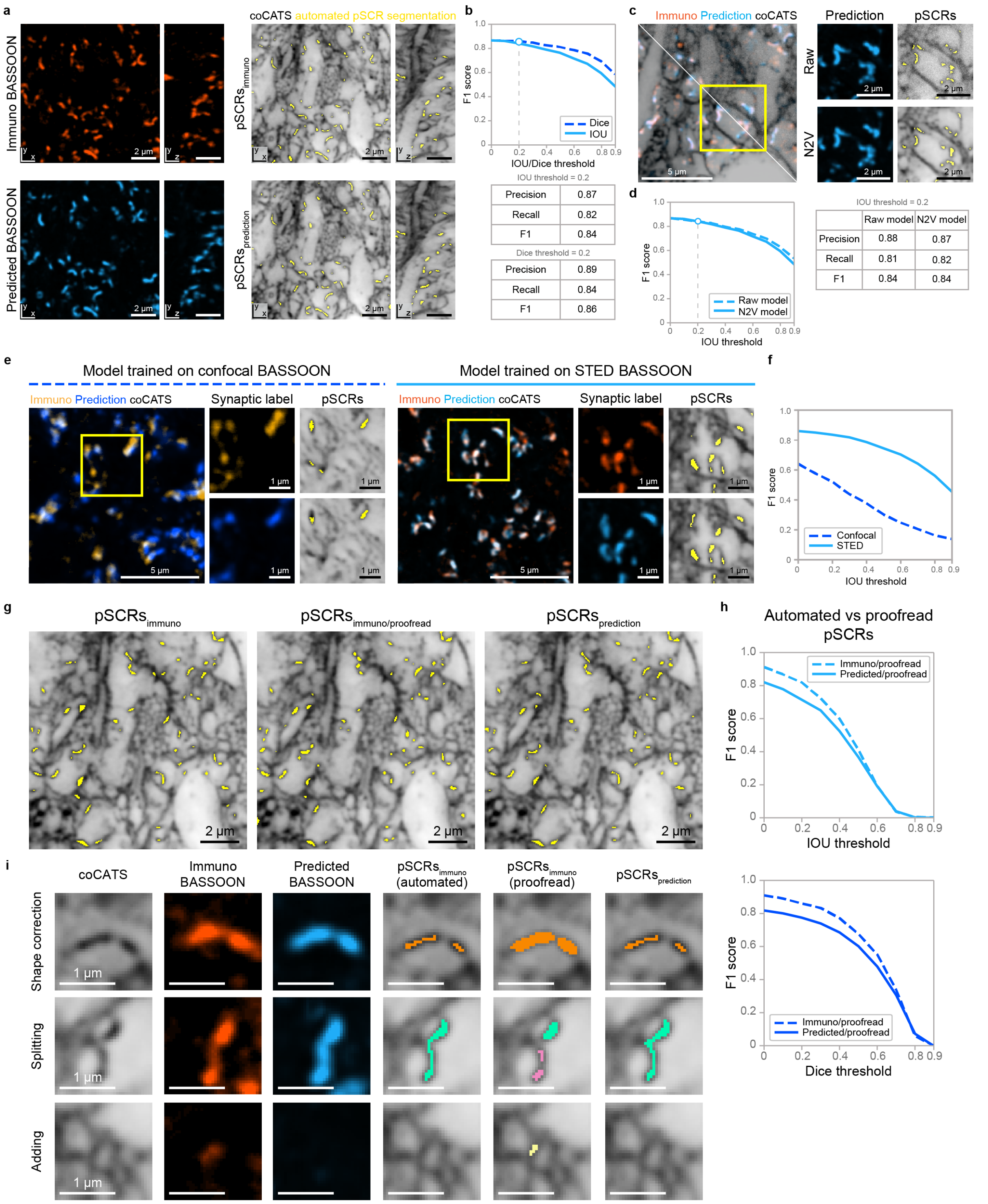

Supplementary Fig. 8| Validation of deep-learning based prediction of synaptic marker location and pSCR segmentation from coCATS structural data. coCATS datasets were acquired after in vivo microinjection and combined with immunostaining for BASSOON (both channels recorded volumetrically with a *z*-STED pattern for predominantly axial resolution increase, unless specifically noted). Same dataset as in Fig. 2j. CATS data were denoised with N2V, unless otherwise noted. Then a smoothing/background subtraction step was applied,

followed by grayscale erosion and thresholding. Segments in spatial proximity to either immunolabeled or predicted BASSOON were retained. Validation datasets were not included in training of the convolutional neural network for image translation. **a,** (*Left*) Comparison of *xy*- and *xz*-planes for immunolabeled BASSOON (*top*) and deep-learning-predicted BASSOON location (*bottom*). (*Right*) coCATS channel (gray) with corresponding automated pSCR segmentations (yellow) based on immunolabeled BASSOON (*top,* pSCRs$_{immuno}$) and predicted BASSOON (*bottom,* pSCRs$_{prediction}$). Both for immunolabeled and predicted BASSOON, automated pSCR segmentations without proofreading are displayed. **b,** F1 score (incorporating precision and recall) for comparing pSCR segmentation based on predicted BASSOON signal against pSCR segmentation based on immunolabeled BASSOON signal as a function of intersection over union threshold (IOU threshold) and Dice coefficient threshold (see Methods). F1, precision, and recall in tabular form for IOU threshold 0.2 and Dice coefficient threshold 0.2, indicating overall high performance of the deep-learning based model for pSCR prediction. No proofreading applied to the segmentations. **c,** Comparison of prediction of BASSOON signal and pSCR segmentation by two models trained on denoised vs. raw coCATS data. (*Left*) Immunolabeled (orange) and predicted (blue) BASSOON based on either raw or denoised CATS data. Magnified views of predicted BASSOON and pSCR segmentations for raw (*top*) and N2V (*bottom*) CATS data. Segmentation results are similar. **d,** Similar performance is also evident in the F1 score as a function of IOU threshold and in the table of performance parameters at IOU threshold 0.2, benchmarking the prediction of synaptic marker location by models trained on raw coCATS data and on N2V coCATS data against the immunostaining. **e,** Comparison of network models for prediction of BASSOON signal trained on super-resolved coCATS (*z*-STED) plus super-resolved BASSOON immunostaining (*z*-STED) vs. training on super-resolved coCATS (*z*-STED) plus confocal immunostaining data. (*Left*) Training based on confocal BASSOON with magnified view of the boxed region for immunostained BASSOON, predicted BASSOON signal, and corresponding pSCR segmentations. (*Right*) Training using super-resolved BASSOON. **f,** F1 score as a function of IOU threshold for confocal vs. STED models. The performance is markedly lower when using confocal BASSOON data, showing that the higher resolution in STED imaging is necessary to train a model to faithfully predict synaptic marker location and localize pSCRs in the deep-learning based segmentation pipeline. **g,** Comparison of pSCR segmentations (yellow) guided by immunolabeling BASSOON (*left,* pSCRs$_{immuno}$), manually proofread taking the immunolabeling into account (*middle*, pSCRs$_{immuno/proofread}$), and guided by BASSOON prediction (*right*, pSCRs$_{prediction}$) in the same dataset as in a. **h,** F1 score as a function of IOU threshold and Dice coefficient threshold comparing the respective automatically generated pSCR segmentations with the proofread version. **i,** Close-up view with examples of differences between proofread and purely automatically generated segmentations.

coCATS in vivo microinjection into the lateral ventricle as used here was performed in *n*=10 biological specimens. The N2V-deep-learning (DL) model in panels a-d,f,i was trained on *n*=13 denoised volumetric imaging datasets recorded across 4 brain sections coming from 3 animals (*n*=3 biological replicates). The raw-DL model in panels c,d was trained on the same datasets without denoising. The DL model trained on confocal BASSOON (panel e) was trained on *n*=8 volumetric imaging datasets recorded across 3 brain slices from 2 animals. The training data for the DL model based on STED-BASSOON in panel e was size-matched to training on confocal BASSOON and consisted of *n*=9 volumetric imaging datasets recorded across 4 brain slices from 3 animals.

Supplementary Fig. 9

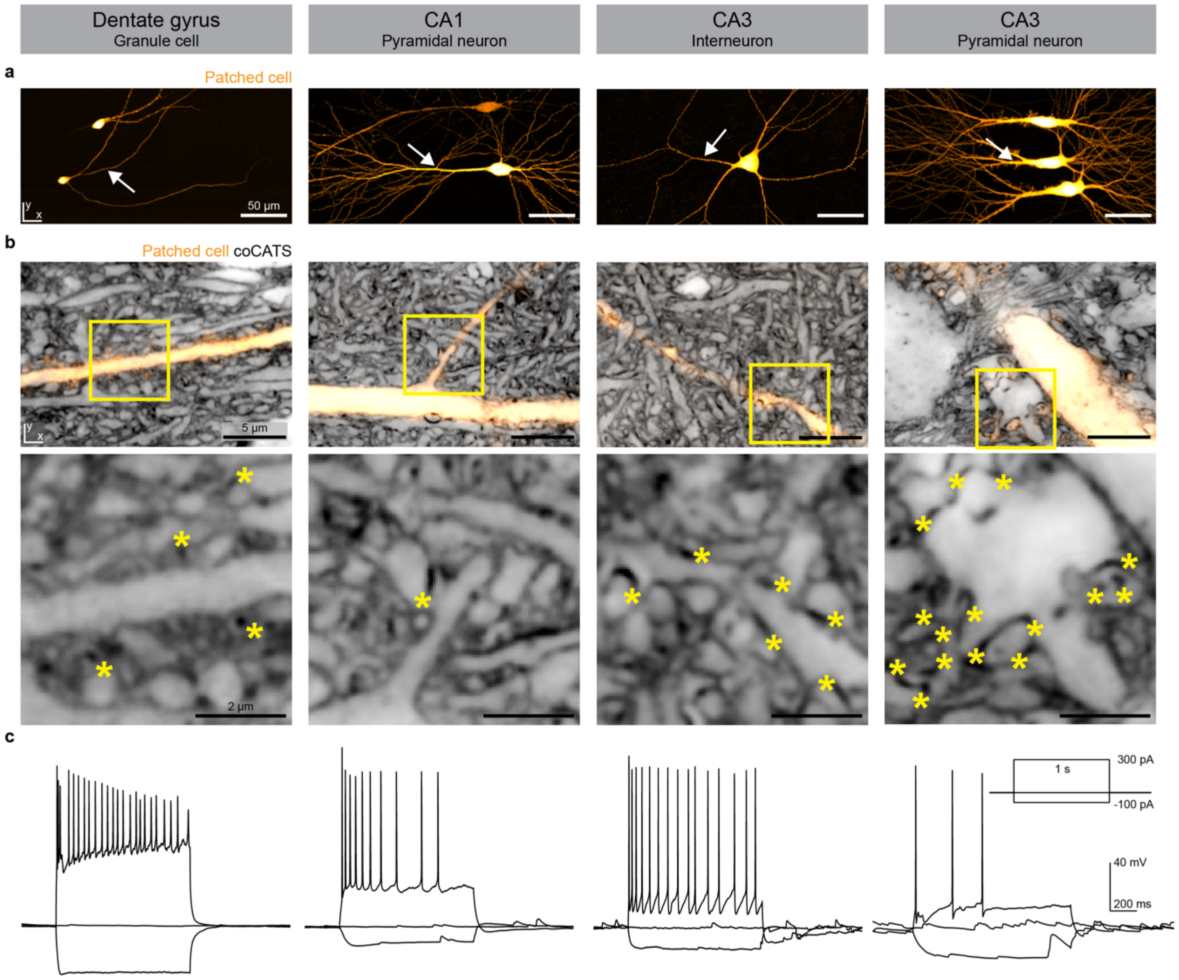

Supplementary Fig. 9| Combined structural and functional characterization with coCATS and single-cell patch-clamp experiments in various neuronal cell types. Neurons in organotypic hippocampal slice cultures were whole-cell patch-clamped, recorded and dye-filled with Lucifer yellow before coCATS labeling, immersion-fixation and imaging. **a,** Maximum intensity projection (MIP) overview images of various patched cells (DG granule cell, CA1 pyramidal neuron, CA3 interneuron, CA3 pyramidal neurons) acquired with a confocal microscope. Scale bars: 50 µm. **b,** (*Top*) Single *xy*-planes of volumetric imaging datasets showing close-ups (white arrows in panel a) of the same cells (yellow, confocal, N2V) in their tissue context revealed by coCATS (gray, *z*-STED, N2V). (*Bottom*) Zoomed views of boxed regions. coCATS visualizes the local environment of the cells, as well as the synaptic connectivity profile via pSCRs (asterisks). This information is useful for characterizing cell types and their connectivity also in the absence of an immunostaining. Scale bars: 5 µm. **c,** Unique action potential phenotypes upon current injection into the patched cells provide complementary information for cell type identification and single cell characterization. coCATS labeling in combination with functional recordings and dye-filling of various cell types was performed in 6 organotypic brain sections (n=6 biological specimens).

Supplementary Fig. 10

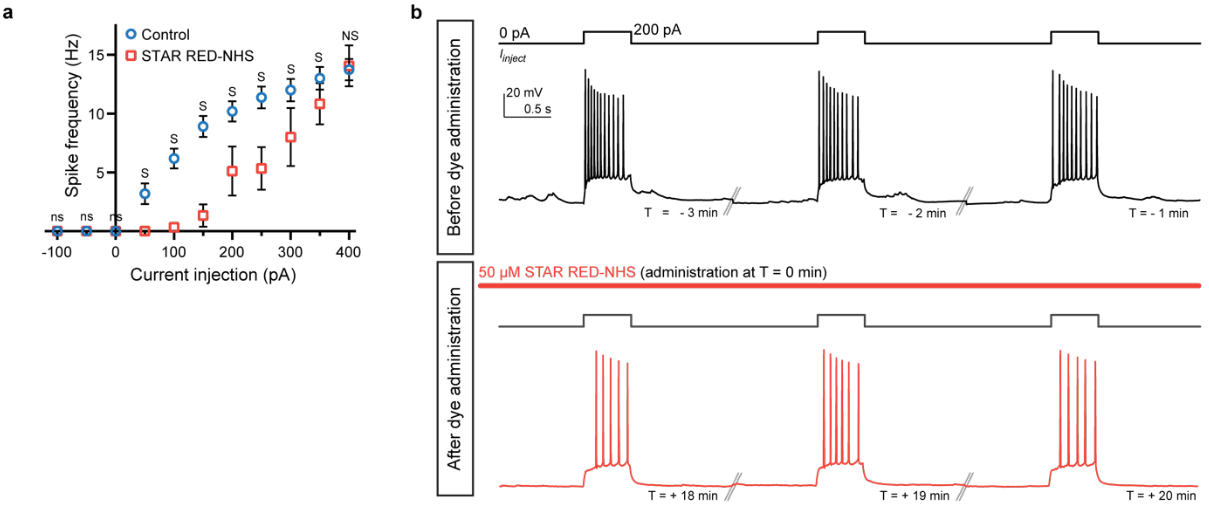

Supplementary Fig. 10| Neuronal activity during and after coCATS incubation. a, Action potential frequency as a function of current injection in CA1 pyramidal neurons in 15-16 DIV organotypic hippocampal brain slices without coCATS labeling (control) and after 25 min incubation with 50 µM STAR RED-NHS (mean ± s.d.). While viability and maximum firing rate (NS: non-significant, p=0.592, unpaired two-sided t-test) were not affected, lower spiking frequency was observed at lower injection currents in coCATS-incubated samples (50-350 pA, S: significant, p<0.000001 for 50 pA, 100 pA, 150 pA, 200 pA and 250 pA; p=0.000097 for 300 pA; p=0.002529 for 350 pA, unpaired, two-sided t-test). This effect likely results from an increase in network activity during dye incubation. Such increased network activity can commonly be observed in hyperconnected organotypic cultures. Measurements were performed in cultures prepared at three different time points and data points represent 11 control cells recorded across 3 slices (*n*=11 cells), and 9 cells recorded across 4 dye-exposed slices (STAR RED-NHS, *n*=9 cells). **b,** Firing profile of an example CA3 pyramidal neuron in response to current injection in an organotypic hippocampal brain slice at 3 time points spaced by 1 minute before (*top*) and at 3 time points after application of the coCATS label (*bottom*), spaced by 1 minute and starting 18 minutes after label application. CATS labeling did not prevent cell firing which would be indicative of cell damage. Electrophysiological recording during dye-incubation (panel b) was performed in *n*=3 biological specimens.

Supplementary Fig. 11

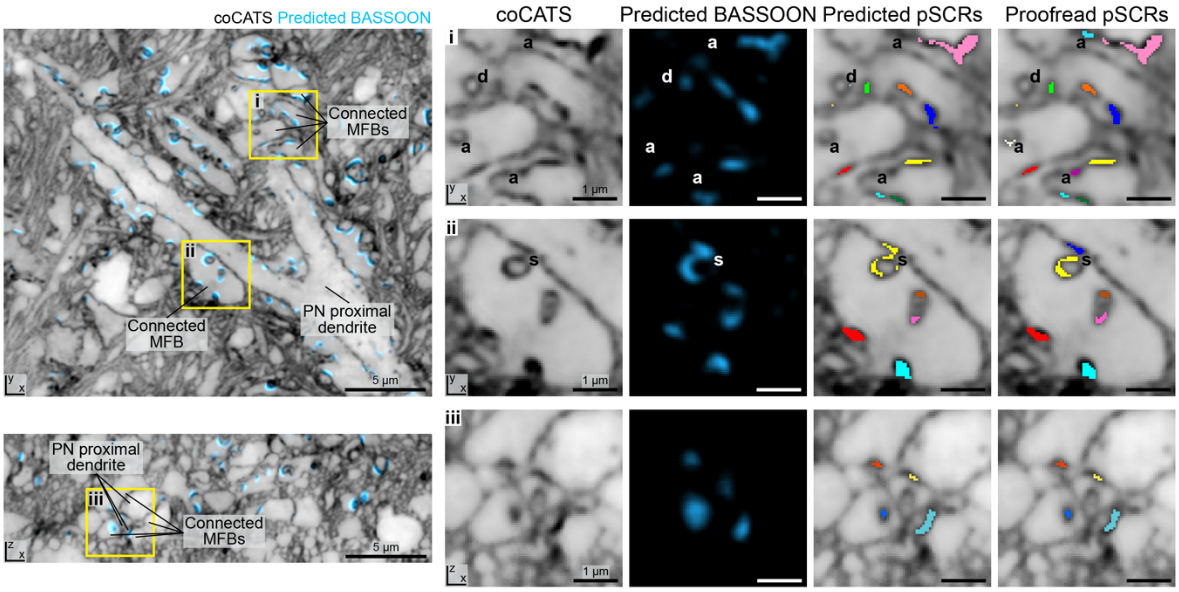

Supplementary Fig. 11| Deep-learning-based identification of synapse location in coCATS labeled organotypic hippocampal slice cultures. CA3 pyramidal neuron (PN) proximal dendrite as shown in Fig. 3g-i with synaptically connected mossy fiber boutons (MFB) identified via the presence of pSCRs. (*Left*) Single *xy*- and *xz*-planes of near isotropically super-resolved coCATS (N2V) data and BASSOON signal predicted by the deep learning algorithm (blue). (*Right*) Zoomed views of the boxed regions showing the CATS and BASSOON prediction channels separately, as well as automatically generated and manually proofread pSCR instance segmentations (both multi-colored). Specific examples of proofreading operations include adding (a), deleting (d), splitting (s), and merging (not shown) segments as indicated. For automatically generated pSCR segmentations, coCATS data was thresholded, without applying the smoothing/background subtraction and grayscale erosion steps discussed in Supplementary Fig. 8. coCATS labeling in combination with functional recordings and dye-filling of various cell types was performed in 6 organotypic brain sections (*n*=6 biological specimens). The data displayed here stems from a single imaging volume. pSCR segments were identified by the DL model in Fig. 2j-k and proofread manually.

Supplementary Fig. 12

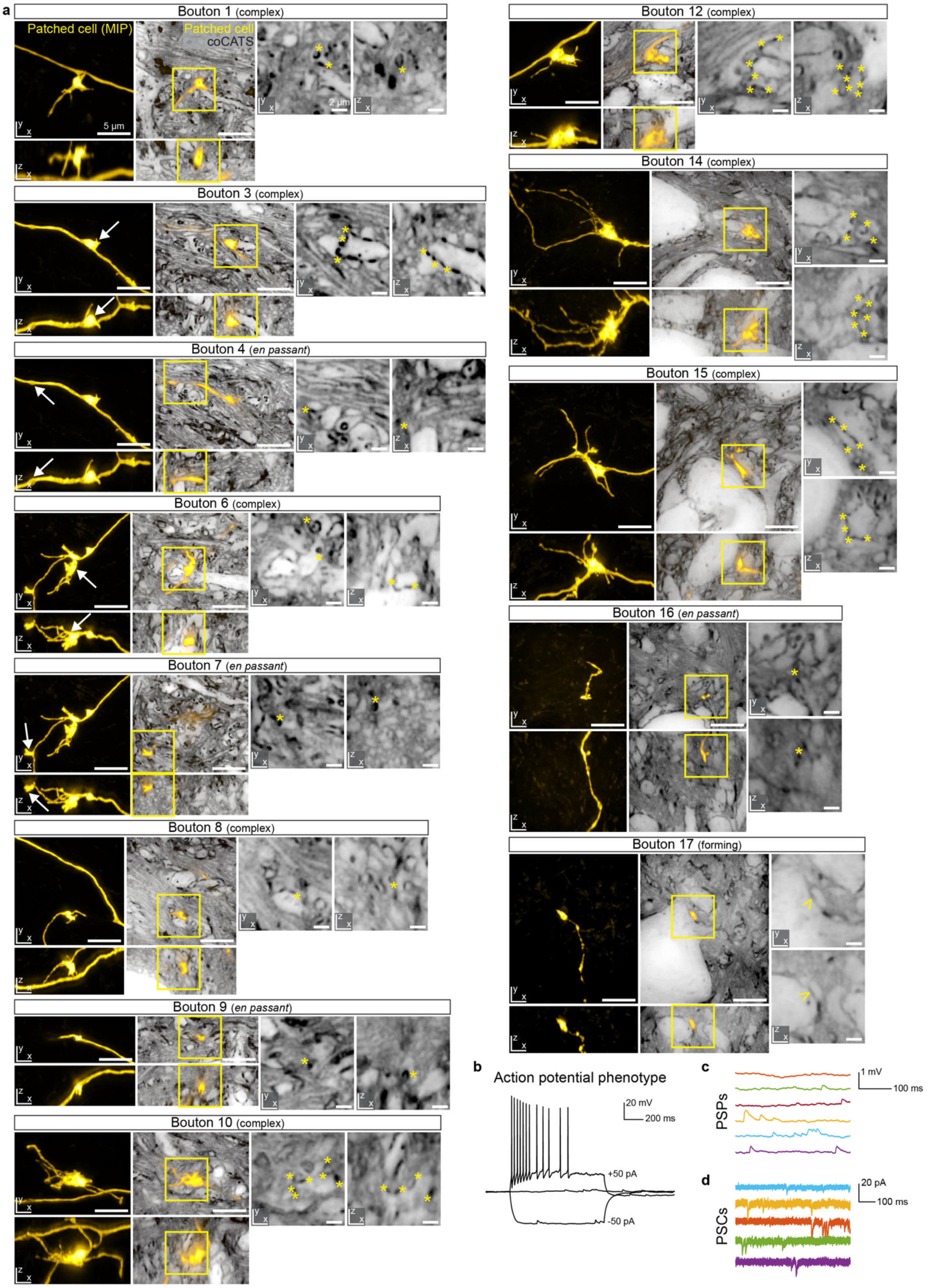

Supplementary Fig. 12| Visualization of DG granule cell output field. a, Tissue architecture around MFBs for 13 out of the 17 synaptic outputs marked in Fig. 4a, in addition to the 4 displayed in Fig. 4c. (*Left*) Maximum intensity projections (MIP) of positively labeled neuron

(yellow, *z*-STED, N2V). (*Middle*) Single *xy*- and *xz*-planes from volumetric measurements of surrounding tissue architecture by coCATS (*z*-STED, gray, N2V) with overlaid positive label of the single neuron. (*Right*) Zoomed views as indicated by the boxes showing the CATS channel alone with asterisks indicating pSCRs. White arrows in the left panels indicate the synaptic bouton that is displayed in the CATS panels for cases with more than one bouton in the field of view. At bouton 17, small yellow arrows mark the end-point of the axon without a clear pSCR present. Scale bars, overview images: 5 µm; magnified views: 2 µm. **b,** Action potential phenotype of the same DG granule cell, elicited by current injection during whole cell patch clamp recording. **c,** Spontaneous post-synaptic potentials (PSPs) and post-synaptic currents (PSCs) in the same neuron. coCATS labeling in combination with functional recordings and dye-filling of various cell types was performed in 6 organotypic brain sections (*n*=6 biological specimens). All boutons positively labelled here belong to a single cell and were acquired across multiple imaging volumes along the axon in the same organotypic slice (*n*=1 biological specimen).

Supplementary Fig. 13

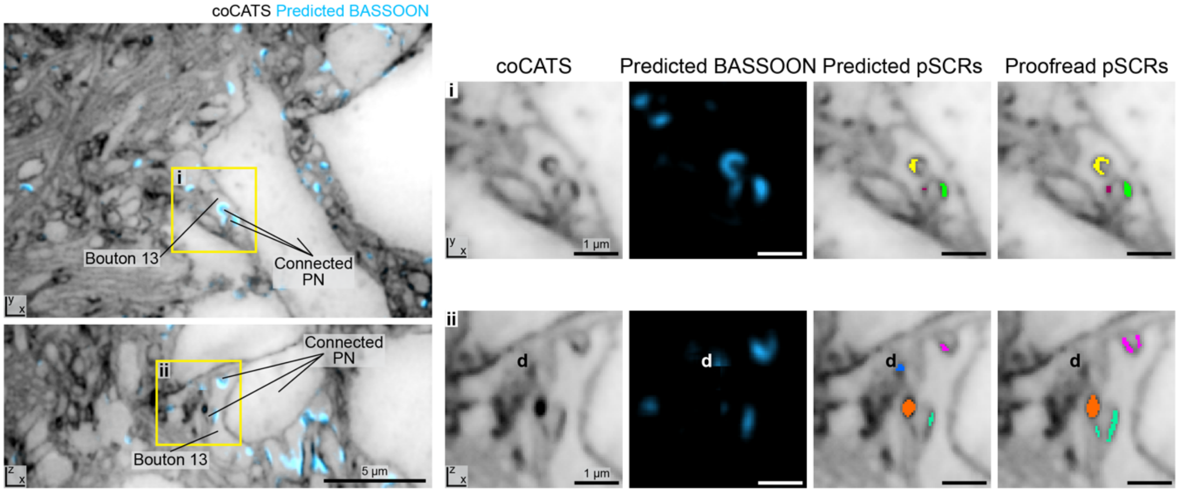

Supplementary Fig. 13| Deep-learning-based identification of synapse location in coCATS labeled organotypic hippocampal slice cultures. Imaging volume used for reconstructing the local output of bouton 13 of the DG granule cell in Fig. 4e. The location of bouton 13 of the positively labeled DG granule cell and a pyramidal neuron constituting its main post-synaptic partner (labeled 'Connected PN') are indicated. Scale bars, overviews: 5 µm; zoomed views: 1 µm. (*Left*) Single *xy*- and *xz*-planes of near-istropically super-resolved coCATS (N2V) volume and BASSOON signal predicted by deep learning algorithm (blue). (*Right*) Zoomed views of the boxed regions showing the CATS and prediction channels separately, as well as automatically generated and manually proofread pSCR instance segmentations (both multi-colored). In this specific example, a deletion operation (d) was performed during proofreading. For automatically generated pSCR segmentations, coCATS data was thresholded, without applying the smoothing/background subtraction and grayscale erosion steps of Supplementary Fig. 8. coCATS labeling in combination with functional recordings and dye-filling of various cell types was performed in 6 organotypic brain sections (n=6 biological specimens). pSCR segments were identified by the DL model from Fig. 2j-k and proofread manually.

Supplementary Fig. 14

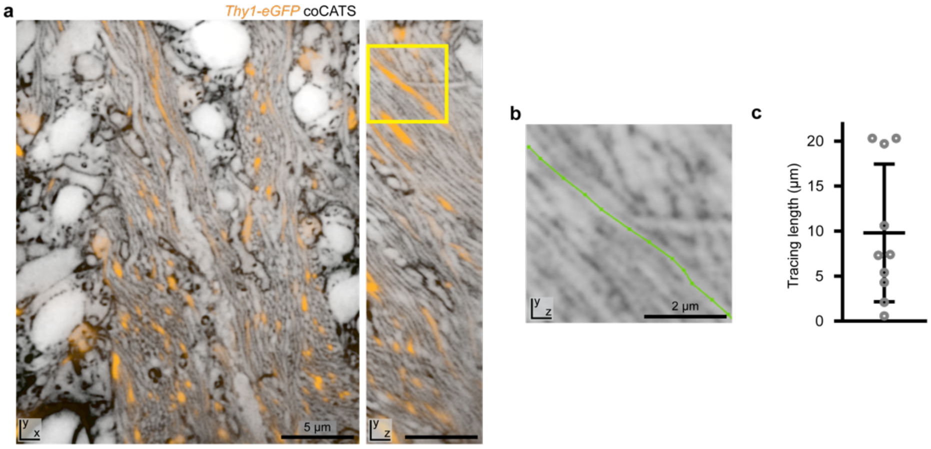

Supplementary Fig. 14| Tracing axons in coCATS data. a, Imaging volume from the hippocampal CA3 stratum lucidum of a *Thy1-eGFP* mouse after in vivo microinjection of coCATS label (STAR RED-NHS) and transcardial fixative perfusion. Sparse cytosolic expression of enhanced green fluorescent protein (eGFP) was detected with immunolabeling against eGFP using AF594. Both color channels were recorded at near-isotropic STED resolution and denoised with N2V. Power distribution in STED was 80/20 between the *z*-STED pattern and 4π helical phase modulation. **b,** Ten individual axons expressing eGFP were marked in the coCATS channel and handed over to a person blinded to the eGFP channel. That person was asked to trace these axons in both directions from the mark using WebKnossos. **c,** Length of correctly traced axon stretches for the ten axons, using eGFP expression as ground truth. Each data point corresponds to one axon from the dataset in panel a. Three axons could be traced across the entire dataset whereas most could only be followed for shorter stretches before an error occurred. Mean length of correctly traced axon stretches was 9.8 ± 7.6 µm (mean ± s.d.). Tracing was performed in n=10 axons from one imaging volume in n=1 biological specimen.

Supplementary Fig. 15

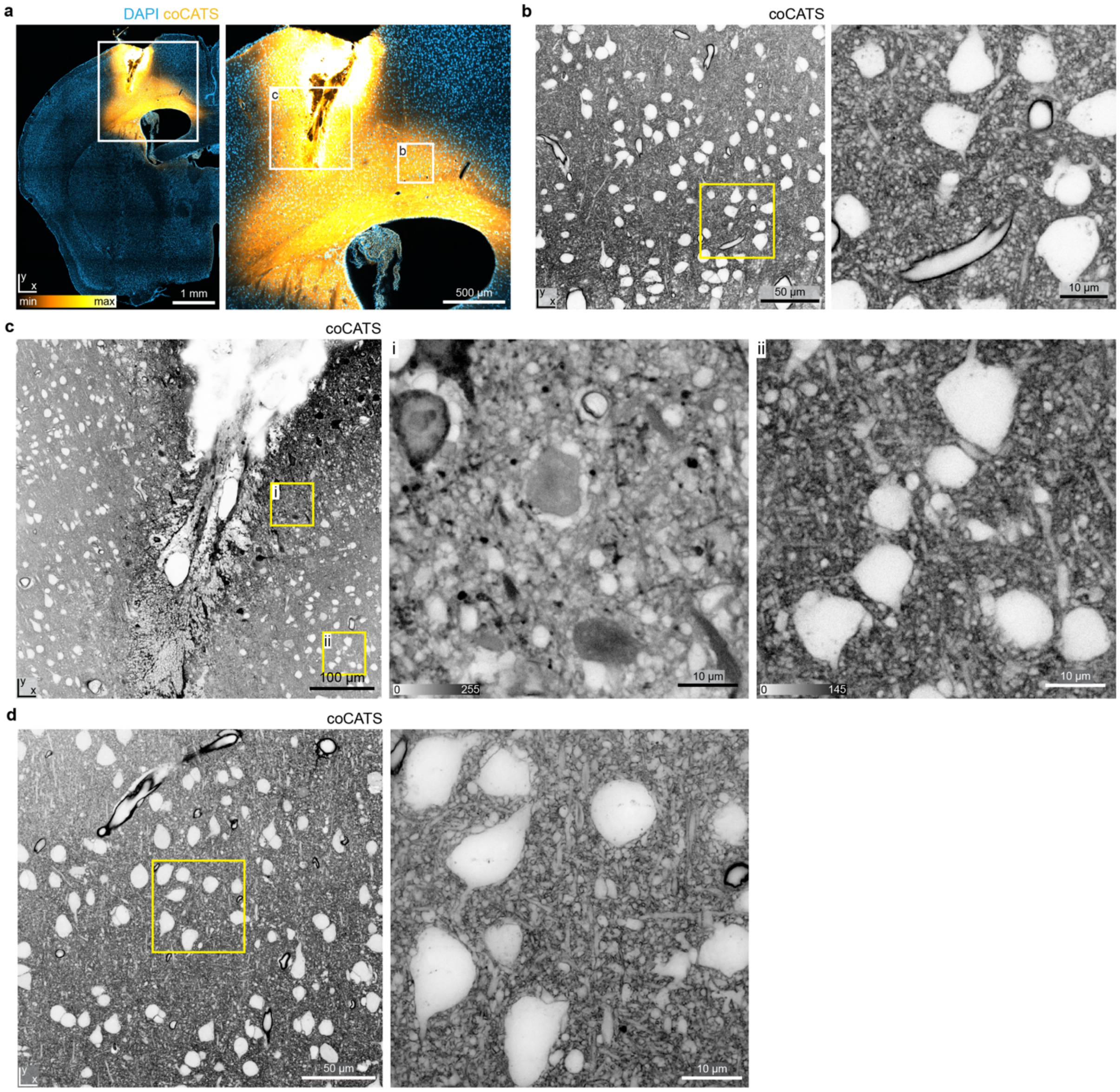

Supplementary Fig. 15| Targeted delivery of coCATS label to a specific brain region by in vivo microinjection. Brain areas of interest can be labeled by in vivo microinjection into the tissue close to the region of interest. **a,** Tile scan of a coronal brain section after in vivo microinjection into the primary motor cortex. The injection site, which displays local damage, is clearly visible. Higher resolution image of the area indicated by the white rectangle shows that the damage is spatially limited, such that cortical structures in proximity to the injection site can be studied. The coCATS intensity lookup table (yellow) is not inverted. Data was acquired on a spinning disc confocal microscope. **b,** Enlarged view of a cortical area spatially separated from the injection site. High labeling intensity, but no tissue damage, is visible in this dataset. Injection of coCATS label at ~0.2-1.0 mm from the region of interest yields high contrast labeling. The right panel is a magnified view of the boxed region. **c,** Enlarged view of a region comprising the injection site with two zoomed views, one in the damage region in immediate proximity to the injection site and one in the nearby region of intact tissue. Local damage is visible by the presence of erythrocytes and big voids in the overview image. (i) Enlarged view of a region in immediate vicinity of the injection site. Cellular structures appear

swollen and disorganized. Damaged cells are highly labeled (black), presumably because the coCATS label reacts with the protein-rich intracellular environment. Tissue structure cannot be appreciated as many structures have taken up labeling compound. (ii) Enlarged view of a region ~250 µm away from the injection site. No strongly labeled cellular structures are visible, and tissue organization, including cell bodies and processes, is visible. Images were acquired with a confocal microscope. **d**, Another region in the same coronal section. Overview confocal image (*left*) and zoomed view of the yellow-boxed region (*right*) acquired with STED microscopy show well-preserved tissue structure. Raw data. The data displayed here were acquired from the same biological specimen. coCATS in vivo microinjection into cortex was performed in 4 animals (n=4 biological specimens).

Supplementary Fig. 16

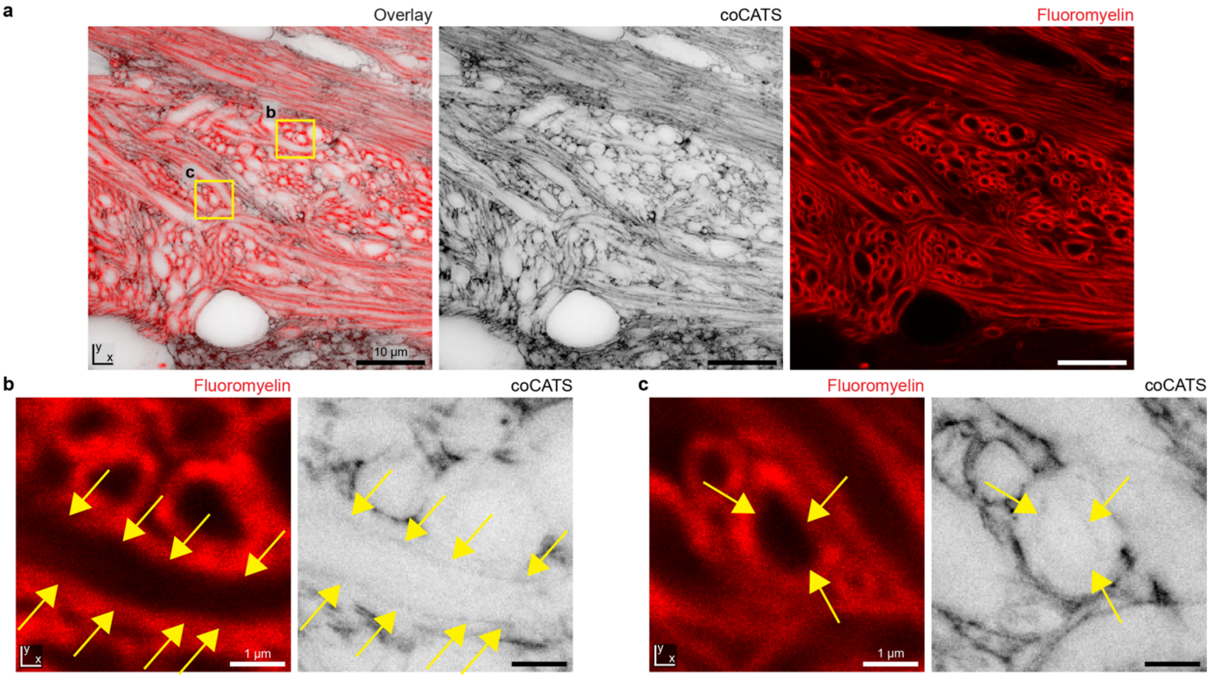

Supplementary Fig. 16| Visualizing myelinated axons with coCATS. a, Tissue architecture in the corpus callosum revealed by coCATS (gray, *xy*-STED) after in vivo microinjection of coCATS label (STAR RED-NHS) into the lateral ventricle of an adult mouse and transcardial fixative perfusion. Myelinated axons were labelled with FluoroMyelin (red, confocal). **b,c,** Magnified views of the regions indicated in panel a. coCATS delineates the outer border of the myelin sheath. In many of the myelinated axons, a demarcation of the axon itself (yellow arrows) at the inner border of the myelin sheath is visible, but the signal intensity is low. This ad-axonal line is more consistent in rCATS (see Fig. 5a, Supplementary Fig. 18). Raw data. coCATS combined with myelin labeling was performed in 2 brain sections (n=2 technical replicates) from one biological specimen.

Supplementary Fig. 17

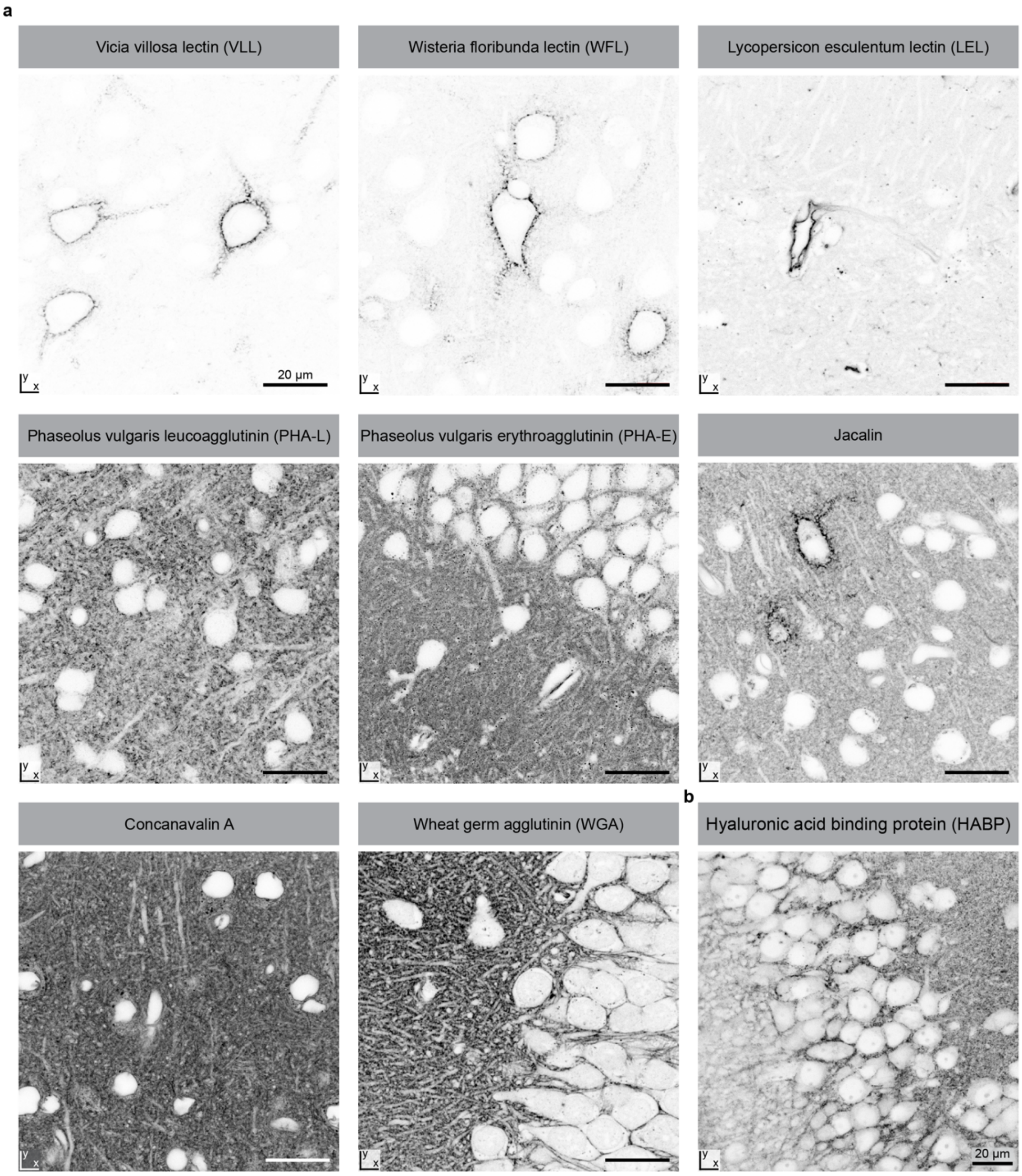

Supplementary Fig. 17| Screening for affinity binders to reveal tissue architecture with resident CATS (rCATS) in rodent brain. a, Binding pattern of various lectins in previously fixed adult mouse brain. (*Top, left*) Vicia villosa lectin (VVL) and (*top, center*) Wisteria floribunda lectin (WFL) binding patterns were restricted to perineuronal nets associated with a sparse subset of cells (cortex). These are thought to bind to terminal N-acetylgalactosamine linked to serine/threonine and galactose, respectively. (*Top, right*) Lycopersicon esculentum lectin (LEL) faithfully depicted blood vessels by binding to specific N-glycans (hippocampus). (*Middle, left*) Phaseolus vulgaris leucoagglutinin (PHA-L) and (*middle, center*) Phaseolus vulgaris erythroagglutinin are two members of the same lectin family that resulted in a grainy extracellular staining pattern in the adult mouse cortex (PHA-L) and dentate gyrus (PHA-E). (*Middle, right*) Jacalin binds to O-glycosidically linked oligosaccharides, leading to a strong labeling of perineuronal nets, as well as a weak depiction of the extracellular environment. (*Bottom, left*) Concanavalin A recognizes α-mannose on oligosaccharides. This resulted in a

homogeneous labeling of the extracellular space, here shown in cortex, but also of intracellular structures, including the nuclear envelope. (*Bottom, middle*) Wheat germ agglutinin (WGA) delineates cell bodies and cellular processes by binding to N-acetylglucosamine and sialic acid abundant in the extracellular matrix and on cell surfaces, as shown here for hippocampus. Scale bars: 20 µm. **b,** Hyaluronic acid was labeled via fluorescently labeled hyaluronic acid binding protein (HABP) in the mouse hippocampus. Cellular outlines are visible, but the labeling is not homogeneous. Images were acquired with a confocal microscope. Intensity lookup tables are inverted. After initial screening, involving n=3 biological replicates for WGA, this lectin was used for further experiments. The other lectins and HABP were not further pursued after testing in n=1 brain section each.

Supplementary Fig. 18

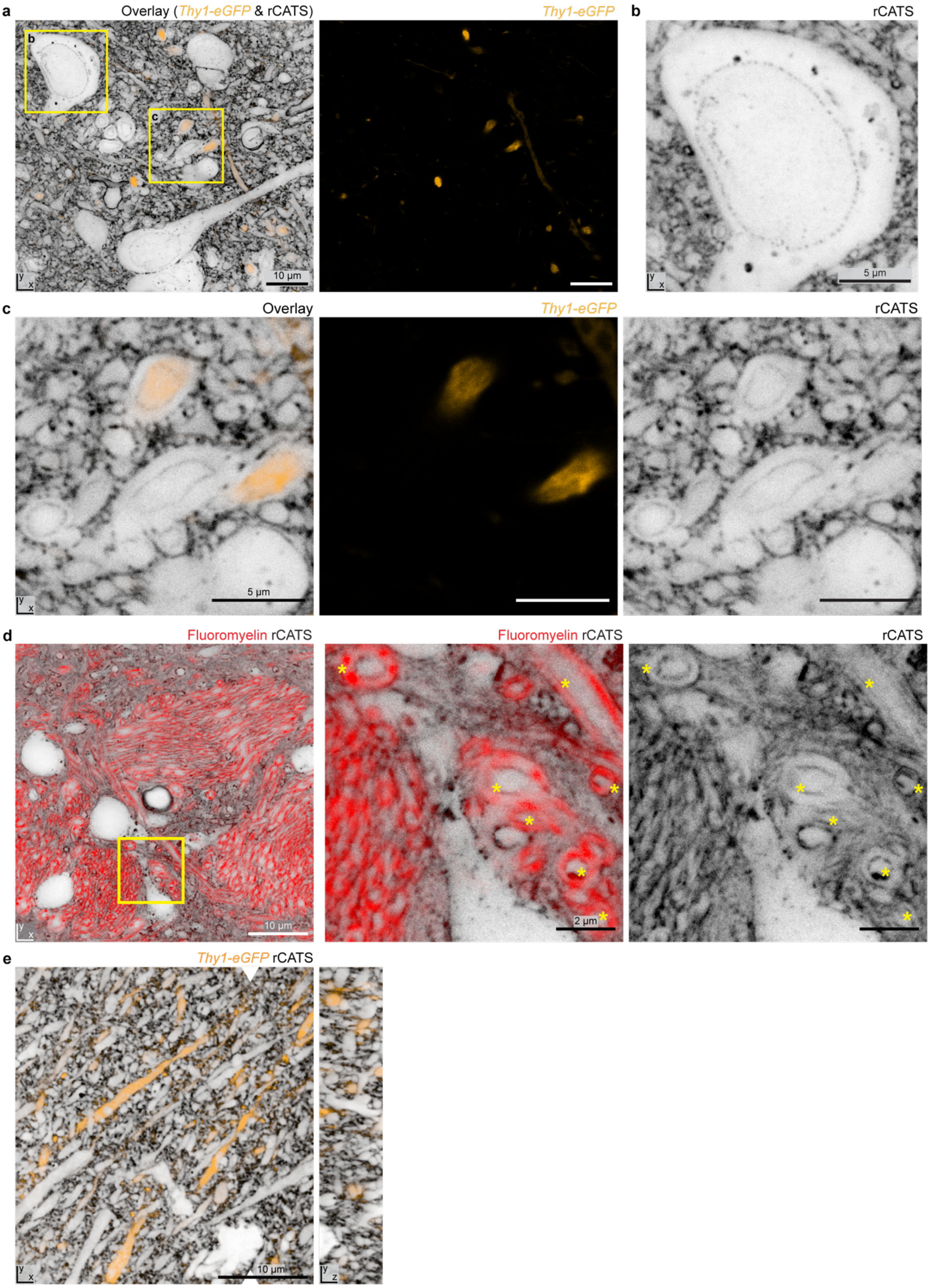

Supplementary Fig. 18| Identification of myelination and nuclear pores by rCATS. a, rCATS labeling in the hypothalamus of a perfusion-fixed adult *Thy1-eGFP* mouse. A sparse

subset of neurons are labeled via cytosolic eGFP expression (orange, confocal), shown in the overlay with rCATS (gray, STED with power distribution of *z*-STED/*xy*-STED patterns of 80/20) and as a separate panel. **b,** Magnified view of boxed region in a: Neuronal soma with nuclear envelope discernible in the rCATS channel due to binding of WGA to nuclear pore glycoproteins. **c,** Magnified view of second boxed region in a. Zoom-in on myelinated axons. Cytoplasmic expression of eGFP in a subset of neurons exclusively labels the axon but not the myelin sheath. In contrast, rCATS typically delineates the inner and outer borders of the myelin sheath. **d,** Fluoromyelin staining (red, confocal), as well as rCATS (gray, *xy*-STED), reveal myelinated axons in the hypothalamus of a perfusion-fixed adult mouse. Magnified view: myelinated structures delineated by rCATS co-localize with Fluoromyelin (examples indicated by asterisks). **e,** STED at near-isotropic resolution of rCATS labeling (gray, *z*-STED) in the cortex of a perfusion-fixed adult *Thy1-eGFP* mouse (eGFP: orange, confocal). White arrowheads indicate position of *yz*-view. rCATS in combination with sparse *Thy1-eGFP* labeling was performed in *n*=3 biological specimens. rCATS in combination with Fluoromyelin labeling in panel d was performed on 4 brain slices across *n*=3 animals.

Supplementary Fig.19

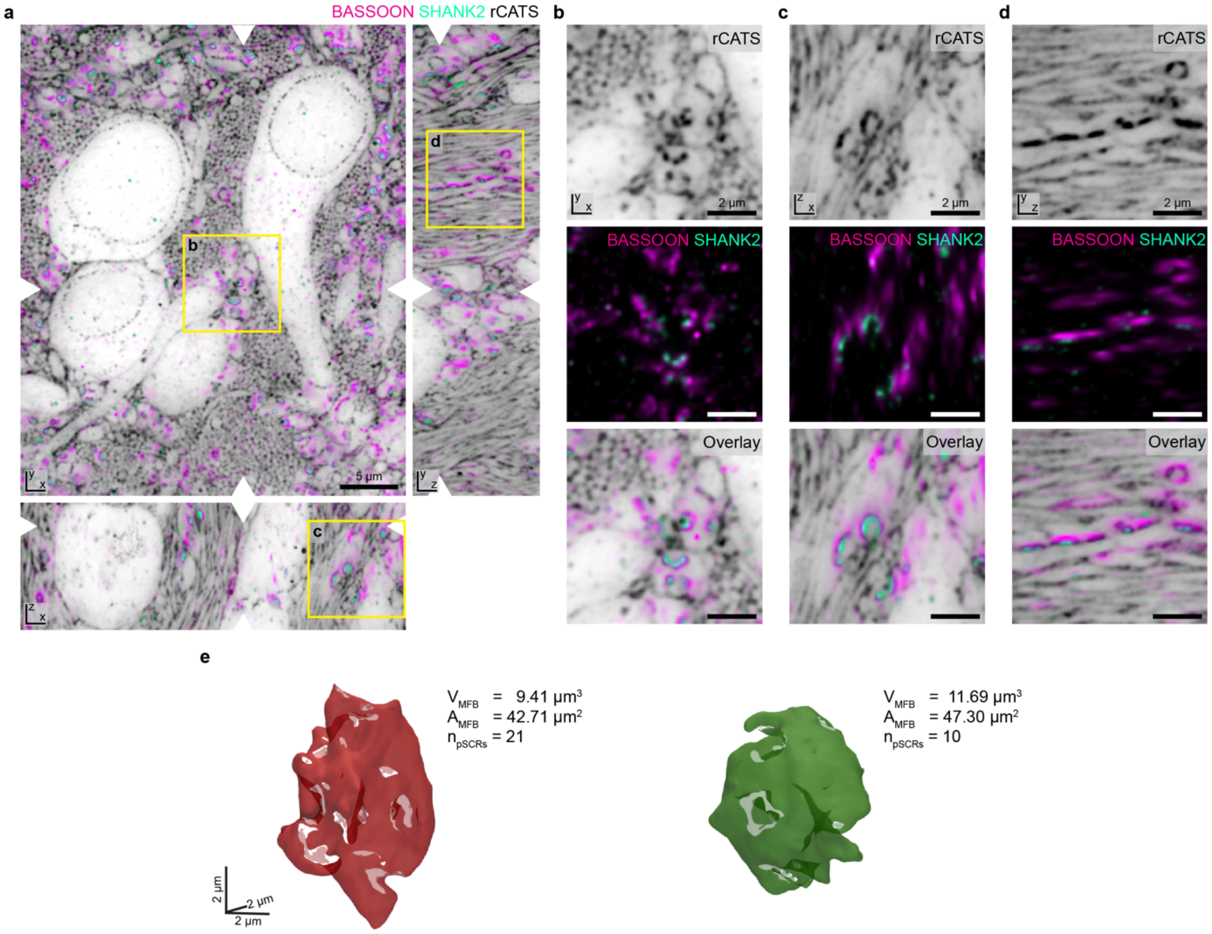

Supplementary Fig. 19| Organization of the DG hilus of an adult mouse revealed by rCATS. a, Three orthogonal views of an imaging volume in the DG hilus of an adult mouse after transcardial fixative perfusion. The rCATS channel (gray, *z*-STED, labeling with WGA-CF633) reveals cell somata, mossy fiber boutons and bundles of mossy fibers. Synapses are highlighted by immunolabeling for the presynaptic marker BASSOON (magenta, AF488, confocal). Immunolabeling for the postsynaptic scaffolding protein SHANK2 (turquoise, AF594, *z*-STED) highlights excitatory synapses. White arrowheads indicate positions of corresponding orthogonal sections. All channels were denoised with N2V. **b-d,** Magnified views of the orthogonal slices in regions indicated in panel a, highlighting several mossy fiber boutons and a neurite studded with synaptic transmission sites. High intensity features in the rCATS channel in close spatial association with synaptic molecules show similarity to pSCRS observed in coCATS. However, the labeling mechanism is different in the two cases. In coCATS, covalent labeling targeting primary amines, particularly on proteins, is expected to map the local extracellular protein density. In rCATS, lectin binding maps the presence of carbohydrate residues. **e,** 3D-renderings of two manually segmented MFBs with key geometric parameters. These fall into the same range as the quantifications in the CA3 stratum lucidum performed with coCATS in Fig. 2. White shaded areas on the MFBs represent putative synaptic transmission sites as defined by the dense carbohydrate rich features sandwiched between the pre- and postsynaptic markers. We applied the automated immunolabeling-guided pipeline developed for coCATS without any parameter tuning for an initial segmentation and manually corrected the output taking both the rCATS channel and molecular information into account.

rCATS in perfusion-fixed brain slices was performed in n=10 biological specimens. Exemplary MFB segmentation from rCATS data was performed for the 2 MFBs displayed here from one imaging volume.

Supplementary Note 2: Fixation, permeabilization and antigen retrieval protocols for CATS.

In coCATS, we employed both transcardial fixative perfusion after in vivo microinjection and, for organotypic hippocampal brain slices and human cerebral organoids, immersion fixation, in all cases using paraformaldehyde (PFA) as fixative. Here, the extracellular label is applied in the living state. Probe penetration is governed by tissue organization, as well as the half-life time of the reactive moiety. We mostly used NHS as amine reactive group, which is hydrolyzed in aqueous environment. For in vivo microinjection experiments, sufficient labeling density for high-resolution imaging could be achieved up to ~1 mm away from the injection site (see **Fig. 1c, Supplementary Fig, 2 and Suppl. Fig. 15**). We observed that diffusion of the coCATS probe in the tissue, and therefore labeling volume, was enhanced by the presence of axon bundles, such as those found in the corpus callosum (see **Supplementary Fig. 15**) and retarded by tightly packed cell layers. Additional permeabilization is not necessary for probe penetration if a fluorophore-containing covalent labeling molecule is used, as was typically the case in our STED experiments. Avoiding permeabilization led to best coCATS labeling contrast. In cases where antibody stainings were added to coCATS, permeabilization was required. For coCATS, we used a gentle permeabilization protocol with 0.2% Triton X-100 (TX) at 4°C. This was done to balance potential loss of signal due to washout of tissue components bearing coCATS labels vs. permeabilization requirement for antibody penetration. We did not extensively test different detergents.

rCATS caters to diverse sample types requiring specific considerations. For previously collected samples, e.g. human (archival) material, tissue preservation can often not be experimentally controlled. In mouse brain samples with optimum fixation conditions, we found that the quality of rCATS labeling was similar to the one achieved with coCATS (**Fig. 5a-c, Extended Data Fig. 8,9, Supplementary Fig. 19**), even though rCATS is dependent on the presence of extracellular carbohydrates whereas coCATS can be expected to label extracellular molecules more indiscriminately. However, for rCATS, permeabilization is required. We achieved best structural preservation with repeated freeze-thaw cycles but rCATS labeling was restricted to the first 20 µm of the tissue (**Supplementary Fig. 20**). It is of note that penetration depth is higher for rCATS labeling than for conventional antibodies, likely mirroring the larger size of antibodies (typically ~150 kDa) compared to WGA (~38 kDa). If deeper probe penetration is required, alternative permeabilization strategies are required. When using low concentrations of TX at low temperatures (e.g. 0.2% TX ON at 4 °C) we observed a similar labeling quality than the one obtained with the freeze-thaw method, such that we opted for this strategy for the expansion microscopy experiments. Yet, increasing the TX-concentration and temperature during permeabilization, as well as adding TX directly to the WGA labeling solution, resulted in a marked decrease in the quality of the rCATS staining (**Supplementary Fig. 20b-d**). In these cases, the staining appeared less homogeneous alongside the formation of aggregates. We note that we did not embark on further optimization of permeabilization protocols, and other mild detergents such as Saponin or Tween may well yield similar or superior results.

For human brain specimens, we obtained best structural preservation with surgery specimens depending on procedural aspects that impacted fixation. We hypothesized that tissue preservation was decreased in human samples as a result of being restricted to immersion fixation rather than perfusion fixation, especially in deeper tissue layers, which would be

apparent in the rCATS staining (**Fig.6a-c, Supplementary Fig. 24**). Indeed, when comparing perfusion- vs immersion-fixation in entire hemispheres of mouse brains (**Supplementary Fig. 20a**), we found that a less homogeneous staining was obtained in the immersion-fixed material. In addition, in human surgery specimens, some areas of the tissue showed mechanical damage from the surgical handling.

We also found rCATS labeling to be compatible with standard protocols for processing archival formalin-fixed paraffin-embedded (FFPE) tissues and with common antigen retrieval protocols required in these samples for antibody labeling. Here, the expectation is that brain biopsy samples (**Fig. 6e-i**) would be fixed more rapidly than postmortem material, resulting in typically better tissue preservation. However, rCATS was still applicable to a postmortem brain with a postmortem interval of over 12 hours that had been stored in the paraffin block for 16 years (**Fig. 6d**). We furthermore tested rCATS for compatibility with different antigen retrieval protocols for FFPE tissues (**Supplementary Fig. 27a-d**), employing both a low-pH citric acid-based protocol and a protocol at high pH according to the requirements of different antibodies that are in routine diagnostic use in the clinical histopathology setting.

Supplementary Fig. 20

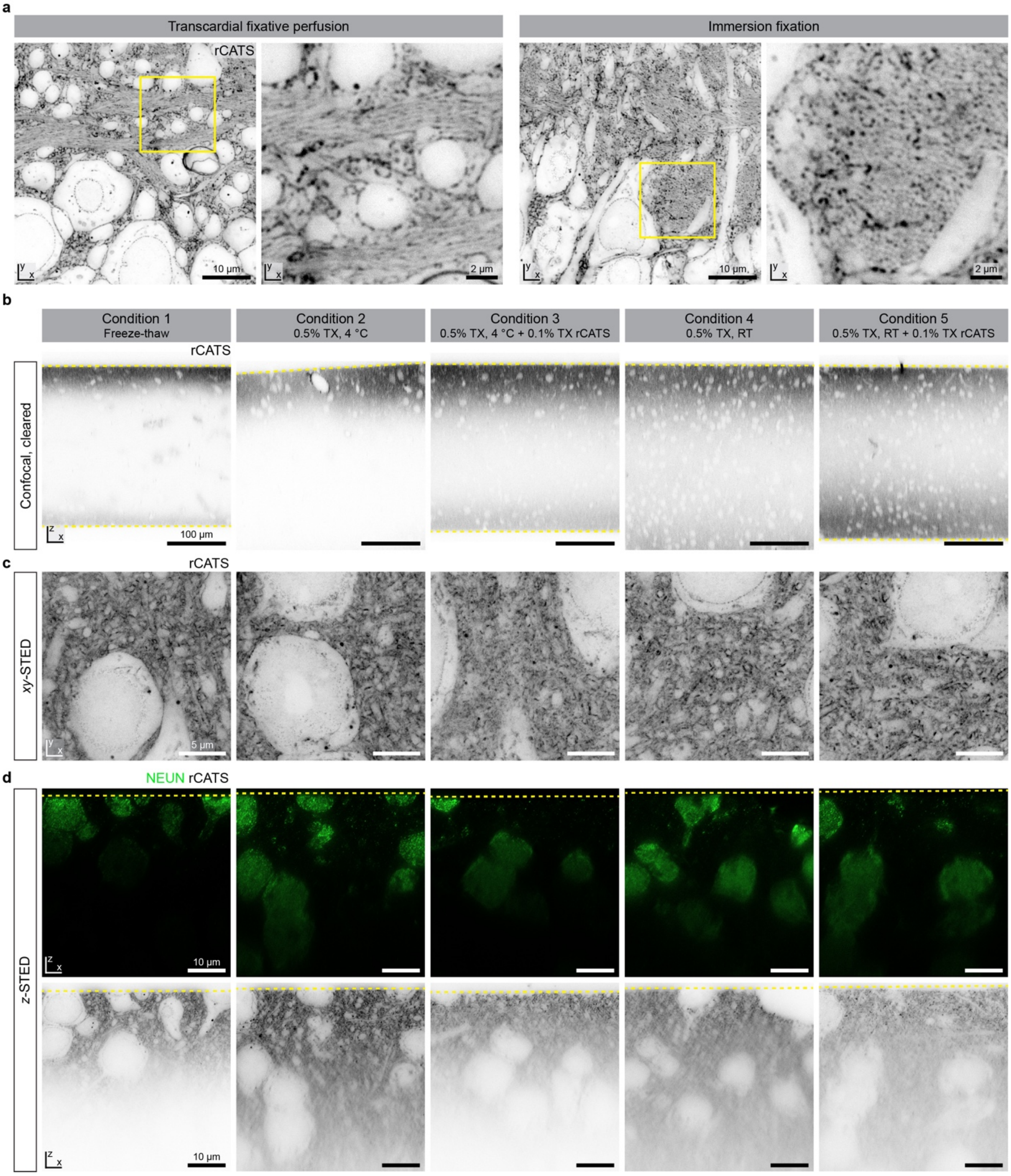

Supplementary Fig. 20| Effects of fixation and permeabilization conditions on rCATS labeling in mouse brain. a, Comparison of rCATS (WGA-CF633) labeling quality after transcardial fixative perfusion (*left*) and immersion fixation (*right*). Overview image (*xy*-STED) and magnified view of the boxed area in the hippocampal CA3 region. For immersion fixation, the brain was excised after sacrificing the animal, and hemispheres immersed in fixative solution. For permeabilization, repeated cycles of freezing and thawing were applied in both cases. While both methods are applicable, we typically preferred transcardial fixative perfusion for optimum preservation of specimen structure. **b-d,** Effect of different permeabilization conditions on rCATS (WGA-CF633) and antibody penetration in the cortex

of an adult mouse after transcardial fixative perfusion. 300 µm thick brain sections were permeabilized by repeated freeze-thaw cycles (condition 1), overnight incubation with a detergent (0.5% Triton X-100 (TX) at 4 °C, conditions 2, 3) or overnight incubation with 0.5% TX at room temperature (RT, conditions 4, 5). We furthermore tested whether addition of 0.1% TX in the rCATS/antibody solution would increase probe penetration (conditions 3, 5). Sample preparation was identical within each column. Tissue sections were chosen thicker than in our typical experiments (50-100 µm) in order to assess penetration of labeling reagents from either specimen surface. Top surfaces and, where included in the imaging volume, bottom surfaces of the tissue are indicated by yellow dashed lines. **b,** Depth penetration (*xz*-view) of rCATS labeling assessed by confocal microscopy. Samples were cleared with CUBIC 2 solution (50% sucrose, 25% urea, 10% triethanolamine, 0.1% TX in ddH_2O, refractive index ~1.4) after rCATS labeling. Imaging was performed with a multi-immersion 20x objective lens with silicone immersion oil. **c,** Close-up view with *xy*-STED near the surface to assess differences in labeling quality and structural preservation for the various conditions. **d,** Penetration depth of immunolabeling for the neuronal marker NEUN (AF594), paired with rCATS (WGA-CF633) labeling, for the various permeabilization conditions without clearing. Note that readout was performed with axial scans in *z*-STED mode for both color channels with the objective lens' correction collar set for the tissue surface, such that signal strength and imaging quality degraded beyond the first ~10 µm, in addition to any decline in labeling quality. This reflects the *z*-STED pattern's sensitivity to spherical aberrations from refractive index mismatch (compare characterization in Extended Data Fig. 1d, Supplementary Fig. 6g). We observed highest rCATS labeling quality when using freeze-thaw permeabilization. When applying detergent-based permeabilization, rCATS labeling intensity and homogeneity decreased with harsher permeabilization conditions, including increasing time and temperature of TX permeabilization. Including TX during WGA incubation had a detrimental effect on rCATS labeling quality. rCATS labeling in perfused brain slices, as seen in panel a, was performed in n=10 biological specimens. rCATS labeling of immersion-fixed half-hemispheres was performed once. The effect of permeabilization conditions on rCATS and antibody labeling depth (panels b-d) was tested twice, in n=2 independent biological specimens.

Supplementary Fig. 21

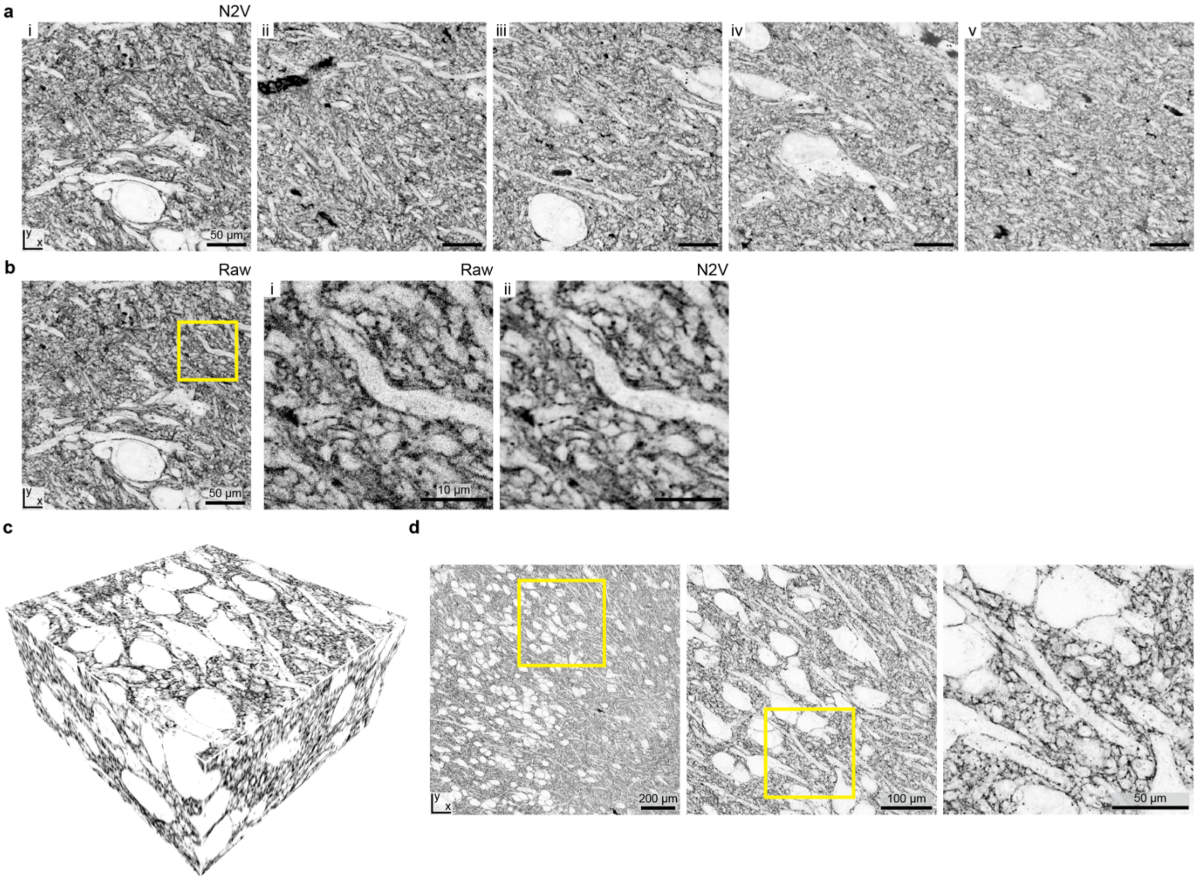

Supplementary Fig. 21| coCATS with ExM in organotypic hippocampal slice cultures. a, En face views of the 5 example planes in Fig. 5d (denoised with N2V). Scale bars refer to tissue size after expansion. **b,** Raw data for the first slice, with zoom in the boxed region for (i) raw data and (ii) data after denoising with N2V. **c,** 3D-view of a 290 x 290 x 137 μm^3 imaging volume after ~4-fold expansion of an organotypic hippocampal slice culture with protein retention ExM (proExM). The tissue was coCATS labeled with NHS-PEG_{12}-biotin, immersion-fixed, hydrogel-embedded, mechanically homogenized by proteolytic digestion, and expanded. Post-expansion readout was performed with fluorophore-labeled streptavidin. **d,** Overview image of a single *xy*-plane of the same sample and progressive zoom-ins as indicated by the rectangles. Data was acquired with a confocal microscope. Scale bars refer to sample size after expansion. coCATS labeling of organotypic brain sections in combination with MAP (panels a,b) was performed in n=3 biological specimens. coCATS labeling of organotypic brain sections in combination with proExM (panels c,d) was performed in n=3 biological specimens.

Supplementary Fig. 22

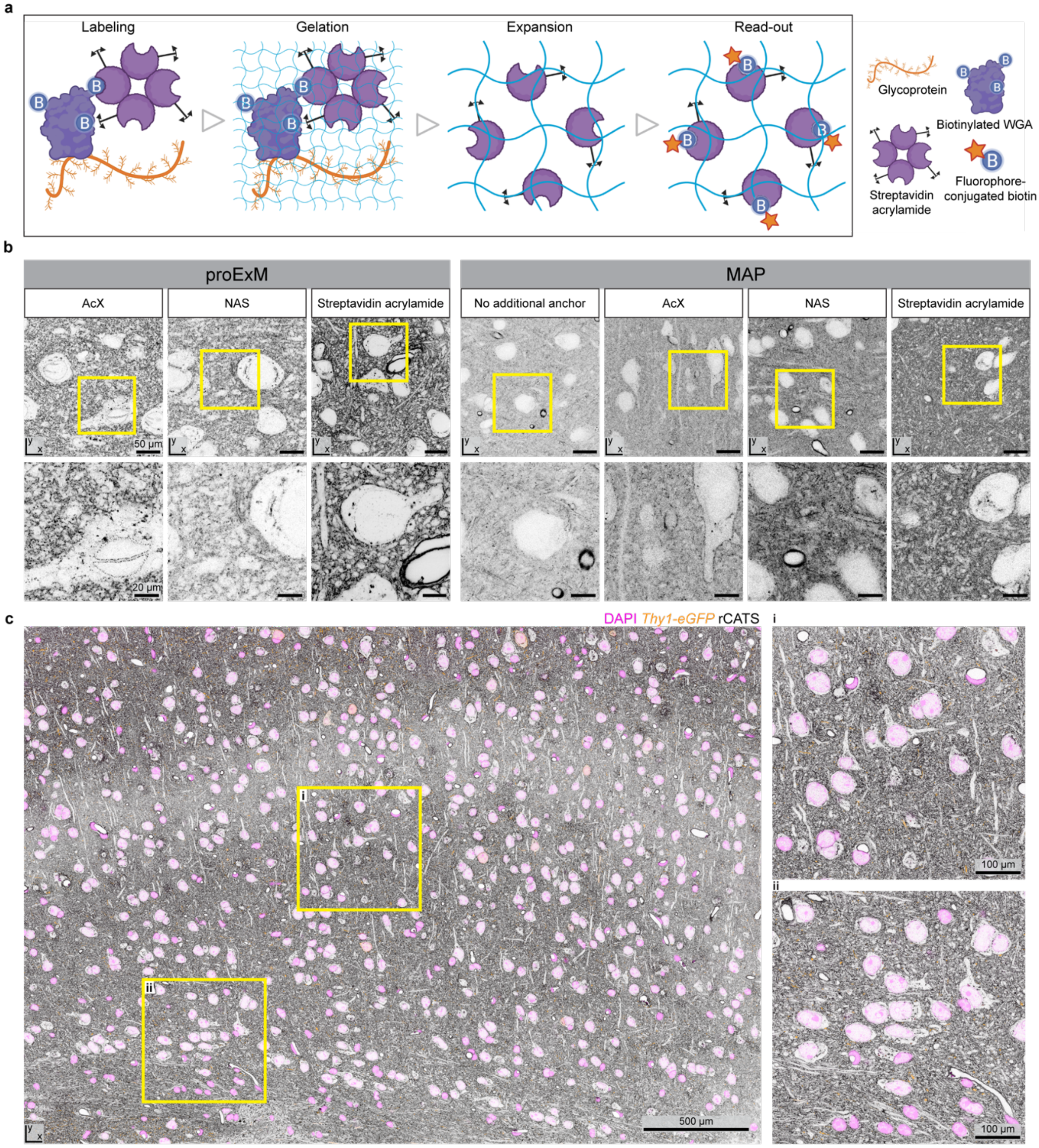

Supplementary Fig. 22| Pipeline for signal retention with rCATS in expansion microscopy. a, Schematic of rCATS expansion pipeline ensuring retention of WGA signal. Biotinylated WGA labels predominantly extracellularly located carbohydrate residues. To ensure that the WGA signal is retained in the hydrogel, the biotin on the WGA is targeted with streptavidin acrylamide. Upon gelation, streptavidin copolymerizes with the hydrogel via the acrylamide moiety. During the expansion procedure, polysaccharides and biotinylated WGA may get lost, but the anchored streptavidin remains in place and is read out with fluorophore-conjugated biotin post-expansion. Streptavidin does retain its capacity to bind biotin after common homogenization procedures for disrupting tissue cohesiveness in ExM, including heat/chemical denaturation and enzymatic digestion. **b,** Retention of biotinylated WGA in slices from PFA-perfused mouse brain was tested with two common ExM strategies, protein-

retention ExM (proExM) and magnified analysis of proteomes (MAP), using various retention strategies. Anchoring for proExM with acrylic acid N-hydroxysuccinimide ester (NAS) or Acryloyl-X (AcX) led to specific, but low intensity signal, likely due to the limited number of lysine residues on WGA that can be targeted by such an approach. In contrast, a highly specific and strong WGA labeling pattern was obtained when retaining WGA via streptavidin acrylamide and reading it out post-expansion with fluorophore-conjugated biotin. With the standard PFA/acrylamide-based retention strategy in the MAP approach (no additional anchoring), the WGA signal was grainy and diffuse. Few structures, mainly blood vessels, were labeled strongly. Additional anchoring with AcX or NAS in MAP somewhat improved WGA-retention, but still resulted in an overall diffuse labeling pattern with strong labeling of blood vessels and putative myelinated processes. Diffuse labeling was also visible in cell bodies. Handing over WGA signal to streptavidin acrylamide improved the labeling quality further in MAP, but still resulted in inhomogeneities and aggregates, such that we opted for the proExM approach in this specific case. **c,** A slab of an adult *Thy1-eGFP* mouse cortex processed with the rCATS expansion pipeline, 4-fold expanded with proExM. (i-ii) Zoom-ins of the yellow boxed regions. Scale bars refer to sample size after expansion throughout. All images were acquired with a confocal microscope. Test experiments with the various anchoring compounds (panel b) were performed once for each of the two expansion protocols, with and without anchors. Higher labeling intensity upon anchoring with streptavidin acrylamide was confirmed twice, i.e. in a total of n=3 biological replicates each for proExM and MAP.

Supplementary Fig. 23

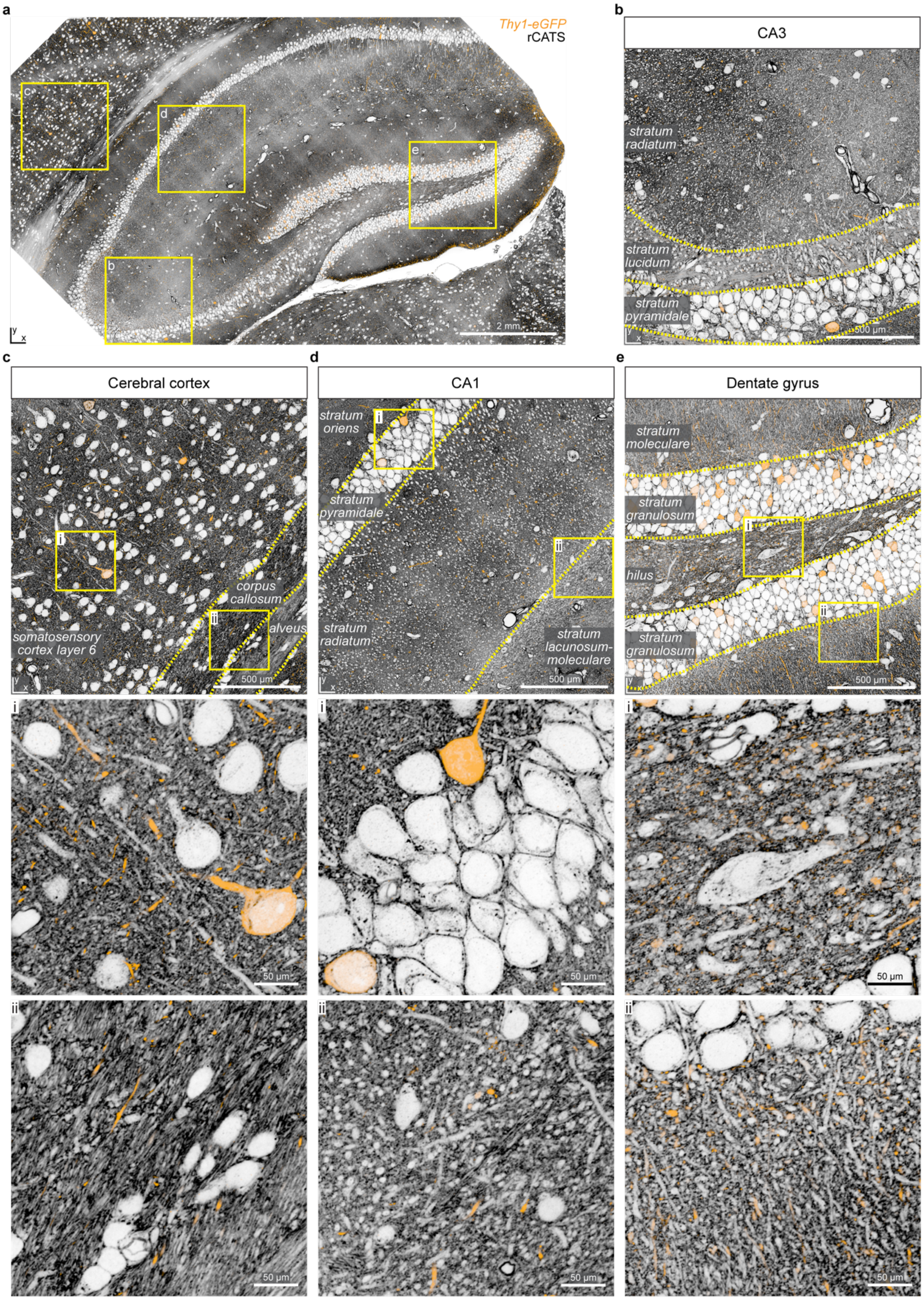

Supplementary Fig. 23| Nervous tissue organization across scales revealed by rCATS and expansion microscopy. Hippocampus and overlying cortex labeled with rCATS and expanded

~4-fold with protein retention ExM in a brain of an adult, perfusion-fixed *Thy1-eGFP* mouse, imaged with a spinning disc confocal microscope. A sparse subset of neurons is highlighted by cytosolic eGFP expression. Same dataset as in Fig. 5e. **a,** Overview image of a coronal section of hippocampus and adjacent cortex. Boxes indicate the regions enlarged in the following panels. Scale bars refer to sample size after expansion throughout. **b**, Enlarged view of the CA3 region (same as in Fig. 5e). Hippocampal layers, including the CA3 stratum radiatum, CA3 stratum lucidum, and the CA3 pyramidal layer (stratum pyramidale) are identifiable from the rCATS labeling. **c,** Enlarged view of the cerebral cortex with two further zoom-ins as indicated by the boxes. The somatosensory cortex layer 6 can be distinguished from the corpus callosum and alveus by the differential organization of the tissue. The cortex contains many cell bodies and processes running perpendicular to the cortical surface (zoom i), whereas the white matter shown here mainly consists of fiber tracts running parallel to the cortical surface (zoom ii). Sparse eGFP-positive cell bodies and neuronal processes can be found. **d,** Enlarged view of the CA1 area and two further zoom-ins as indicated by the boxes. The CA1 stratum oriens, CA1 stratum pyramidale, CA1 stratum radiatum, and CA1 stratum lacunosum-moleculare can be identified. **e,** Enlarged view in the dentate gyrus and two further zoom-ins. The organization of the DG blades, including the DG granule cell layer (stratum granulosum) surrounding the DG polymorph layer (hilus), as well as the DG molecular layer (stratum moleculare), can be appreciated. In the polymorph and molecular layers, sparse eGFP-positive structures, predominantly corresponding to DG axons and boutons, are visible. Whole coronal brain slice expansion in combination with proExM was performed in 6 samples (n=6 technical replicates) across n=4 animals. The representative imaging data displayed here was acquired in a single specimen.

Supplementary Fig. 24

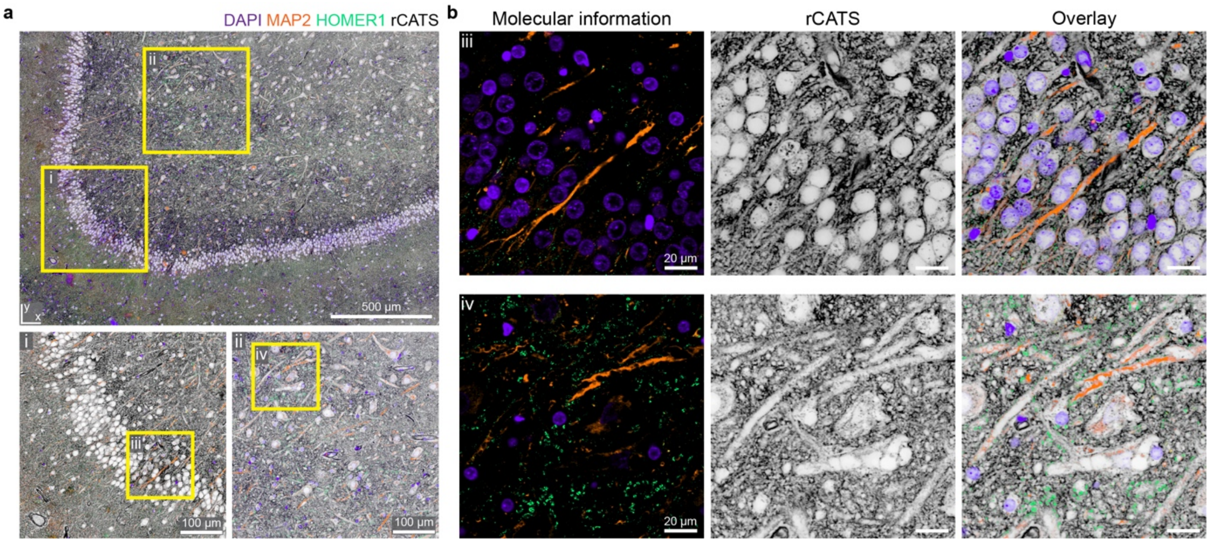

Supplementary Fig. 24| Tissue architecture in human brain surgery specimen. a, rCATS (gray, WGA-CF633) in the DG-region of a human hippocampal surgery specimen (36-year old male patient undergoing surgery for epilepsy treatment) with additional immunostaining for dendrites (MAP2, orange), excitatory synapses (HOMER1, green) and nuclei (DAPI, purple). (*Top*) Confocal, with magnified views of boxed regions (*bottom*). The sample was immersion fixed and stored in cryoprotectant at -80°C until use (see Methods). **b,** Magnified views of the boxed regions iii and iv in a. (*Left*) Molecular stainings alone, (*middle*) tissue architecture revealed by rCATS, (*right*) overlay. Raw data. rCATS imaging in surgery explants was performed in n=8 epilepsy patients, from which we selected the samples in Fig. 6a-c and this figure for quality of structural representation.

Supplementary Fig. 25

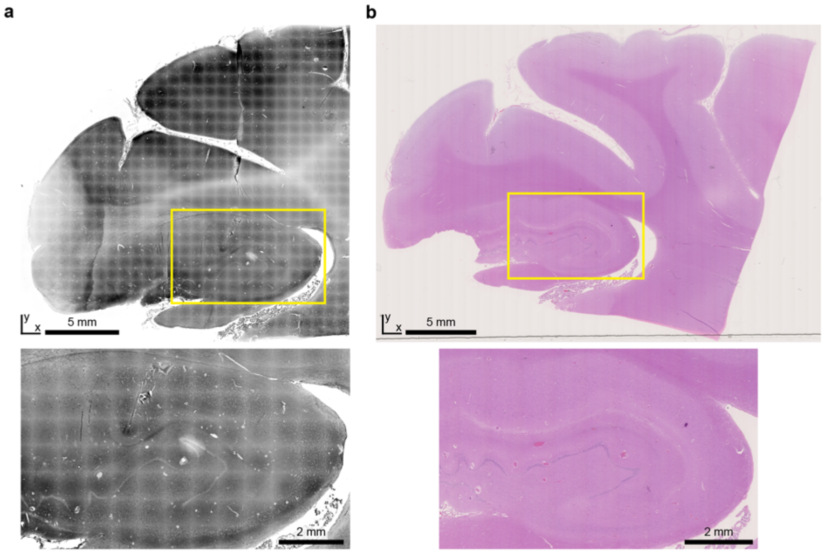

Supplementary Fig. 25| Gross overview in human postmortem brain. a, Confocal tile scan overview in rCATS (*top*, same data as in Fig. 6d) and magnified view of the hippocampal region (*bottom*). **b,** Hematoxylin and Eosin staining of a nearby tissue slice in the same specimen, differentiating gray matter and white matter. Data from a 35-year old female patient without brain pathology. Data are representative of rCATS imaging in n=5 brain sections obtained across n=2 autopsy specimens, of which the displayed sample featured better structural preservation. Comparison with H&E staining was performed once.

Supplementary Fig. 26

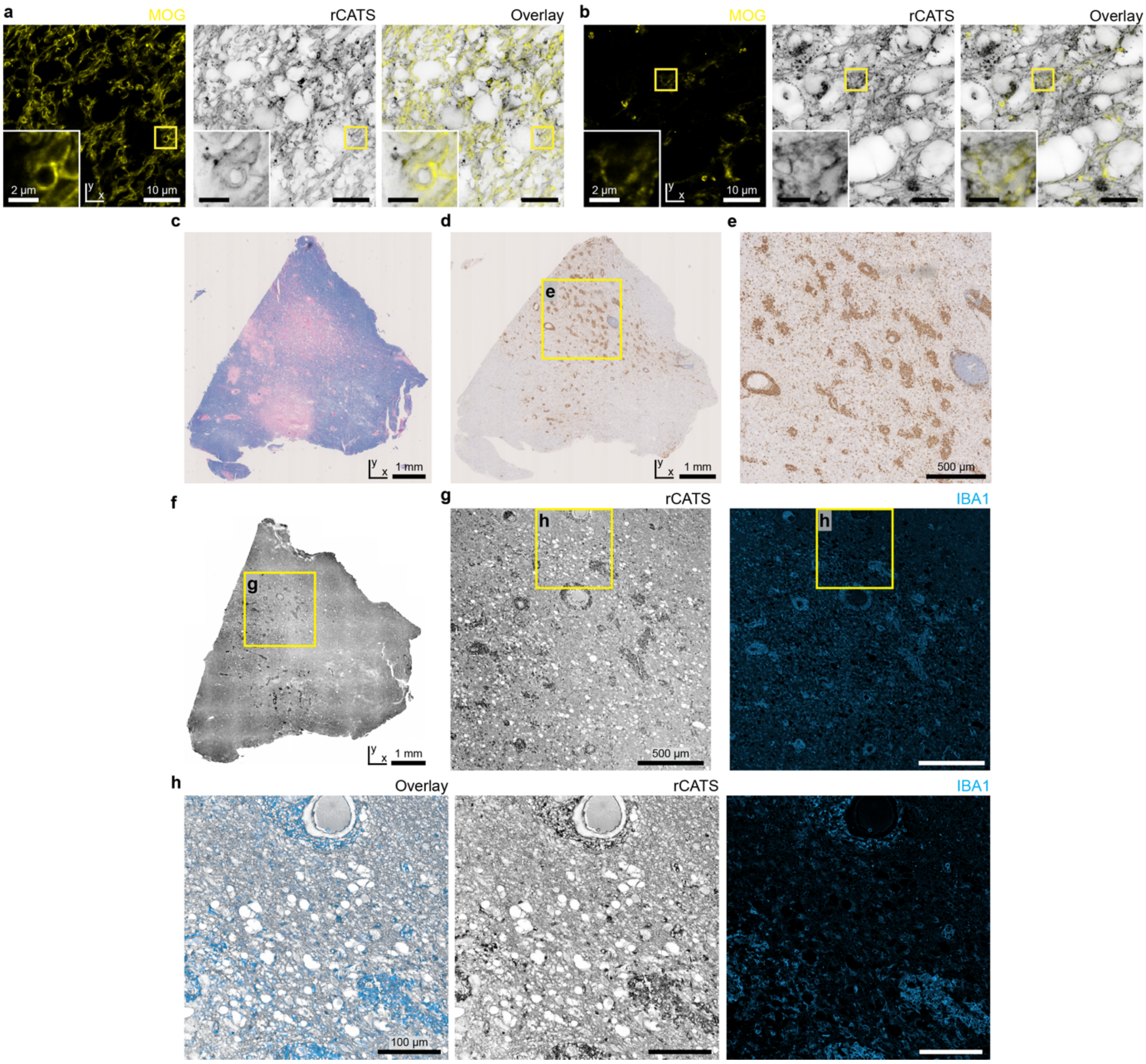

Supplementary Fig. 26| Tissue architecture visualized by rCATS in an archival FFPE specimen from a patient diagnosed with MOGAD. a, rCATS (gray, *xy*-STED, WGA-CF633) combined with immunolabeling for a component of myelin sheaths, MOG (myelin oligodendrocyte glycoprotein, yellow, confocal, AF488) in a region where myelin is at least partially preserved, as exemplified by the putative myelinated axon in the inset, showing an intact perimeter and an ad-axonal line at the inner border of the myelin sheath in rCATS. Same specimen from a 53-year old female patient as in Fig. 6e. **b,** Different region from the same section imaged as in panel a. Absence of MOG labeling indicates a high degree of demyelination in this region. Only remnants of myelinated axons are present. **c,** Nearby section of the same paraffin tissue block with Luxol Fast Blue staining. Pink areas are indicative of demyelination, consistent with the MOG immunolabeling pattern in Fig. 6e. **d,e,** Immunohistochemistry for the macrophage marker CD68 (brown, diaminobenzidine staining imaged on slide scanner) on a nearby section of the same tissue block with magnified view according to the yellow box. Macrophages are prominently present around blood vessels. The blood vessel on the right margin of panel e shows a predominantly lymphocytic infiltrate (CD4 positive, data not shown). **f,** Confocal image of tissue section from the same specimen labeled with rCATS (WGA-CF633). **g,** Higher resolution confocal scan in the region indicated in f,

combined with immunolabeling for IBA1 (ionized calcium-binding adapter molecule 1, blue, AF594), expressed in microglia and macrophages. **h,** Confocal scan in the region indicated by the box in panel g. Dark labeling in rCATS within $IBA1^+$ cells is consistent with the accumulation of carbohydrate containing myelin degradation products. Imaging was performed for one patient with MOGAD (*n*=1) on 3 brain sections (*n*=3 technical replicates) for the rCATS labeling in combination with immunostaining. Comparison with Luxol Fast Blue and CD68 staining were performed once.

Supplementary Fig. 27

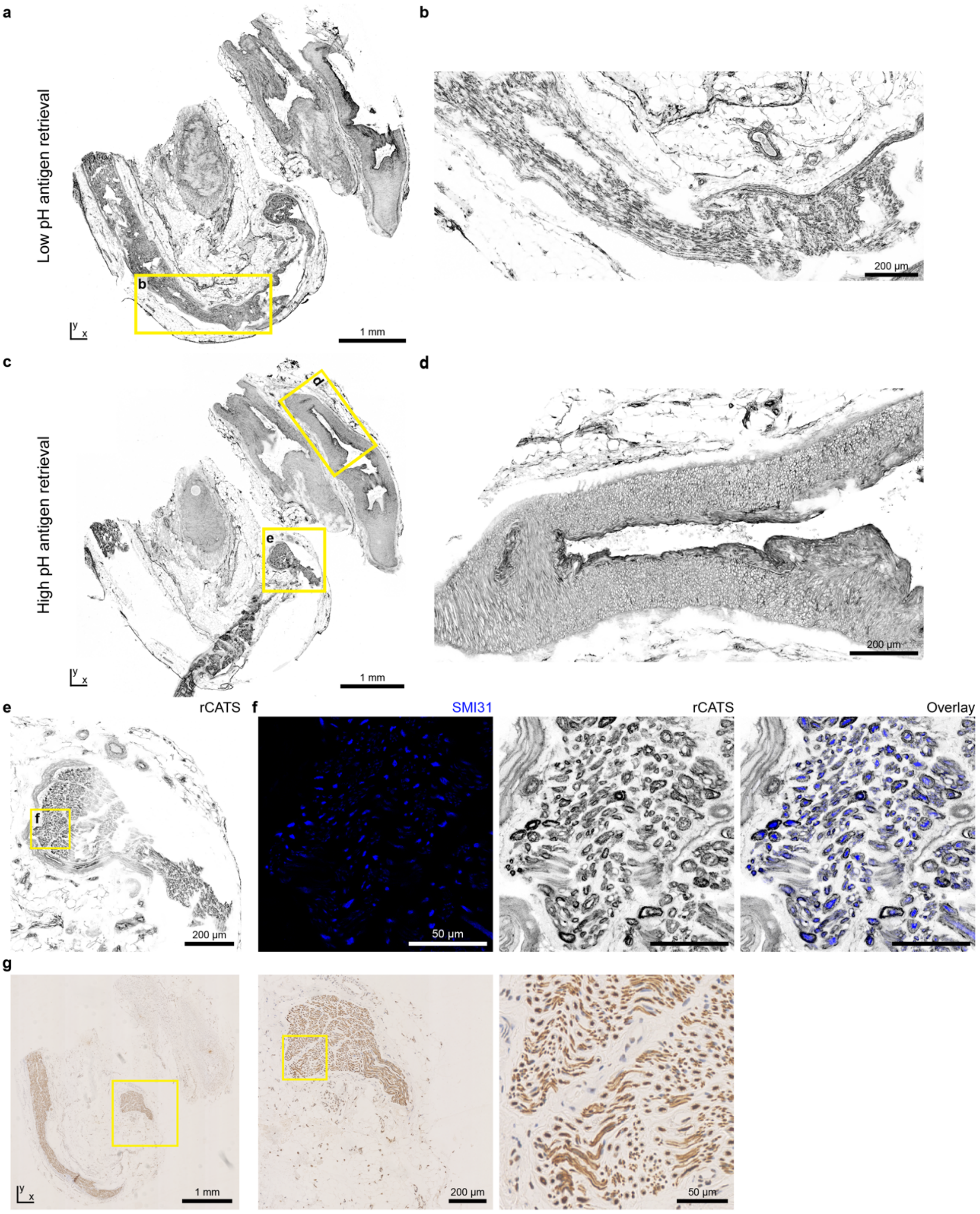

Supplementary Fig. 27| Human peripheral nerve visualized with rCATS. a, rCATS confocal overview of an FFPE specimen of a biopsy of the sural nerve collected for histopathological evaluation of peripheral neuropathy from a 44-year old female patient. Various tissue types, including the sural nerve passing through the yellow rectangle, blood vessels, and connective/fatty tissue are readily discerned. Portions of the nerve were disrupted during sample preparation. To test compatibility of rCATS with standard antigen retrieval protocols required for immunolabelings in FFPE tissues, the specimen was subject to a low pH (citric acid based) antigen retrieval protocol prior to labeling with WGA-CF633. **b,** Higher magnification confocal image as indicated by the box in panel a, showing portions of the nerve

embedded in the surrounding tissue. **c,** Nearby section of the same tissue block as in panel a, but treated with a high pH antigen retrieval protocol. **d,** Confocal scan of the area indicated by the yellow rectangle in panel c, showing the cellular architecture of an artery. **e,** Confocal rCATS image of the portion of the nerve indicated in panel c. **f,** Immunolabeling for the axonal marker Neurofilament H (blue, confocal) together with rCATS (confocal, gray, WGA-CF633) in the region indicated in panel e. Neurofilament H labeling highlights the central axon cylinder in these myelinated nerve fibers (compare also Fig. 6k). **g,** Immunohistochemistry for Neurofilament H in a nearby tissue section of the same specimen, imaged on a slide scanner. rCATS labeling in combination with immunostaining of a human peripheral nerve biopsy was performed for one patient (n=1, same as in Fig. 6j,k) with n=2 technical replicates (2 sections). Comparison with Neurofilament H staining (panel g) was performed once.

3 References

1. Dorkenwald, S. *et al.* Automated synaptic connectivity inference for volume electron microscopy. *Nature Methods* **14**, 435–442 (2017).
2. Staffler, B. *et al.* SynEM, automated synapse detection for connectomics. *eLife* **6**, e26414 (2017).
3. Turner, N. L. *et al.* Reconstruction of neocortex: Organelles, compartments, cells, circuits, and activity. *Cell* **185**, 1082–1100 (2022).
4. Scheffer, L. K. *et al.* A connectome and analysis of the adult Drosophila central brain. *eLife* **9**, e57443 (2020).
