# Supplementary material for: Imaging brain tissue architecture across millimeter to nanometer scales
Source: Nat Biotechnol. 2023 Aug 31;42(7):1051–64. doi: 10.1038/s41587-023-01911-8 (PMC11252008; doi:10.1038/s41587-023-01911-8)
Supplement: Supplementary file 2 — Reporting Summary [file 41587_2023_1911_MOESM2_ESM.pdf]

nature portfolio

Corresponding author(s): Johann G. Danzl
Last updated by author(s): Aug 12, 2023

Reporting Summary

Nature Portfolio wishes to improve the reproducibility of the work that we publish. This form provides structure for consistency and transparency in reporting. For further information on Nature Portfolio policies, see our Editorial Policies and the Editorial Policy Checklist.

Statistics

For all statistical analyses, confirm that the following items are present in the figure legend, table legend, main text, or Methods section.

n/a	Confirmed	
☐	☒	The exact sample size (n) for each experimental group/condition, given as a discrete number and unit of measurement
☐	☒	A statement on whether measurements were taken from distinct samples or whether the same sample was measured repeatedly
☐	☒	The statistical test(s) used AND whether they are one- or two-sided<br>*Only common tests should be described solely by name; describe more complex techniques in the Methods section.*
☒	☐	A description of all covariates tested
☒	☐	A description of any assumptions or corrections, such as tests of normality and adjustment for multiple comparisons
☐	☒	A full description of the statistical parameters including central tendency (e.g. means) or other basic estimates (e.g. regression coefficient) AND variation (e.g. standard deviation) or associated estimates of uncertainty (e.g. confidence intervals)
☐	☒	For null hypothesis testing, the test statistic (e.g. F, t, r) with confidence intervals, effect sizes, degrees of freedom and P value noted<br>*Give P values as exact values whenever suitable.*
☒	☐	For Bayesian analysis, information on the choice of priors and Markov chain Monte Carlo settings
☒	☐	For hierarchical and complex designs, identification of the appropriate level for tests and full reporting of outcomes
☐	☒	Estimates of effect sizes (e.g. Cohen's d, Pearson's r), indicating how they were calculated

Our web collection on statistics for biologists contains articles on many of the points above.

Software and code

Policy information about availability of computer code

Data collection

Data were collected using the following instruments and softwares: Abberior Instruments Expert Line STED microscope using Imspector software (version 14.0.3052 or 16.3.13031); Leica Sp8 using Leica LAS X software (version 2.5.7.23225); Andor Dragonfly 505 using Andor Fusion Software (version 2.2). Electrophysiology signals were acquired using Signal 6.0 software (Cambridge Electronic Design). Pathology slides were digitized on a NanoZoomer 2.0-HT digital slide scanner C9600 (Hamamatsu Photonics, Hamamatsu, Japan).

Data analysis

Visual inspection of the imaging data was performed with ImageJ/Fiji (version 1.53f51) or Napari (version 0.4.12, https://doi.org/10.5281/ZENODO.3555620). Multi-channel data overlays were produced with GIMP (version 2.10.30).
Image analysis was performed in ImageJ/Fiji including Bioformats, Calculator Plus, Grid/Collection stitching, BigStitcher (v0.8.3) and BigWarp plugins. Deep-learning-based image denoising was done with Noise2Void (version 0.2.1) installed from GitHub (https://github.com/juglab/n2v).
Manual segmentation and proof-reading of segmentation data were performed in VAST (version 1.4.0), downloaded from https://lichtman.rc.fas.harvard.edu/vast/.
Custom scripts written with Python (version 3.7.12) and implemented with Jupyter lab (version 3.2.4) were used for automated segmentation of pSCRs. Models for deep-learning-based prediction of synapse location were trained with a U-Net convolutional neural network based on code adapted from GitHub (https://github.com/Li-En-Good/VISTA). Blender 2.92 (blender.org) was used for processing steps in quantification and for visualization.
Skeletonization of neuronal arborization from expanded samples was performed with webKnossos (version v22.05.1) installed on a local server after conversion to webKnossos file data structure with custom scripts based on the webKnossos Python library (v0.10.5). Tracing of axons from super-resolved coCATS data was also performed with webKnossos by an experimenter who was blinded for the sparse positive channel. Evaluation was performed by a second experimenter guided by the positive channel.
Electrophysiology recordings were analyzed by Stimfit (Front. Neuroinform 8, 16 (2014)) and Matlab based scripts.
GraphPad Prism (version 9.0.2) was used for statistical tests. GraphPad Prism and Excel 2016 were used to create graphs. Schematics were created with BioRender (biorender.com).

Pathology slide images were exported to tif format with the NPD.Viewer2 software (Hamamatsu).
Code related to this publication is available at https://github.com/danzllab/CATS.

For manuscripts utilizing custom algorithms or software that are central to the research but not yet described in published literature, software must be made available to editors and reviewers. We strongly encourage code deposition in a community repository (e.g. GitHub). See the Nature Portfolio guidelines for submitting code & software for further information.

Data

Policy information about availability of data

All manuscripts must include a data availability statement. This statement should provide the following information, where applicable:

- Accession codes, unique identifiers, or web links for publicly available datasets
- A description of any restrictions on data availability
- For clinical datasets or third party data, please ensure that the statement adheres to our policy

Source data are available at the Institute of Science and Technology Austria's data repository with DOI: www.doi.org/10.15479/AT:ISTA:13126 (https://research-explorer.ista.ac.at/record/13126).

Field-specific reporting

Please select the one below that is the best fit for your research. If you are not sure, read the appropriate sections before making your selection.

☒ Life sciences ☐ Behavioural & social sciences ☐ Ecological, evolutionary & environmental sciences

For a reference copy of the document with all sections, see nature.com/documents/nr-reporting-summary-flat.pdf

Life sciences study design

All studies must disclose on these points even when the disclosure is negative.

Sample size

As this is a proof-of-concept study focusing on the development of new technology and its applicability, no prior determination of sample size was performed. Once the concept was proven and the data quality to our satisfaction, experiments were performed in multiple replicates to ensure and demonstrate reproducibility.
The sample size for MFB characterization from coCATS data (n=30) was chosen to reflect the complexity of the biological system and mirror the diversity of connectivity and structure at the single synapse level. Number of reconstructed boutons was limited by the required human time for manual segmentation. 2 MFBs from one rCATS data set were exemplarily segmented to demonstrate feasibility.
The sample size for the different components of the neuronal input field was given by the number of synaptically connected structures within the chosen imaging volume. We reconstructed 58 distinct cellular structures, 43 of which were MFBs as identified by connectivity and morphology. The reconstructed dendrite segment contained 68 subsegments identified as dendritic spines.
The amount of training data for deep-learning-based models for pSCR indentification affects the accuracy of the prediction. We stopped collecting training data, when increasing the amount of training data did not substantially increase the accuracy of the prediction.

Data exclusions

Some datasets displayed were cropped from larger raw versions to focus on specific regions of interest.
For analysis of MFB spine connectivity in relation to the synaptic input field, 3 out of 43 MFBs were excluded, as they were only partially located within the imaged volume. For high quality representation of tissue structure, optimum tissue preservation, labeling and imaging conditions are required. We discarded datasets that were of lower quality.

Replication

Stated replicates give a lower bound how many times individual experiments were performed with similar results. As this manuscript reports on a technological development, a large number of experiments with some variation of parameters have been performed, including during the development phase. For analysis, only datasets of high labeling and imaging quality were pursued.
In all images, representative data from single experiments are shown. To confirm reproducibility of the technology, we performed a series of technical replicates which were typically recorded across several biological specimens, as indicated below. For some of the procedures that we performed routinely, such as in vivo microinjection, the stated number of replicates gives a lower bound and we did not count additional replicates beyond n=10.
Individual datasets were replicated as follows:
Figure 1: Fig. 1b: Data are representative of coCATS experiments in n=10 organotypic hippocampal slices and rCATS in n=10 fixative perfused animals. Fig. 1c,d,e: Images are representative of coCATS with in vivo microinjection into the LV in n=10 animals. Figure 2: Fig. 2a: Imaging data are representative of in vivo microinjection into the LV in n=10 animals. Fig. 2b-g: Renderings and quantitative analysis of n_MFB=30 MFBs reconstructed (10 from each of 3 imaging volumes recorded across two brain sections (one animal); 22 MFBs are displayed in Fig. 2b and 8 MFBs in Supplementary Fig. 7. Fig. 2h represents one of the 3 imaging volumes used for MFB visualization and quantification. Fig. 2j,k: Training was performed on n=13 imaging volumes recorded across 4 brain sections from n=3 animals, and testing on n=1 dataset. Figure 3: Fig. 3a,b: Imaging data are representative of coCATS in n=10 organotypic slices. Fig. 3c-g: Data are representative of coCATS labeling in combination with functional recordings and dye-filling of various cell types in n=6 organotypic slices. Fig. 3h,i: 3D-reconstruction was performed for n=1 specimen and analysis in Fig. 3j-l comprised one dendrite with n_spine=68 spine structures, and n_MFB=40 MFBs. 3 additional MFBs were only partially contained within the imaging volume and thus not included in quantifications. Additionally, 14 non-MFB structures in synaptic contact with the dendrite were reconstructed. Figure 4: Fig. 4a-e: coCATS labeling in combination with functional recordings is representative of experiments in n=6 organotypic slices. Following the axon trajectory with 3D-reconstruction was done for n=1 sample, with bouton characteristics extracted from a total of N_analyzed=17 boutons imaged across multiple volumes along the axon trajectory. Reconstructions were performed on 2 imaging volumes, as seen in Fig. 4d,e. Fig. 4f: coCATS images represent raw data from n=5 brain slices obtained from n=2 independent biological specimens with in vivo microinjection into LV and primary motor cortex, respectively. They are representative of coCATS in vivo microinjection in n=10 and n=4 animals for LV and cortical microinjection, respectively. Figure 5: Fig. 5a,b: Data are representative of rCATS in n=10 perfusion-fixed specimens. Fig. 5c: rCATS/coCATS co-labeling was performed in n=7 brain

sections (technical replicates) across n=3 animals with various fluorophore combinations. Fig. 5d: Data are representative of coCATS with MAP in n=3 organotypic slices. Fig. 5 e-i: Whole-section rCATS with proExM was performed in 6 brain slices across n=4 animals and skeletonization in n=1 dataset. Figure 6: Fig. 6a-c: rCATS was performed on surgery explants from n=8 patients and the best-preserved specimens were selected for display here and in Supplementary Fig. 24. Fig. 6d: rCATS was performed in 5 slices from autopsy specimens of n=2 individuals. Fig. 6e-i: Data representative of n=3 technical rCATS replicates from n=1 patient with MOGAD. Fig. 6j,k: rCATS data representative of n=2 technical replicates in peripheral nerve biopsy of n=1 patient. Extended Data Fig. 1: Comparison of confocal vs. STED performance in coCATS-labeled specimens was performed in n=3 biological specimens in 3 independent imaging sessions. Imaging of fluorescent beads is representative for typical microscope performance and was acquired in one imaging session. Extended Data Fig. 2: Displayed data are from a single dataset representative of coCATS with in vivo microinjection into the lateral ventricle performed in n=10 animals. Extended Data Figs. 3,4: Tests for pSCR location relative to synaptic markers in Extended Data Figures 3 and 4 were performed for a total of 10 markers across 17 brain slices from n=6 animals. Extended Data Fig. 5: Reconstruction was performed on n=1 dataset (same as Fig. 3g), including the positively labelled dendrite with spines (n_spines=68), MFBs (n_MFBs=43) with axons and filopodia (n_axons/filopodia=38), and structures in synaptic contact with the main dendrite, not identifiable as MFB-related structures (n_non-MFB=14). Extended Data Fig. 6: CoCATS in vivo microinjection into the lateral ventricle was performed in n=10 biological specimens. Astrocyte 3D-reconstruction was performed once. Extended Data Fig. 7: Imaging data is representative and was acquired across 8 different brain sections from n=3 individual biological specimens. coCATS labeling of various brain regions was achieved by in vivo microinjection into the lateral ventricle or cortex, which was performed in n=10 and n=4 biological specimens, respectively. Extended Data Fig. 8: Serial imaging data in panel a are from a single specimen and data in panel b were acquired across brain slices from n=6 biological specimens.

Extended Data Fig. 9: coCATS-rCATS co-labeling was performed in 7 brain sections total from n=3 animals with different fluorophore combinations. Extended Data Fig. 10: Imaging data are representative of coCATS labeling in n=5 human cerebral organoids cultured at 3 different time points. Dense manual reconstruction was performed in one dataset. Suppl. Fig. 1: All probes which were used for subsequent routine experiments, i.e. STAR RED NHS, ATTO643 NHS and NHS-PEG12-biotin, were tested three times in organotypic slice cultures from different culture time points (n=3 biological specimens). All other probes were tested in n=2 biological specimens, except for AF546 NHS, AF594 NHS and maleimide-PEG11-biotin. AF546 NHS and AF594 NHS were tested only once, as the staining pattern matched the pattern of other NHS-conjugated fluorophores. Maleimide-PEG11-biotin was tested only once, as the result matched the labeling pattern of Atto643 maleimide. Suppl. Fig. 2: Serial whole-brain sectioning and overview imaging of the dye-distribution in the brain after LV injection was performed in n=5 animals. Injection of coCATS label into the LV and imaging as described for the various datasets throughout the manuscript were performed in n=10 animals. Suppl. Fig. 3: Training N2V networks in independent N2V runs to obtain n=5 technical replicates for the same volumetric dataset was done in n=1 specimen. Suppl. Fig. 4: The data displayed are representative comparisons of raw vs. denoised imaging data as displayed in the main figures and were recorded across n=5 biological specimens. Suppl. Fig. 5: Displayed images show representative examples of automated and proofread pSCR segmentations. coCATS in vivo microinjection into the lateral ventricle for labeling CA3 stratum lucidum was performed in n=10 biological specimens. Suppl. Fig. 6: Comparison of confocal vs. STED performance in coCATS-labeled specimens is representative of imaging in n=3 biological specimens. It is furthermore representative of the improved tissue visualization with xy- or z-STED imaging over diffraction-limited imaging in a number of measurements throughout the manuscript, recorded across multiple biological specimens (see e.g. Fig. 1c, Fig. 4f, Fig. 5a, Extended Data Fig. 1,3,4,7,8,9). The illustration experiment in panel g was done in n=1 sample and is representative of routinely setting the correction collar to the desired imaging depth in our (STED) imaging experiments. The correction collar was set to 0.17 once and imaged. The other values were set and imaged twice. Suppl. Fig. 7: Panel a shows eight reconstructed MFBs representing together with the 22 reconstructed boutons in Fig. 2b the total of 30 MFBs quantified in Fig. 2c-g (n_MFB=30, with 10 selected from each of 3 imaging volumes, recorded across two brain sections (n=2 technical replicates) from one animal (n=1 biological replicate)). Analysis in panel b was performed on the n_MFB=30 reconstructed MFBs. Suppl. Fig. 8: CoCATS in vivo microinjection into the lateral ventricle as used here was performed in n=10 biological specimens. The N2V-deep-learning (DL) model in panels a-d,f,i was trained on n=13 denoised volumetric imaging datasets recorded across 4 brain sections coming from 3 animals (n=3 biological replicates). The raw-DL model in panels c,d was trained on the same datasets without denoising. The DL model trained on confocal BASSOON (panel e) was trained on n=8 volumetric imaging datasets recorded across 3 brain slices from 2 animals. The training data for the DL model based on STED-BASSOON in panel e was size-matched to training on confocal BASSOON and consisted of n=9 volumetric imaging datasets recorded across 4 brain slices from 3 animals. Suppl. Fig. 9: coCATS labeling in combination with functional recordings and dye-filling of various cell types was performed in 6 organotypic brain sections (n=6 biological specimens). Suppl. Fig. 10: Measurements in panel a were performed in cultures prepared at three different time points and comprised 11 control cells recorded across 3 slices (n=11 cells), and 9 cells recorded across 4 dye-exposed slices (STAR RED-NHS, n=9 cells). Electrophysiological recording during dye-incubation (panel b) was performed in n=3 biological specimens. Suppl. Fig. 11: CoCATS labeling in combination with functional recordings and dye-filling of various cell types was performed in 6 organotypic brain sections (n=6 biological specimens). The data stems from a single imaging volume (same as Fig. 3g-i). Suppl. Fig. 12: coCATS labeling in combination with functional recordings and dye-filling of various cell types was performed in 6 organotypic brain sections (n=6 biological specimens). All boutons positively labelled here belong to a single cell (same as Fig. 4a-d) and were acquired across multiple imaging volumes along the axon in the same organotypic slice (n=1 biological specimen). Suppl. Fig. 13: Same dataset as in Fig. 4e. coCATS labeling in combination with functional recordings and dye-filling of various cell types was performed in 6 organotypic brain sections (n=6 biological specimens). Suppl. Fig. 14: Tracing was performed in n=10 axons from one imaging volume in n=1 biological specimen. Suppl. Fig. 15: The data displayed were acquired from the same biological specimen. coCATS in vivo microinjection in the cortex was performed in 4 animals (n=4 biological specimens). Suppl. Fig. 16: coCATS combined with myelin labeling was performed in 2 brain sections (n=2 technical replicates) from one biological specimen. Suppl. Fig. 17: After initial screening, involving n=3 biological replicates for WGA, this lectin was used for further experiments. The other lectins and HABP were not further pursued after testing in n=1 brain section each. Suppl. Fig. 18: rCATS in combination with sparse Thy1-eGFP labeling was performed in n=3 biological specimens. rCATS in combination with Fluoromyelin labeling in panel d was performed on 4 brain slices across n=3 animals. Suppl. Fig. 19: rCATS in perfusion-fixed brain slices was performed in n=10 biological specimens. Exemplary MFB segmentation from rCATS data was performed for 2 MFBs from one imaging volume. Suppl. Fig. 20: rCATS labeling in perfused brain slices, as seen in panel a, was performed in n=10 biological specimens. rCATS labeling of immersion-fixed half-hemispheres was performed once. The effect of permeabilization conditions on rCATS and antibody labeling depth (panels b-d) was tested twice, in n=2 independent biological specimens. Suppl. Fig. 21: coCATS labeling of organotypic brain sections in combination with MAP (panels a,b) was performed in n=3 biological specimens. coCATS labeling of organotypic brain sections in combination with proExM (panels c,d) was performed in n=3 biological specimens. Suppl. Fig. 22: Test experiments with the various anchoring compounds (panel b) were performed once for each of the two expansion protocols, with and without anchors. Higher labeling intensity upon anchoring with streptavidin acrylamide was confirmed twice, i.e. in a total of n=3 biological replicates each for proExM and MAP. Suppl. Fig. 23: Whole coronal brain slice expansion in combination with proExM was performed in 6 samples (n=6 technical replicates) across n=4 animals. The representative imaging data displayed here was acquired in a single specimen. Suppl. Fig. 24: rCATS imaging in surgery explants was performed in n=8 epilepsy patients, from which we selected the samples in Fig. 6a-c and Suppl. Fig. 24 for quality of structural representation. Suppl. Fig. 25: Data are representative of rCATS imaging in n=5 brain sections obtained across n=2 autopsy specimens, of which the displayed sample featured better structural preservation. Comparison with H&E staining was performed once. Suppl. Fig. 26: Imaging was performed for one patient with MOGAD (n=1) on 3 brain sections (n=3 technical replicates) for the rCATS labeling in combination with immunostaining.

	Comparison with Luxol Fast Blue and CD68 staining were performed once. Suppl. Fig. 27: RCATS labeling in combination with immunostaining of a human peripheral nerve biopsy was performed for one patient (n=1, same as in Fig. 6j,k) with n=2 technical replicates (2 sections). Comparison with Neurofilament H staining (panel g) was performed once.
Randomization	We do not compare samples between experimental groups. Accordingly, no randomization was performed.
Blinding	For evaluating axon traceability in Suppl. Fig. 14, the tracer was blinded to the eGFP ground truth data. For astrocyte segmentation in Extended Data Fig. 6, an experimenter first segmented the structure of a cell indicated by a seed point in the cell body purely from coCATS data. Afterwards, the experimenter was presented with the positive label and extended their segmentation of the cellular structure from coCATS data guided by th positive label. In all other experiments, no blinding was performed. Blinding was not relevant as we demonstrate a labeling/optical imaging development and do not compare experimental groups.

Reporting for specific materials, systems and methods

We require information from authors about some types of materials, experimental systems and methods used in many studies. Here, indicate whether each material, system or method listed is relevant to your study. If you are not sure if a list item applies to your research, read the appropriate section before selecting a response.

Materials & experimental systems

n/a	Involved in the study
☐	☒ Antibodies
☐	☒ Eukaryotic cell lines
☒	☐ Palaeontology and archaeology
☐	☒ Animals and other organisms
☐	☒ Human research participants
☒	☐ Clinical data
☒	☐ Dual use research of concern

Methods

n/a	Involved in the study
☒	☐ ChIP-seq
☒	☐ Flow cytometry
☒	☐ MRI-based neuroimaging

Antibodies

Antibodies used

Primary antibodies:
1. Anti-Bassoon monoclonal mouse (clone: SAP7F407, abcam, ab82958)
2. Anti-CD68 monoclonal mouse (clone: KP1, DAKO, M0814)
3. Anti-Gephyrin monoclonal mouse (clone: 3B11, Synaptic Systems, 147111)
4. Anti-glial fibrillary acidic protein monoclonal mouse (clone: 134B1, Synaptic Systems, 173011)
5. Anti-green fluorescent protein monoclonal, mouse (clone: 3E6, ThermoFisher Scientific/Invitrogen, A11120)
6. Anti-green fluorescent protein polyclonal, rabbit (ThermoFisher Scientific/Invitrogen, A11122)
7. Anti-Homer1 polyclonal, rabbit (Synaptic Systems, 160003)
8. Anti-ionized calcium binding adaport molecule 1 polyclonal rabbit (Wako Chemical/Fujifilm, 019-19741)
9. Anti-microtubule associated protein 2 polyclonal, guinea pig (Synaptic Systems, 188004)
10.Anti-Munc13-1 polyclonal guinea pig (Synaptic Systems, 126104)
11. Anti-myelin basic protein polyclonal mouse (Millipore, AB5864)
12. Anti-myelin oligodendrocyte glycoprotein monoclonal mouse (clone: CL2858, Atlas Antibodies, AMAb91067)
13. Anti-NEUN polyclonal guinea pig (Synaptic Systems, 266004)
14. Anti-Neurofilament H, phosphorylated (SMI31), monoclonal mouse (clone: SMI31, Covance SMI31P, BioLegend 801601)
15. Anti-Shank2 polyclonal, guinea pig (Synaptic Systems, 162204)
16. Anti-Synapsin 1/2 polyclonal guinea pig (Synaptic Systems, 106004)
17. Anti-Synaptobrevin 2 monoclonal mouse (clone: 69.1, Synaptic Systems, 104211)
18. Anti-Synaptophysin 1 monoclonal, mouse (clone: 7.2, Synaptic Systems, 101011)
19. Anti-Synaptophysin 1 polyclonal, guinea pig (Synaptic Systems, 101004)
20. Anti-vesicular gamma-aminobutyric acid transporter polyclonal, rabbit (Synaptic Systems, 131003)
21. Anti-vesicular glutamate transporter 1 polyclonal, rabbit (Synaptic Systems, 135302)

Secondary antibodies:
22. Alexa Fluor 488 donkey anti-rabbit IgG (H+L) (Jackson ImmunoResearch, 711-545-152)
23. Alexa Fluor 488 donkey anti-guinea pig IgG (H+L) (Jackson ImmunoResearch, 706-545-148)
24. Alexa Fluor 488 goat anti-mouse IgG (H+L) (ThermoFisher Scientific, A11001)
25. Alexa Fluor plus 488 goat anti-rabbit IgG (H+L) (ThermoFisher Scientific, A32731)
26. Alexa Fluor 546 goat anti-guinea pig IgG (H+L) (ThermoFisher Scientific, A11074)
27. Alexa Fluor 594 goat anti-guinea pig IgG (H+L) (ThermoFisher Scientific, A11076)
28. Alexa Fluor 594 goat anti-mouse IgG (H+L) (ThermoFisher Scientific, A11005)
29. Alexa Fluor 594 goat anti-rabbit IgG (H+L) (ThermoFisher Scientific, A11037)
30. Alexa Fluor 647 donkey anti-sheep IgG (H+L) (ThermoFisher Scientific, A21448)
30. STAR 580 goat anti-mouse IgG (abberior, ST580-1001)
31. STAR 580 goat anti-rabbit IgG (abberior, ST580-1002)

Validation

All antibodies were used for immunofluorescence staining of previously fixed mouse or human brain tissue. All stainings with the used antibodies yielded the expected staining patterns for their target structures within the super-resolved tissue context.
In addition, the following statements of validation were available from the manufacturers of the primary antibodies:
Antibody 1: positive control = rat brain tissue extract.

Antibody 2: The antibody was clustered as anti-CD68 at the Fourth International Workshop and Conference on Human Leucocyte Differentiation Antigens held in Vienna in 1989 (5). SDS-PAGE analysis of immunoprecipitates formed between the antibody and 125I-labeled lysates from human spleen with B-cell lymphoma rich in macrophages shows reaction with a 110 kDa polypeptide, corresponding to CD68 (4). In Western blotting of extracts of lung, spleen and U937 cells, diffuse 110, 70 and 40 kDa bands were detected when using reducing conditions. Under non-reducing conditions the spleen extract showed an additional 220 kDa band (4). See package insert for reference(s).
Antibody 3: Detects all splice variants that contain a complete E-domain including the C6 domain. K.O. validated. Reacts with human, rat, mouse, zebrafish.
Antibody 4: Specific for GFAP isoform 1 (alpha). K.O. validated. Reacts with human, rat, mouse, cow.
Antibody 5: this Antibody was verified by Relative expression to ensure that the antibody binds to the antigen stated.
Antibody 6: This Antibody was verified by Relative expression to esure that the antibody binds to the antigen stated.
Antibody 7: Specific for Homer 1. Cross-reactivity of the serum to Homer 2 and 3 was removed by pre-adsorption with Homer 2 (aa 1 - 176) and Homer 3 (aa 1 - 177). Reacts with human and mouse.
Antibody 8: cross-reactivity: human, mouse, rat and other.
Antibody 9: Specific for MAP 2; recognizes all four isoforms. Reacts with human, rat and mouse.
Antibody 10: K.O. validated. Reacts with rat, mouse, zebrafish.
Antibody 11: Recognizes Myelin Basic Protein in demyelinated nerve tissues. Immunohistochemistry analysis of lesioned rat spinal cord shows a high level of specificity for this antiserum. Reacts with human, rat.
Antibody 12: reacts with human, mouse, rat. Validated in 44 normal tissues and 20 cancers.
Antibody 13: reacts with rat and mouse.
Antibody 14: reacts with human, mouse, rat. Affinity purified.
Antibody 15: reacts with mouse. Specific for Shank2. K.O. tested. This antibody had been successfully used for the MAP expansion microscopy method.
Antibody 16: Specific for synapsins 1a/b and 2a/b. K.O. validated. Reacts with human, rat, mouse, hamster, cow, zebrafish.
Antibody 17: K.O. validated. Reacts with human, rat, mouse, hamster. No signal for chicken and zebrafish.
Antibody 18: Reacts with mouse. Specific for synaptophysin 1, no cross-reactivity to other synaptophysins. K.O. tested.
Antibody 19: Reacts with mouse. Specific for synaptophysin 1, no cross-reactivity to other synaptophysins.
Antibody 20: Reacts with mouse. Specific for VGAT. K.O. tested.
Antibody 21: Reacts with mouse. Specific for VGLUT 1. K.O. tested.

Eukaryotic cell lines

Policy information about cell lines

Cell line source(s)	For generation of human cerebral organoids, human H9 ES cells (WA09) were obtained from a commercial source (WA09, lot number: WIC-WA09-RB-001, WiCell). Generation of cerebral organoids from these cells was approved by the institutional ethics board (ISTA Ethics Committee, approval date June 09, 2020).
Authentication	Authentication was performed by the provider via short tandem repeat analysis, karyotype analysis (G-banding) and flow cytometry for embryonic stem cell markers. No further authentication was performed.
Mycoplasma contamination	Cells were routinely tested for mycoplasma contamination and were tested negative.
Commonly misidentified lines (See ICLAC register)	The study did not involve commonly misidentified cell lines.

Animals and other organisms

Policy information about studies involving animals; ARRIVE guidelines recommended for reporting animal research

Laboratory animals	Adult (3-5 month old) C57BL/6J and STOCK Tg(Thy1-EGFP)MJrs/J (hemizygous) (Jackson #007788) mice were used for in vivo microinjection and or/perfusion experiments. 5-7 day old C57BL/6J, STOCK Tg(Thy1-EGFP)MJrs/J (hemizygous) (Jackson #007788) or PSD95-HaloTag mice (homozygous or heterozygous) (courtesy of Seth G.N. Grant, University of Edinburgh) were used to prepare organotypic hippocampal slice cultures. Mice of either sex were used interchangeably to demonstrate the technology.
Wild animals	No wild animals were used in this study.
Field-collected samples	The study did not involve any field-collected samples.
Ethics oversight	Animal procedures were performed in accordance with national law (BGBLA 114 and Directive 522), European Directive 2010/63/EU and institutional guidelines for animal experimentation and were approved by the Austrian Federal Ministry for Education, Science and Research (authorizations BMBWF-V/Sb: 2020-0.363.126 and 2021-0.547.215). Experiments performed on cultured organotypic brain slices involved organ extraction after killing the animal, which does not require ethics authorization.

Note that full information on the approval of the study protocol must also be provided in the manuscript.

Human research participants

Policy information about studies involving human research participants

Population characteristics	For display in Fig. 6a-c, rCATS data from one male individual (35y), undergoing surgery for epilepsy treatment, was used. Fig. 6d, as well as Suppl. Fig. 25 display rCATS data from one archival human FFPE autopsy specimen (35y, female) without brain pathology.<br>In Fig. 6e-i, as well as Suppl. Fig. 26, FFPE biopsy tissue from one patient (53 y, female) diagnosed with MOGAD is displayed.

	Fig. 6j,k as well as Suppl. Fig. 27, display tissue from one human peripheral nerve biopsy tissue (44y, female).<br>Suppl. Fig. 24 displays data from a male patient (36y) who had previously undergone brain surgery for neoplastic disease. In a second, independent surgery for epilepsy of the temporal lobe with sclerosis, the material used in the present study was collected.
Recruitment	The human material used in the present study had been previously collected and stored.<br>Brain sections from 8 individuals were used for rCATS analysis of epilepsy surgery specimens. For display in the manuscript, two of these were selected according to structural preservation and image quality (see Fig. 6a-c, Suppl. Fig. 24).<br>FFPE-fixed brain sections from 2 autopsy specimens, as well as FFPE-fixed biopsy material from one patient diagnosed with MOGAD, were used. In addition, multiple sections from one human FFPE peripheral nerve biopsy were used.
Ethics oversight	Procedures involving human surgery specimens were approved by the Ethics Committee of the Medical University Vienna (authorization EK 1188/2019 and EK2271/2021). Patients provided informed consent for use of brain tissue material. Human archival autopsy and biopsy material from formalin-fixed paraffin-embedded (FFPE) brain and nerve tissue was identified at the Neurobiobank of the Division of Neuropathology and Neurochemistry, Department of Neurology, Medical University of Vienna. Research use of these samples is approved by the Ethics Committee of the Medical University of Vienna, EK 1123/2015 and EK 1636/2019 that provides a common broad consent (biobank consent) according to the Austrian Research Organisation Act 2018, §2d, para 3 (biomaterial can be used within an entire research area, as long as the patient has not withdrawn).

Note that full information on the approval of the study protocol must also be provided in the manuscript.
